# Knowledge about tuberculosis and infection prevention behavior: A nine city longitudinal study from India

**DOI:** 10.1371/journal.pone.0206245

**Published:** 2018-10-30

**Authors:** Sophie Huddart, Thomas Bossuroy, Vincent Pons, Siddhartha Baral, Madhukar Pai, Clara Delavallade

**Affiliations:** 1 Department of Epidemiology, Biostatistics and Occupational Health, McGill University, Montreal, Quebec, Canada; 2 McGill International TB Centre, Montreal, Quebec, Canada; 3 World Bank, Washington DC, District of Columbia, United States of America; 4 Harvard Business School, Boston, Massachusetts, United States of America; 5 International Food Policy Research Institute, Washington DC, District of Columbia, United States of America; Indian Institute of Technology Delhi, INDIA

## Abstract

**Background:**

Improving patients’ tuberculosis (TB) knowledge is a salient component of TB control strategies. Patient knowledge of TB may encourage infection prevention behaviors and improve treatment adherence. The purpose of this study is to examine how TB knowledge and infection prevention behaviors change over the course of treatment.

**Methods:**

A matched patient-health worker dataset (n = 6,031) of publicly treated TB patients with NGO-provided treatment support health workers was compiled in nine Indian cities from March 2013 to September 2014. At the beginning and end of TB treatment, patients were asked about their knowledge of TB symptoms, transmission, and treatment and infection prevention behaviors.

**Results:**

Patients beginning TB treatment (n = 3,424) demonstrated moderate knowledge of TB; 52.5% (50.8%, 54.2%) knew that cough was a symptom of TB and 67.2% (65.6%, 68.7%) knew that TB was communicable. Overall patient knowledge was significantly associated with literacy, education, and income, and was higher at the end of treatment than at the beginning (3.7%, CI: 3.02%, 4.47%). Infection prevention behaviors like covering a cough (63.4%, CI: 61.2%, 65.0%) and sleeping separately (19.3%, CI: 18.0%, 20.7%) were less prevalent. The age difference between patient and health worker as well as a shared language significantly predicted patient knowledge and adherence to infection prevention behaviors.

**Conclusions:**

Social proximity between health worker and patients predicted greater knowledge and adherence to infection prevention behaviors but the latter rate remains undesirably low.

## Introduction

India bears almost 25% of the global tuberculosis (TB) burden with an estimated 2,800,000 new cases in 2016 [1]. Limiting the transmission of TB in the community is critical to curbing the spread of TB. Transmission of TB takes place mostly within the household [2], but also outside [3].

Improving patient knowledge of TB is an important component of enhancing patient-centric care and is a major goal of the End TB strategy [4]. Health workers are often the primary source of information on TB for patients. Effective communication on the cause of tuberculosis, its symptoms, its treatment, and mode of spread is particularly important, especially in countries like India where the disease is still heavily stigmatized [5]. It is assumed that the extended contact with the healthcare system required by lengthy TB treatment will present an opportunity to increase patient knowledge empowering them to reduce risk of spread, adhere to the full treatment course and potentially recognize symptoms of TB in others. Health workers who are socially proximal to their patients, e.g. share a language or are close in age, may be more effective at educating patients than health workers who are very different socially from their patients. Adequate TB knowledge, particularly regarding how TB is spread, may encourage infection prevention behaviors like covering a cough.

A comparison of patients’ knowledge at the beginning and end of treatment allows us to assess whether completing treatment is associated with improved TB knowledge and behavior changes. Existing studies of TB knowledge among Indian TB patients have generally shown a low level of knowledge. Limited general knowledge about TB may be related to the stigmatized nature of TB in some Indian communities. Most studies focused on qualitative assessments, which, while informative, cover limited geographical areas, are restricted to small sample sizes that do not necessarily generalize to larger patient groups [6], and are not suitable for analyzing changes in TB knowledge over the course of treatment due to their cross-sectional nature [7–12]. Further, the literature has not yet explored how patients’ knowledge of infection mechanisms translates into preventive behavior, an important determinant of disease transmission.

The purpose of this study was to examine how TB knowledge and infection prevention behavior change over the course of the TB treatment. The analysis used a large, unique dataset of 6,031 patients surveyed at the beginning and at the end of their treatment in nine different cities in India, where patient data was matched to the data on the 86 health workers who oversaw patient treatment. This large cohort studied at two time-points presented a unique opportunity to assess TB patient knowledge and key infection prevention behaviors, and their evolution through the course of treatment.

## Methods

The study cohort was assembled during two randomized field experiments conducted in partnership with the Jameel Poverty Action Lab (J-PAL) and Operation ASHA [13,14]. Operation ASHA is an Indian NGO that supports TB patients through DOTS (Directly Observed Treatment, Short course), the standard TB treatment regimen, under public-private partnerships with State Governments in India. Patients were assigned to a DOTS center where they would receive their medicine from a DOTS provider and receive counseling from a health worker who shared information on tuberculosis diagnosis and treatment, in addition to monitoring and following-up on treatment adherence. For the purpose of the study, patients were interviewed at the beginning (entry) and end of DOTS (exit) by an independent research organization, using an in-depth survey with extensive questions regarding TB knowledge and related health behaviors.

The cohort was composed of child and adult drug-sensitive tuberculosis patients treated in the public sector in nine cities across four North Indian states (Madhya Pradesh, Delhi, Chhattisgarh and Odisha) from March 2013 to September 2014. All patients starting their treatment as well as all patients who had completed six months of treatment or had interrupted their treatment (based on Operation ASHA monthly reports) were identified and scheduled for interview. A total of 6,031 patients were interviewed at least once through this project using a structured survey.

The surveys administered to patients beginning and completing DOTS contained identical questions on TB knowledge and health behavior. To elicit knowledge of TB symptoms, patients were asked “What symptoms of TB do you know?” Answers coded as correct were weight loss, loss of energy, poor appetite, fever, cough, and night sweats. Similarly, to elicit patient’s knowledge of modes of transmission of TB, patients were asked “How can one contract TB?” Patients were not prompted but interviewers were provided with pre-coded answers like “when another person coughs or sneezes” and “by consuming alcohol” on which to map patient answers. Additional TB knowledge including availability and efficacy of treatment was assessed by asking respondents whether they agreed with statements like “TB is hereditary” and “TB is caused by a germ.” Finally, patients were asked in a yes/no format “Have you been sleeping in another room separate from other household members since being diagnosed?” Because living conditions may limit patients’ ability to alter sleeping arrangements, we use an additional proxy for contagion prevention behavior by asking patients “Do you cover your mouth while coughing to prevent the further spread of TB?”. The survey knowledge questions were used to form an 18-point scale where each correct answer such as knowing cough is a symptom of TB or knowing that TB can cause death is worth one point on the scale. A complete list of knowledge concepts can be seen in the appendix. This knowledge index was normally distributed and had a Cronbach’s α of 0.79, indicating high internal validity [15].

The surveys also collected patient demographic information including gender, age, religion, caste, literacy, education, marital status, work status, BCG vaccination status, household size, income generating activity, personal and familial history of TB, language, and monthly income.

In addition to the patient survey, 86 health workers under Operation ASHA supervision were interviewed and their information was linked to the patient data such that each patient survey record was now linked to the characteristics of the health worker assigned to that patient’s treatment. During the patients’ treatment, health workers shared information on diagnosis and treatment, in addition to monitoring and following up on patients to promote treatment adherence. Health worker covariates included age, education, and training. We constructed social proximity indices, based on the absolute age difference between the patient and his or her health worker, as well as on whether the health worker and patient shared the same language and/or caste. Social proximity in networks has indeed been shown to lower the cost of accessing knowledge between members [16,17].

Continuous and high order categorical covariates were categorized *a priori*. Given the limited 2% rate of missing data, a single chained imputation with 10 iterations was performed to impute missing patient and health worker data.

The analysis involved three goals: 1) assessing patients’ knowledge about TB symptoms, treatment, and transmission mechanisms at the beginning and end of DOTS; 2) assessing patients’ behavior regarding TB infection prevention; and 3) identifying changes in TB knowledge and behavior over the course of the DOTS treatment as well as their socio-demographic predictors.

For the first two goals, the percentages of patients answering TB knowledge questions correctly, or indicating participation in infection preventive behaviors, were calculated at the beginning and end of DOTS. Models were then fit for each knowledge question and preventive behavior to identify the main predictors. For the third goal, modelling was used to measure the association between completing treatment, patient and health worker demographics, and the outcomes of interest. The general knowledge index was used as an outcome to assess associations with overall TB knowledge.

For all models, potentially relevant covariates were identified in univariate analysis using Gaussian or binomial generalized linear models as appropriate with a threshold p-value of 0.25. Religion and caste were found to be highly co-linear (Pearson’s χ^2^ = 3528.2, df = 12, p-value < 0.001) thus, only caste was considered for inclusion in the multivariate model. All potentially relevant covariates were fit into the multivariate model. Generalized estimating equations (GEEs) with robust sandwich estimators were used for the multivariate analysis to account for the correlation created by patients surveyed twice. Patients surveyed twice were given a weight of 0.5 and demographics from the entry survey were used, while singly surveyed patients were given a weight of 1.

For the general knowledge model, the index was scaled to a 100-point scale for ease of interpretation. The general knowledge model was fit using a Gaussian GEE model while all other models were binomial GEEs. All analyses were performed in R.

This project complies with all relevant guidelines and regulations and ethical approval was obtained from the institutional review boards of the Massachusetts Institute of Technology (USA), University of Cape Town (South Africa), and the Institute for Financial Management and Research (India). A simple language consent form was read to all participants and written informed consent denoted by a signature was collected from all interviewed patients and health workers.

## Results

### Sample description

All patients who began and/or completed DOTS treatment during the study period were scheduled for survey interviews upon beginning and/or completing their treatment. A total of 11,594 surveys were scheduled and 8,295 (71.5%) were conducted. The reasons that some surveys were not able to be conducted include respondent refusal, respondents changing places of residence, respondent death, and health workers’ advice against interviewing some sensitive patients. A further 240 (2.1%) surveys were excluded due to missing patient or linked health worker information. In total, 8,055 unique survey responses from 6,031 patients are included in this work.

Of the 8,055 survey responses, 1,400 are from patients who were interviewed only at the beginning of DOTS, 2,024 who were interviewed at the beginning and end of DOTS, and 2,607 who were interviewed only at the end of DOTS. In total there are 3,424 patient surveys from the beginning of DOTS and 4,631 surveys from the end of DOTS.

For all models in this paper, patients who provided both an entry and exit survey data points were treated as a cluster and the correlation between these responses was accounted for using generalized estimating equation models with robust sandwich estimators.

Patient demographics were very similar between the patients interviewed at the beginning and end of DOTS treatment (Table 1). The average age in the cohort was 33.7 years for both interview time-points. About 58% of patients were male and most adult patients were married (65.7% at both time-points). While almost two thirds were working at entry (63.5%), the rate dropped to just above half (52.2%) at the end of treatment. The level of education was low: a third of the patients were illiterate and only a small proportion of patients had received a high school diploma (~ 4%). Only 15% of patients earned more than 10,000 INR a month, approximately 150 USD.

**Table 1:** Demographic summary of patient surveys at beginning and end of DOTS treatment. For marriage, the denominator for the percentage is the number of adults.

		Entry Survey (n = 3424)	Exit Survey (n = 4631)
Average Age, n (mean age)	Adults (>14 years)	3129 (35.8)	4205 (36.1)
	Children (<=14 years)	295 (10.6)	426 (10.6)
Overall mean age (SD)		33.7 (16.3)	33.7 (16.3)
Gender, n (%)	Male	2002 (58.5)	2658 (57.4)
	Female	1422 (41.5)	1973 (42.6)
Religion, n (%)	Christian	22 (0.6)	28 (0.0)
	Hindu	37 (1.1)	3767 (81.3)
	Muslim	37 (1.1)	785 (17.0)
	Other	37 (1.1)	51 (1.1)
Literacy, n (%)	Illiterate	1058 (30.9)	1360 (29.4)
	Read not write	131 (3.8)	153 (3.3)
	Literate	2235 (65.3)	3118 (67.3)
Marriage status, n (%)	Single	1086 (34.2)	1443 (34.3)
	Married	2084 (65.7)	2762 (65.7)
Total household size, mean (SD)		4.6 (2.3)	4.6 (2.2)
Currently working, n (%)	Yes	2175 (63.5)	2422 (52.2)
	No	1249 (36.4)	2209 (47.7)
Number of family members with TB, mean (SD)		0.5 (0.9)	0.5 (.9)
BCG Status	Not vaccinated	2446 (71.4)	3355 (72.4)
	Not sure	69 (2.0)	98 (2.0)
	Vaccinated	909 (26.5)	1178 (25.4)
History of TB, n (%)	Yes	836 (24.4)	1128 (24.4)
	No	2588 (75.6)	3508 (75.6)
Highest education, n (%)	Class 1–5	961 (28.1)	1340 (28.9)
	Class 6–12	1472 (43.0)	2067 (44.6)
	Graduate or other diploma	129 (3.8)	203 (4.4)
	No formal education	862 (25.2)	1021 (22.0)
Monthly Income (INR), n (%)	<5,000	1642 (48.0)	2158 (46.6)
	5000–10000	1252 (36.6)	1751 (37.8)
	>10000	530 (15.5)	722 (15.6)

### Knowledge and behavior at the onset of the treatment

Table 2 provides summary statistics of individual TB knowledge concepts and infection prevention behaviors at the onset of TB treatment.

**Table 2:** Summary of TB knowledge rates and health behaviors at the beginning of DOTS.

		Proportion, 95% CI
**Knowledge of TB transmission**		
TB is communicable	Male	69.8 (67.7, 71.8)
	Female	63.5 (61.0, 66.0)
	Overall	67.2 (65.6, 68.7)
TB spreads though resp. droplets	Male	79.2 (77.4, 80.9)
	Female	74.6 (72.3, 76.8)
	Overall	77.3 (75.9, 78.7)
**Knowledge of TB treatment**		
TB drugs available	Male	93.7 (92.5, 94.7)
	Female	92.9 (91.4, 94.1)
	Overall	93.3 (92.5, 94.2)
TB regimen is 6–8 months	Male	94.9 (93.8, 95.8)
	Female	93.6 (92.2, 94.8)
	Overall	94.3 (93.5, 95.0)
TB drugs should be continued after symptoms subside	Male	87.0 (85.4, 88.4)
	Female	87.8 (86.0, 89.3)
	Overall	87.3 (86.1, 88.4)
Failure to complete regimen can lead to more severe TB infection	Male	92.2 (91.0, 93.3)
	Female	92.4 (90.9, 93.7)
	Overall	92.3 (91.3, 93.2)
**Knowledge of TB symptoms**		
Cough as symptom	Male	58.0 (55.8,60.2)
	Female	44.9 (42.3,47.5)
	Overall	52.5 (50.8, 54.2)
Fever as symptom	Male	36.6 (34.5, 38.7)
	Female	29.6 (27.2, 32.0)
	Overall	33.8 (32.1, 35.3)
**TB Transmission Behavior**		
Sleep in separate room	Male	25.5 (23.6, 27.5)
	Female	10.6 (9.1, 12.3)
	Overall	19.3 (18.0, 20.7)
Cover mouth with hand or cloth when coughing	Male	65.0 (62.9, 67.0)
	Female	61.2 (58.6, 63.7)
	Overall	63.4 (61.2, 65.0)

Knowledge regarding spread of TB was relatively high. Three quarters of patients could identify droplets released by coughing or sneezing as a mode of transmission (77.3%, CI: 75.9%, 78.7%). This is higher than in other studies of Indian people without TB where only 20%–56% could correctly identify the mode of transmission.[7–9] About two thirds knew that TB is communicable (67.2%, CI: 65.6%, 68.7%), a slightly lower share likely due to some confusion over the definition of a communicable disease. Knowledge about TB symptoms was more limited though. Upon entering treatment, about half of the patients could identify the major symptom of TB: cough (52.5%, CI: 50.8%, 54.2%). One third of the patients (33.8%, CI: 32.1%, 35.3%) knew fever was a symptom of TB.

Knowledge levels about TB treatment among patients were very high, reflecting effective dissemination of information after diagnosis. At the beginning of DOTS, most patients knew that drugs were available to treat TB (93.3%, CI: 92.5%, 94.2%), that the length of the treatment regimen was 6–8 months (94.3%, CI: 93.5%, 95.0%), that TB medicines should be continued even after symptoms subsided (87.3%, CI: 86.1%, 88.4%), and that non-adherence to treatment could complicate their TB (92.3%, CI: 91.3%, 93.2%). Other studies, covering general populations in rural India, found 68% of people knew that TB is curable[10] but only 7% of people know that 6–8 months of treatment was required.[11]

All in all, patients started their treatment with relatively high levels of *knowledge* about TB and mode of spread.

By contrast, reported use of infection prevention *behaviors* were much lower. Less than 20.0% (19.3%, CI: 18.0%, 20.7%) of patients reported sleeping in a separate room from other household members, likely reflecting constraints to living arrangements in urban slums. More strikingly, only 63.4% (CI: 61.2%, 65.0%) of patients reported covering their mouth when they coughed at the onset of the treatment.

### Determinants and evolution of knowledge

Modelling was used to investigate patient and health worker covariates that associated with higher rates of TB knowledge.

#### General knowledge

The average TB knowledge score of patients at the beginning of DOTS was 54.93 (54.33, 55.53) and the average TB knowledge score of patients at the end of DOTS was significantly higher (p< 0.00) at 59.32 (58.83, 59.81).

After adjustment by the other model covariates, the knowledge score was still significantly higher by 3.75% (3.02%, 4.48%) for patients at the end of treatment when compared to patients at beginning of treatment (Table 3). Higher education and socio-economic status were associated with higher knowledge of TB, as reflected in the positive coefficients attached to age, being male, marriage, literacy, education, and monthly income. Older patients were also significantly more likely to have a higher knowledge score. Patients who reported having a family member with TB or a personal history of TB also displayed higher TB knowledge. Being counseled by an older health worker was associated with higher knowledge scores, but knowledge was negatively associated with the age gap between health worker and patient. Sharing a common language with the health worker had one of the strongest associations with TB knowledge in the model (8.18%, CI: 6.13%, 10.22%). The last two results suggest that social proximity between patient and health worker significantly improves transmission of TB-related knowledge.

**Table 3:** Patient and health worker characteristics associated with general knowledge index, n = 8,055. The 18-point index was scaled to 100 points.

	Coefficient	Confidence Interval	Significance
Beginning of treatment	Reference		
End of treatment	3.75	(3.02, 4.48)	*
Age (years)	0.08	(0.04, 0.11)	*
Female	Reference		
Male	2.25	(1.44, 3.05)	*
General Caste	Reference		
Other Backwards Caste	-0.50	(-1.55, 0.55)	
Scheduled Caste	0.24	(-0.94, 1.42)	
Scheduled Tribe	0.76	(-0.78, 2.29)	
Minority	-2.16	(-3.82, -0.5)	*
Illiterate	Reference		
Read not write	3.29	(0.81, 5.76)	*
Literate	3.20	(1.74, 4.66)	*
Single	Reference		
Married	0.98	(0.04, 1.92)	*
Total household size	-0.20	(-0.37, -0.02)	*
Number family w/ TB	1.41	(0.96, 1.85)	*
No history of TB	Reference		
History of TB	3.44	(2.57, 4.31)	*
Hindi	Reference		
Other	5.28	(2.75, 7.81)	*
No formal education	Reference		
Class 1–5	2.54	(1.01, 4.07)	*
Class 6–12	7.53	(5.78, 9.27)	*
High school graduate or other diploma	14.62	(12.42, 16.83)	*
<5,000 INR/month	Reference		
<10,000 INR/month	1.23	(0.39, 2.07)	*
>10,000 INR/month	3.43	(2.33, 4.53)	*
Health worker age (years)	0.16	(0.11, 0.22)	*
Absolute age difference w/ health worker (years)	-0.67	(-0.27, -0.18)	*
Health worker does not share language	Reference		
Health worker does share language	8.18	(6.13, 10.22)	*
Health worker, no diploma	Reference		
Health worker, high school diploma	0.31	(-0.68, 1.30)	
Health worker, at least some university	0.83	(-0.15, 1.82)	
Health worker does not share caste	Reference		
Health worker does share caste	-0.67	(-1.54, 0.20)	

*indicates term was statistically significant

#### Knowledge of how TB is spread

Patients at the end of treatment had significantly higher knowledge about TB mode of spread than patients at the beginning of TB treatment. They had 28% (OR 1.28, CI: 1.15, 1.41) higher odds of knowing that TB is communicable by the end of DOTS (Table 4) and 53% (OR 1.53, CI: 1.37, 1.71) higher odds of knowing that TB spreads through respiratory droplets (Table 5).

**Table 4:** Patient and health worker characteristics associated with knowing that TB is communicable, n = 8,055.

	Odds Ratio	Confidence Interval	Significance
Beginning of treatment	Reference		
End of treatment	1.28	(1.15, 1.41)	*
Age (years)	1.01	(1.00, 1.01)	*
Female	Reference		
Male	1.26	(1.13, 1.41)	*
Illiterate	Reference		
Read not write	1.70	(1.25, 2.31)	*
Literate	1.41	(1.17, 1.70)	*
Single	Reference		
Married	1.11	(0.97, 1.27)	
Total household size	0.99	(0.96, 1.01)	
Number family w/ TB	1.15	(1.08, 1.22)	*
No BCG	Reference		
Not sure	1.31	(0.86, 2.00)	
BCG vaccinated	0.90	(0.80, 1.02)	
No history of TB	Reference		
History of TB	1.06	(0.93, 1.21)	
Hindi	Reference		
Other	1.85	(1.36, 2.53)	*
No formal education	Reference		
Class 1–5	1.04	(0.86, 1.25)	
Class 6–12	1.69	(1.35, 2.12)	*
High school graduate or other diploma	2.61	(1.76, 3.87)	*
<5,000 INR/month	Reference		
<10,000 INR/month	1.09	(0.97, 1.23)	
>10,000 INR/month	1.01	(0.86, 1.19)	
Health worker age (years)	1.02	(1.01, 1.03)	*
Absolute age difference w/ health worker (years)	0.98	(0.98, 0.99)	*
Health worker does not share language	Reference		
Health worker does share language	1.84	(1.45, 2.32)	*
Health worker, no diploma	Reference		
Health worker, high school diploma	1.31	(1.12, 1.52)	*
Health worker, at least some university	1.11	(0.97, 1.27)	
Health worker does not share caste	Reference		
Health work does share caste	0.92	(0.82, 1.03)	

*indicates term was statistically significant

**Table 5:** Patient and health worker characteristics associated with knowing that TB is spread through respiratory droplets, n = 8,055.

	Odds Ratio	Confidence Interval	Significance
Beginning of treatment	Reference		
End of treatment	1.53	(1.37, 1.71)	*
Age (years)	1.01	(1.00, 1.01)	*
Female	Reference		
Male	1.11	(0.97, 1.27)	
General Caste	Reference		
Other Backwards Caste	0.96	(0.81, 1.14)	
Scheduled Caste	1.17	(0.96, 1.42)	
Scheduled Tribe	1.10	(0.86, 1.41)	
Minority	0.80	(0.62, 1.03)	
Illiterate	Reference		
Read not write	1.26	(0.92, 1.72)	
Literate	1.55	(1.27, 1.91)	*
Not currently working	Reference		
Currently working	0.97	(0.85, 1.11)	
Number family w/ TB	1.15	(1.07, 1.24)	*
No BCG	Reference		
Not sure	2.06	(1.21, 3.51)	*
BCG vaccinated	1.00	(0.87, 1.15)	
No history of TB	Reference		
History of TB	1.29	(1.12, 1.50)	*
Hindi	Reference		
Other	0.96	(0.69, 1.34)	
No formal education	Reference		
Class 1–5	1.08	(0.88, 1.31)	
Class 6–12	1.80	(1.40, 2.30)	*
High school graduate or other diploma	2.87	(1.77, 4.65)	*
<5,000 INR/month	Reference		
<10,0000 INR/month	1.04	(0.91, 1.18)	
>10,000 INR/month	1.29	(1.07, 1.56)	*
Health worker age (years)	1.01	(1.00, 1.02)	*
Absolute age difference w/ health worker (years)	0.98	(0.98, 0.99)	*
Health worker does not share language	Reference		
Health worker does share language	1.56	(1.21, 2.03)	*
Health worker, no diploma	Reference		
Health worker, high school diploma	1.20	(1.01, 1.42)	*
Health worker, at least some university	1.06	(0.90, 1.23)	
Health worker does not share caste	Reference		
Health work does share caste	0.93	(0.81, 1.07)	

*indicates term was statistically significant

Specific knowledge that TB is communicable was associated with being male, literate, and educated (Table 4). Health worker’s education mattered too. Patients whose treatment is supervised by a health worker with a high school diploma had 31% (OR 1.31, CI: 1.12, 1.52) higher odds of knowing that TB is communicable. In addition, social proximity between patient and health worker is a strong predictor of knowledge of how TB is spread. Patients with health workers close in age and who spoke the same language were more likely to know that TB is communicable. Sharing the same language increases knowledge odds by 84% (OR 1.84, CI: 1.45, 2.32).

Knowledge that respiratory droplets are the mechanism by which TB spreads shows similar patterns. It is also strongly associated with literacy and education but not gender (Table 5). Literate patients are 55% (OR 1.55, CI: 1.27, 1.91) more likely to know about this mode of infection. The difference in knowledge odds between uneducated patients and patients with a graduate or similar diploma is even higher (OR: 2.87, CI: 1.77, 4.65). Health worker predictors again included age and high school diploma. Finally, sharing the same language increases the odds of knowing about the mode of spread by 56% (OR 1.56, CI: 1.21, 2.03).

### Determinants and evolution of infection prevention behaviors

Similar modelling was used to investigate the determinants of adoption of infection prevention behaviors.

#### Behavior preventing spread of TB

At the end of the DOTS treatment, patients were neither more likely to sleep separately (Table 6), which would reduce household contact transmission, nor more likely to cover a cough, which would reduce household and non-household contact transmission (Table 7). The lack of improvement in prevention behavior contrasts with the improvement of knowledge about infection mechanisms over the course of DOTS.

**Table 6:** Patient and health worker characteristics associated with the patient sleeping separately, n = 8,055.

	Odds Ratio	Confidence Interval	Significance
Beginning of treatment	Reference		
End of treatment	0.98	(0.83, 1.11)	
Age (years)	1.05	(1.05, 1.06)	*
Female	Reference		
Male	2.09	(1.79, 2.43)	*
Illiterate	Reference		
Read not write	1.34	(0.95, 1.87)	
Literate	1.48	(1.27, 1.72)	*
Single	Reference		
Married	0.36	(0.30, 0.42)	*
Total household size	1.04	(1.01, 1.07)	*
Not currently working	Reference		
Working	1.27	(1.10, 1.46)	*
Number family w/ TB	0.87	(0.81, 0.95)	
Hindi	Reference		
Other	1.56	(1.22, 2.00)	*
Health worker age (years)	0.99	(0.98, 1.00)	*
Absolute age difference w/ health worker (years)	0.98	(0.97, 0.99)	*
Health worker, no diploma	Reference		
Health worker, high school diploma	0.69	(0.58, 0.82)	*
Health worker, at least some university	0.83	(0.70, 0.98)	*

*indicates term was statistically significant

**Table 7:** Patient and health worker characteristics associated with the patients covering their mouths while coughing, n = 8,055.

	Odds Ratio	Confidence Interval	Significance
Beginning of treatment	Reference		
End of treatment	0.98	(0.89, 1.08)	
Age (years)	1.01	(1.00, 1.01)	*
Female	Reference		
Male	1.21	(1.09, 1.36)	*
Illiterate	Reference		
Read not write	0.93	(0.71, 1.22)	
Literate	1.10	(0.98, 1.23)	
Single	Reference		
Married	0.97	(0.86, 1.10)	
Not currently working	Reference		
Working	1.11	(1.00,1.24)	*
Number family w/ TB	1.07	(1.02, 1.14)	*
No history of TB	Reference		
History of TB	1.58	(1.40,1.78)	*
Hindi	Reference		
Other	0.74	(0.55, 1.00)	*
Health worker age (years)	1.00	(0.99, 1.00)	*
Absolute age difference w/ health worker (years)	0.98	(0.98, 0.99)	*
Health worker does not share language	Reference		
Health worker does share language	1.53	(1.21,1.94)	*

*indicates term was statistically significant

Sleeping alone was significantly associated with indicators of socio-economic attainment such as literacy, labor force participation, age, and being male. Being married however was strongly associated with not sleeping alone, with an OR of 0.36 (0.30, 0.42), most likely because spouses keep sharing a room. Those with family members who have TB were less likely to sleep alone, perhaps because TB patients in the household were sharing a room, or possibly because having several household members infected with TB is associated with poverty and overcrowding. Yet the social proximity of health workers to the patients still seems to make a difference, with the age gap significantly and negatively associated with sleeping alone.

Similarly, socio-economic status is associated with a higher probability of covering mouth when coughing. Personal or family history of TB display a strong association with more preventive behavior limiting infection risk for contacts, suggesting that patients learn from experience. Strikingly, greater social proximity between patient and health worker in charge of counseling them along their treatment was associated with more protective behavior in the patient towards their contacts. Patient and health worker speaking the same language translates into a 1.53 OR (1.21, 1.94) of covering the mouth when coughing.

## Discussion

The results of this study expand the available evidence on TB knowledge and infection prevention among patients.

First, at the onset of the DOTS treatment, overall knowledge about TB is moderate in this population and varies across knowledge dimensions. **Patients may have acquired this initial knowledge during the process of care-seeking and TB diagnosis**. Patients know more about TB treatment than they know about symptoms. Aspects related to the availability and length of treatment, as well as the importance of completing the entire treatment are being effectively communicated to the large majority of TB patients. Our estimates of Indian TB patient knowledge are higher than some others in the literature [7–12]; perhaps because this cohort represented urban TB patients whereas most available knowledge, attitude, and belief assessments regarding TB were conducted on non-TB populations in rural areas. Though rates of TB knowledge were higher than in other studies even at the beginning of DOTS, TB patients may have acquired additional TB knowledge as they progressed through the patient pathway before being enrolled in DOTS.

Despite relatively high knowledge of how TB is spread, a large fraction of patients are not engaging in behaviors preventing the spread of TB either to close contacts or to contacts outside the household. This suggests that there is substantial room for improvement in patients’ adoption of infection prevention behaviors. The low rates of patients sleeping separately to prevent disease spread within the household may however reflect the often-overcrowded living conditions of TB patients where many families may not have separate sleeping quarters available.

Thanks to the comprehensive survey information collected, our study documents predictors significantly associated with infection prevention knowledge and behavior. For most indicators, drivers of knowledge and behavior are comparable in magnitude as well as ordinally. Patients with higher socio-economic status, as measured by education, labor force participation or income, know significantly more about infection mechanisms and are more likely to adopt preventive behaviors.

Our results consistently point to the capacity of patients to learn from their experience with the disease. Having personal or family history of TB is associated by greater knowledge and prevention behaviors, and knowledge is also significantly higher at the end of the treatment as opposed to the beginning. This may be the result of efficient counseling by health workers or DOTS providers.

Leveraging matched patient-health worker data, our study offers the opportunity to investigate how a patient’s knowledge and behavior may be affected by the socio-demographic characteristics of the health worker in charge of his/her treatment. A key result is that social proximity (as defined by closeness in age and language) critically improves transmission of knowledge from health worker to patient, as well as preventive behavior in patients, especially covering cough.

Finally, our study does not capture any change in the adoption by patients of prevention behaviors that would limit TB contagion to contacts. Despite initially low levels of preventive behavior adoption rates, there is no significant change over the course of the DOTS treatment. This may be due to a failure of the health system to promote desirable behaviors in practice, or to the fact that patients by the end of their treatment are not contagious and therefore less likely to report prevention behaviors. Additional longitudinal TB knowledge surveys that cover the contagious period are needed to describe whether patients receiving DOTS are engaging in transmission prevention behavior when it is most important.

This analysis capitalizes on the large cohort of patients surveyed to provide precise estimates of patients’ TB knowledge rates and the rates of key infection prevention behaviors. The knowledge index created from the survey responses, while not externally validated, captured information on patients’ knowledge of TB symptoms, modes of transmission, and treatment. Uniquely, we were able to match patient-level data with health worker data and show that social proximity is consistently associated with higher knowledge and greater adoption of prevention behavior. The two time-points also allowed us to compare the evolution of TB knowledge and related health behaviors over the course of the DOTS treatment to estimate associated changes in these rates. These characteristics are unique among similar studies in the Indian TB context.

Without a control group of individuals not receiving DOTS and treatment counselling, causal inference about the impact of the DOTS treatment may not reliably be drawn from our analysis. We do, however, provide evidence on the correlation between patients’ and health workers’ socio-demographic characteristics and patient’s infection-related knowledge and behavior. The survey tool used for this work did not collect data on knowledge regarding other communicable diseases so we are unable to benchmark these results against patients’ awareness of other diseases. Our survey data was not linked to the patients’ medical records precluding questions about how patient knowledge interacts with the type of TB, other comorbidities and outcome of treatment. Additionally, our survey tool did not measure how interested individual patients were in learning about TB and prevention behaviors.

Our findings provide useful insights for further research and policy. They indeed suggest that the lack of knowledge and adoption of prevention behaviors is concentrated in populations with low socio-economic status, which may inform efforts to better target counseling and follow-up efforts. Results also suggest that patients do learn about the disease and the mechanisms of infection from their interaction with health workers or DOTS providers. However, we show more limited effectiveness of communication around adequate actions to be taken. Reinforcing communication on vital information for TB patients is critical to improving quality of care, and additional research is needed to better understand how to effectively catalyze patients’ behavioral change around contagion prevention. Finally, results point to the critical importance of social proximity between the patients and the health workers, as defined by a shared language and a small age difference. Public health systems may have greater impact by strategically recruiting health workers whose characteristics make them closer to the population they serve and therefore more likely to be welcomed and listened to.

## Supporting information

S1 Fig Patient questionnaire. Full questionnaire administered to patients.
(PDF)

## References

[1] World Health Organization. Global Tuberculosis Report. Geneva; 2017.

[2] Otero L, Shah L, Verdonck K, Battaglioli T, Brewer T, Gotuzzo E, et al A prospective longitudinal study of tuberculosis among household contacts of smear-positive tuberculosis cases in Lima, Peru. BMC Infect Dis. BMC Infectious Diseases; 2016;16: 259 10.1186/s12879-016-1616-x 27278655 PMC4898451

[3] Verver S, Warren RM, Munch Z, Richardson M, Van Der Spuy GD, Borgdorff MW, et al Proportion of tuberculosis transmission that takes place in households in a high-incidence area. Lancet. 2004;363: 212–214. 10.1016/S0140-6736(03)15332-9 14738796

[4] World Health Organization. The End TB Strategy: Global Strategy and Targets for Tuberculosis Prevention, Care, and Control After 2015. Geneva; 2015.

[5] Sagili KD, Satyanarayana S, Chadha SS. Is Knowledge Regarding Tuberculosis Associated with Stigmatising and Discriminating Attitudes of General Population towards Tuberculosis Patients? Findings from a Community Based Survey in 30 Districts of India. Subbian S, editor. PLoS One. Public Library of Science; 2016;11: e0147274 10.1371/journal.pone.0147274 26829713 PMC4734597

[6] Ganapathy S, Thomas BE, Jawahar MS, Selvi KJ, Sivasubramaniam, Weiss M. Perceptions of gender and tuberculosis in a south Indian urban community. Indian J Tuberc. 2008;55: 9–14. Public Health and Epidemiology, Health Social Science 18361305

[7] Arora N, Vadrevu R, Chandrasekhar A, Gupta A. Low tuberculosis knowledge among HIV-infected patients in a high HIV prevalence region within southeast India. J Int Assoc Provid AIDS Care. 2013;12: 84–9. 10.1177/1545109712461553 23060464

[8] Sreeramareddy CT, Harsha Kumar HN, Arokiasamy JT. Prevalence of self-reported tuberculosis, knowledge about tuberculosis transmission and its determinants among adults in India: results from a nation-wide cross-sectional household survey. BMC Infect Dis. BMC Infectious Diseases; 2013;13: 16 10.1186/1471-2334-13-16 23324535 PMC3551631

[9] Kar M, Logaraj M. Awareness, Attitude And Treatment Seeking Behaviour Regarding Tuberculosis in a Rural Area Of Tamil Nadu. Indian J Tuberc. 2010;57: 226–229. 21141344

[10] Rao VG, Yadav R, Bhat J, Tiwari BK, Bhondeley MK. Knowledge and attitude towards tuberculosis amongst the tribal population of Jhabua, Madhya Pradesh. Indian J Tuberc. 2012;59: 243–248. 23342547

[11] Yadav S, Mathur M, Dixit A. Knowledge and attitude towards tuberculosis among sandstone quarry workers in desert parts of rajasthan. Indian J Tuberc. 2006;53: 187.

[12] Selvam JM, Wares F, Perumal M, Gopi PG, Sudha G, Chandrasekaran V, et al Health-seeking behaviour of new smear-positive TB patients under a DOTS programme in Tamil Nadu, India, 2003. Int J Tuberc Lung Dis. 2007;11: 161–167. 17263286

[13] Bossuroy T, Delavallade C, Pons V. Biometric Monitoring, Healthcare Delivery and Performance Measurement: Experimental Evidence from Tuberculosis Control in India. 2018.

[14] Bossuroy T, Delavallade C, Pons V. Performance-Based Incentives and the Delivery of Healthcare: Experimental Evidence from the National Tuberculosis Control Program in India. 2018.

[15] Tavakol M, Dennick R. Making sense of Cronbach’s alpha. International Journal of Medical Education. IJME; 27 6 2011 2: 53–55. 10.5116/ijme.4dfb.8dfd 28029643 PMC4205511

[16] Sorenson O, Rivkin JW, Fleming L. Complexity, networks and knowledge flow. Res Policy. 2006;35: 994–1017. 10.1016/j.respol.2006.05.002

[17] Agrawal A, Kapur D, McHale J. How do spatial and social proximity influence knowledge flows? Evidence from patent data. J Urban Econ. 2008;64: 258–269. 10.1016/j.jue.2008.01.003

