# Supplementary material for: Knowledge about tuberculosis and infection prevention behavior: A nine city longitudinal study from India
Source: PLoS One. 2018 Oct 30;13(10):e0206245. doi: 10.1371/journal.pone.0206245 (PMC6207322; doi:10.1371/journal.pone.0206245)

# Evaluation of Operation ASHA modified DOTS model for Tuberculosis treatment

## DOTS Patient Interview - Adult Entry

सर्वेक्षणकर्ता के लिए दिशानिर्देशः सर्वेक्षण के लिए जाने से पहले निम्नलिखित जानकारी भरें। **जिन मरीज़ों का सर्वे आपको करना है उनसे संबंधित जानकारी आपके पर्यवेक्षक आपको देंगे।**

**Surveyor Instructions: fill all the following pieces of information before leaving for the field. Your supervisor will provide you the information regarding the patient(s) you have to survey.**

| | | |
|---|---|---|
| 1 | प्रश्नावली कोड नंबर<br>Questionnaire code | राज्य + शहर + क्षेत्र + मरीज.<br>state + city + center + patient |
| 2 | राज्य का नाम / **कोड**<br>State name/code | ________ |
| 3 | **शहर का नाम / कोड**<br>City name / code | ________ |
| 4 | क्षेत्र का नाम/कोड<br>Area name / code | ________ |
| 5 | मरीज़ का पहला और **अंतिम** नाम/कोड<br>Patient's first & last name / code | ________ |
| 5.1 | **मरीज़ का ती बी नम्बर**<br>Patient TB number | ________ \ |
| 5.2 | **मरीज़ का लेब नम्बर**<br>Patient Lab number | ________ |
| 6 | **सेंटर का नाम / कोड**<br>Center name /code | ________ |
| 7 | **काउंसलर का नाम / कोड**<br>Counselor name/code | ________ |
| 8 | **सर्वेक्षणकर्ता** का नाम/कोड<br>Surveyor name / code | ________ |
| 9 | **पर्यवेक्षक** का नाम/कोड<br>Supervisor name / code | ________ |
| A0.1 | **मरीज़ के घर के भूमंडलीय स्थापन युक्ति निर्देशांक**<br>*GPS Coordinates of patient's house* | North ° .<br>East ° . |
| A.1 | **आपका नाम क्या है ?**<br>What is your name?<br>***सर्वेक्षकः सूचना ध्यानपूर्वक लिखें***<br>*INTERVIEWER: RECORD CAREFULLY* | **नाम**<br>Name: ________<br>**अंतिम नाम**<br>Last name ________<br>**पिता का नाम**<br>Father's Name ________ |
| 10 | **रोगी का पता**/Patient Address: ________<br>________<br>**रोगी का फोन नंबर**/Patient Phone No.: ________ | |

| | | | |
|---|---|---|---|
| 11 | **पहली** विज़िट<br>दिनांक (दिन/महीना/वर्ष)<br>1st visit Date (dd/mm/yy) | दिन/Day [ ][ ] माह/Month [ ][ ] वर्ष/Year [ ][ ]<br>हाँ<br>Yes 1<br>**अपॉइंटमेंट नहीं मिला**<br>No appointment 2 | |
| 12 | **दूसरी** विज़िट 2<br>दिनांक (दिन/महीना/वर्ष)<br>2nd visit Date (dd/mm/yy) | दिन/Day [ ][ ] माह/Month [ ][ ] वर्ष/Year [ ][ ]<br>हाँ<br>Yes 1<br>**अपॉइंटमेंट नहीं मिला**<br>No appointment 2 | |
| 13 | **तीसरी** विज़िट<br>दिनांक (दिन/महीना/वर्ष)<br>3rd visit Date (dd/mm/yy) | दिन/Day [ ][ ] माह/Month [ ][ ] वर्ष/Year [ ][ ]<br>हाँ<br>Yes 1<br>**अपॉइंटमेंट नहीं मिला**<br>No appointment 2 | |
| 14 | सर्वे का परिणाम<br>Outcome of the survey | सर्वे पूरा हुआ।<br>Fully completed 1<br>अधूरा रह गया।<br>Partially completed 2<br>पूरा नहीं हुआः मरीज़ ने जवाब देने से मना कर दिया<br>Not completed: patient refused to answer 3<br>पूरा नहीं हुआः मरीज़ इस पते पर अब नहीं रहता<br>Not completed: patient does not live at this address anymore 4<br>पूरा नहीं हुआ, अन्य कारण **(स्पष्ट करें)**<br>Not completed, other reason (Specify) 5<br>**पूरा नही हुआ, मरीज़ उपलब्ध नही था/अपोइनमेन्ट नही मिला**<br>Not completed: patient refused appointment or was unavailable 6<br>**पूरा नही हुआ, मरीज़ नही मिला**<br>Not completed: patient not found 7<br>**पूरा नही हुआ, काउनसलर ने मना किया**<br>Not completed: counselor warning 8<br>**पूरा नही हुआ, मरीज़ का देहान्त हो गया**<br>Not completed: patient died 9 | |
| 15 | **सर्वे किस भाषा में दिया गया?**<br>In what language was the survey given? | हिन्दी<br>Hindi 1<br>पंजाबी<br>Punjabi 2<br>**अँग्रेज़ी**<br>English 3<br>अन्य (स्पष्ट करें):<br>Other (Specify): -777 | |

**A0: Consent Form** Questionnaire code: |__|__| |__|__| |__|__| |__|__|__|

हम एक शोध अध्ययन संस्था से हैं। अब्दुल लतीफ जमील पोवर्टी एक्शन लैब साउथ एशिया (जे–पाल साउथ एशिया) का संचालन केन्द्र चेन्नई में है। हम आपसे टीबी/क्षय रोग से जुड़े आपके इलाज़ के बारे में कुछ प्रश्न पूछेंगे और आपके परिवार की कुछ निजी जानाकारियां प्राप्त करेंगे। इस सर्वे में हिस्सा लेने से पहले सोच सकते हैं कि आप इस सर्वे में हिस्सा लेना चाहते हैं या नहीं।

यह साक्षात्कार स्वैच्छिक है। अगर आप इस सर्वे में हिस्सा लेने के लिये राज़ी हैं तो भी आप किसी भी सवाल का जवाब देने से मना कर सकते हैं या किसी भी समय आप इस साक्षात्कार में हिस्सा लेने से मना कर सकते हैं।

अगर आप हिस्सा लेने के लिये तैयार हैं तो हम आपसे ऐसा ही दूसरा सर्वे 6 महीने बाद करेंगे। अगर आप चाहें तो उस समय भी इस सर्वे में हिस्सा लेने से मना कर सकते हैं।

जो भी जानकारी आप हमें देगें वह गुप्त रखी जाएगी। हम आपका नाम और जवाब किसी भी अन्य स्वाथ्य कर्मी को नहीं बतायेंगे। आपके जवाब का उपयोग सिर्फ शोध अध्ययन के लिये होगा और आपकी व्यावसायिक स्थिति पर इसका कोई प्रभाव नहीं पड़ेगा। इस सर्वे में आपके हिस्सा लेने के बारे में पुलिस, कोर्ट, सरकार या किसी और को कोई जानकारी नहीं दी जायेगी। हमारा यह शोध कुल तीन साल में पूरा होगा। आज के बाद 3 साल के लिए ये सारी प्रश्नावली हमारे जे–पाल कार्यालयए दिल्ली की अलमारी में रखी जायेंगी और उसके बाद सभी प्रश्नावलियों को नष्ट कर दिया जायेगा।

क्या आपको इस सर्वे की शर्ते मंजूर हैं? और क्या आप इस अध्ययन में अपनी मर्ज़ी से भाग लेना चाहते हैं?

[ ] दिये गये दस्तावेज में वर्णित प्रक्रिया मुझे मंजूर है। मेरे प्रश्नों के जो जवाब दिये गये उससे मैं संतुष्ट हूँ और अपनी मर्ज़ी से इस अध्ययन में हिस्सा लेना चाहताध्चाहती हूँ और मेरे पास इस प्रश्नावली की एक प्रति है।

[ ] , मैं इस प्रश्नावली के जवाबों से सहमत हूँ।

जवाब देने वाले का नाम ‹.......................................› ‹.......................................› ‹.....................................

(पहला नाम) (आख़िरी नाम) (स्थान ;शहर)

जवाब देने वाले के हस्ताक्षर ........................................‹ दिनांक ..........................................

सर्वेक्षणकर्ता के हस्ताक्षर ........................................‹ दिनांक ..........................................

आप कोई भी प्रश्न या जानकारी (आपके नाम और पते की जानकारी) व कोई प्रश्न व सलाह के लिए संपर्क करें।

यदि आपको लगता है कि आपके साथ सही बर्ताव नहीं किया गया, या आप एक शोध उत्तरदाता के रूप में अपने अधिकार से संबंधित कोई प्रश्न पूछना चाहते हैं तो आप हमें संपर्क कर सकते हैं। आप हमें इस नंबर पर संपर्क कर सकते हैं :– शोभिनी मुख़र्जी जेपाल दक्षिण एशिया नई दिल्ली . 0987107155

## A0: Consent Form

Questionnaire code: |__|__| |__|__| |__|__| |__|__|__|

We work for a research organization based in Chennai. We would like to ask you a few questions related to you, your family and your health. Before participating in this survey, please think about whether you wish to participate or not.

• This interview is voluntary. Even after agreeing to take part in this survey, you'll be free to not answer any question you're asked and to end your participation in this survey at any time.

• If you are willing to participate, then we will provide you with a second survey after 6 months. You're free to end your participation in this survey at this time as well.

• Whatever information you provide us with will be kept confidential. We will not disclose your name and responses to any health worker. Your answers will be used for the sake of research only and will not affect your professional status in any way. The police, courts, government, and/or anyone else will not be informed regarding your participation. Our research will take a total of three years. All questionnaires will be kept in a locker in our J-PAL office in Delhi for the next 3 years, after which time all questionnaires will be destroyed.

Do you accept the terms of this survey and do you wish to volunteer in this study?

[ ] I accept the procedures that were described in the given documents. I am satisfied with the answers that I have provided, and I wish to take part in this study of my own accord. I also have a copy of this questionnaire.

[ ] I stand by the answers given in this questionnaire.

Name of the respondent: /____________________/ /_____________________/ /_____________________/
(First name) (Last name) (Place) (City)

Signature of the respondent _____________________________________________ Date _____________________

Signature of the surveyor _____________________________________________ Date _____________________

Please contact us if you have any questions or require any information (regarding your name and address), or if you have any other questions or need any advice.

If you feel you have been treated unfairly, or if you wish to ask any questions regarding your rights as a research subject, you may contact us. You may contact us at this number: Shobhini Mukherjee, JPAL South Asia, New Delhi 09871091555

## A0: Consent Form

Questionnaire code: |__|__| |__|__| |__|__| |__|__|__|

We are from a research organization. The managing center of Abdul Latif Jameel Poverty Action Lab South Asia (J-PAL South Asia) is in Chennai. We would like to ask you a few questions related to your T.B. treatment and obtain some personal information about your family. Before participating in this survey, please think about whether you wish to participate or not.

• This interview is voluntary. Even after agreeing to take part in this survey, you'll be free to not answer any question you're asked and to end your participation in this survey at any time.

• If you are willing to participate, then we will provide you with a second survey after 6 months. You're free to end your participation in this survey at this time as well.

• Whatever information you provide us with will be kept confidential. We will not disclose your name and responses to any health worker. Your answers will be used for the sake of research only and will not affect your professional status in any way. The police, courts, government, and/or anyone else will not be informed regarding your participation. Our research will take a total of three years. All questionnaires will be kept in a locker in our J-PAL office in Delhi for the next 3 years, after which time all questionnaires will be destroyed.

Do you accept the terms of this survey and do you wish to volunteer in this study?

[ ] I accept the procedures that were described in the given documents. I am satisfied with the answers that I have provided, and I wish to take part in this study of my own accord. I also have a copy of this questionnaire.

[ ] I stand by the answers given in this questionnaire.

Name of the respondent: /______________________/ /_______________________/ /_______________________/
(First name) (Last name) (Place) (City)

Signature of the respondent __________________________________________________ Date _______________________

Signature of the surveyor __________________________________________________ Date _______________________

Please contact us if you have any questions or require any information (regarding your name and address), or if you have any other questions or need any advice.

If you feel you have been treated unfairly, or if you wish to ask any questions regarding your rights as a research subject, you may contact us. You may contact us at this number:Shobhini Mukherjee, JPAL South Asia, New Delhi 09871071555

**A0: Consent Form** Questionnaire code: |__|__| |__|__| |__|__| |__|__|__|

हम एक शोध अध्ययन संस्था से हैं। अब्दुल लतीफ जमील पोवर्टी एक्शन लैब साउथ एशिया (जे–पाल साउथ एशिया) का संचालन केन्द्र चेन्नई में है। हम आपसे टीबी/क्षय रोग से जुड़े आपके इलाज़ के बारे में कुछ प्रश्न पूछेंगे और आपके परिवार की कुछ निजी जानाकारियां प्राप्त करेंगे। इस सर्वे में हिस्सा लेने से पहले सोच सकते हैं कि आप इस सर्वे में हिस्सा लेना चाहते हैं या नहीं।

यह साक्षात्कार स्वैच्छिक है। अगर आप इस सर्वे में हिस्सा लेने के लिये राज़ी हैं तो भी आप किसी भी सवाल का जवाब देने से मना कर सकते हैं या किसी भी समय आप इस साक्षात्कार में हिस्सा लेने से मना कर सकते हैं।

अगर आप हिस्सा लेने के लिये तैयार हैं तो हम आपसे ऐसा ही दूसरा सर्वे 6 महीने बाद करेंगे। अगर आप चाहें तो उस समय भी इस सर्वे में हिस्सा लेने से मना कर सकते हैं।

जो भी जानकारी आप हमें देगें वह गुप्त रखी जाएगी। हम आपका नाम और जवाब किसी भी अन्य स्वाथ्य कर्मी को नहीं बतायेंगे। आपके जवाब का उपयोग सिर्फ शोध अध्ययन के लिये होगा और आपकी व्यावसायिक स्थिति पर इसका कोई प्रभाव नहीं पड़ेगा। इस सर्वे में आपके हिस्सा लेने के बारे में पुलिस, कोर्ट, सरकार या किसी और को कोई जानकारी नहीं दी जायेगी। हमारा यह शोध कुल तीन साल में पूरा होगा। आज के बाद 3 साल के लिए ये सारी प्रश्नावली हमारे जे–पाल कार्यालयए दिल्ली की अलमारी में रखी जायेंगी और उसके बाद सभी प्रश्नावलियों को नष्ट कर दिया जायेगा।

क्या आपको इस सर्वे की शर्ते मंजूर हैं? और क्या आप इस अध्ययन में अपनी मर्ज़ी से भाग लेना चाहते हैं?

[ ] दिये गये दस्तावेज में वर्णित प्रक्रिया मुझे मंजूर है। मेरे प्रश्नों के जो जवाब दिये गये उससे मैं संतुष्ट हूँ और अपनी मर्ज़ी से इस अध्ययन में हिस्सा लेना चाहताध्चाहती हूँ और मेरे पास इस प्रश्नावली की एक प्रति है।

[ ], मैं इस प्रश्नावली के जवाबों से सहमत हूँ।

जवाब देने वाले का नाम ध.......................................ध ध.......................................ध ध .....................................

(पहला नाम) (आख़िरी नाम) (स्थान ;शहर)

जवाब देने वाले के हस्ताक्षर .......................................ध दिनांक ..........................................

सर्वेक्षणकर्ता के हस्ताक्षर .......................................ध दिनांक ..........................................

आप कोई भी प्रश्न या जानकारी (आपके नाम और पते की जानकारी) व कोई प्रश्न व सलाह के लिए संपर्क करें।

यदि आपको लगता है कि आपके साथ सही बर्ताव नहीं किया गया, या आप एक शोध उत्तरदाता के रूप में अपने अधिकार से संबंधित कोई प्रश्न पूछना चाहते हैं तो आप हमें संपर्क कर सकते हैं। आप हमें इस नंबर पर संपर्क कर सकते हैं :– शोभिनी मुख़र्जी जेपाल दक्षिण एशिया नई दिल्ली . 0987107155

| अ: व्यक्तिगत विवरण<br>**A: Personal Information** | प्रश्नावली कोड/<br>Questionnaire code: \|__\|__\| \|__\|__\| \|__\|__\| \|__\|__\|__\| | | |
|---|---|---|---|
| *साक्षात्कारकर्ता . पहले मैं आपसे कुछ व्यक्तिगत और आपके परिवार सम्बन्धी प्रश्न पूछना चाहूँगा*<br>***INTERVIEWER: First, I would like to ask you some personal information as well as information about your family.*** | | | |
| A.0 | **सर्वेक्षण के प्रारंभ होने का समय**<br>*STARTING TIME OF THE SURVEY* | **घंटे** Hour [ ][ ]   **मिनट** Minutes [ ][ ] | |
| A.2 | **लिंग**<br>Gender<br><br>***साक्षात्कारकर्ता:उत्तरदाता के लिंग पर घेरा बनाएं***<br>*INTERVIEWER: Circle the respondent's gender* | **पुरुष**<br>Male 1<br><br>**स्त्री**<br>Female 2 | |
| A.3 | **आपकी जन्म तिथि क्या है?**<br>What is your birth date? | **जन्मतिथि** Birthday   दिन day [ ][ ]   **माह** month [ ][ ]   **वर्ष** year [ ][ ]   1<br><br>**जवाब नहीं देना**<br>Will not answer -888<br><br>**नहीं पता**<br>Does not know -999 | |
| A.4 | **आपकी उम्र क्या है?**<br>What is your age? | **आयु**<br>Age [ ][ ] 1<br><br>**जवाब नहीं दिया**<br>Will not answer -888<br><br>**नहीं पता**<br>Does not know -999 | |
| A.5 | **आपकी जाति क्या है?**<br>What is your caste?<br><br>**साक्षातकर्ता: जाति कोड सूची का उल्लेख करें**<br><br>*INTERVIEWER: REFER TO CASTE CODE LIST* | **Code/(कोड)** [ ][ ][ ][ ] 1<br><br>________________<br>**( स्पष्ट कीजिए)**<br>Other (Specify) -777<br><br>**जवाब नहीं देना**<br>Will not answer -888<br><br>**नहीं पता**<br>Does not know -999 | |

| | | | |
|---|---|---|---|
| A.6 | **आपकी जाति वर्ग क्या है?**<br>What is your caste category?<br><br>*साक्षात्कारकर्ता:उत्तर को पढ़ें*<br>*INTERVIEWER: READ THE ANSWERS* | **सामान्य**<br>General | 1 |
| | | **अन्य पिछड़ी जाति**<br>Other backward classes | 2 |
| | | **अनुसूचित जाति**<br>Scheduled caste | 3 |
| | | **अनुसूचित जनजाति**<br>Scheduled tribe | 4 |
| | | **अल्पसंख्यक (मुस्लिम, इसाई)**<br>Minority (Muslim, Christian) | 5 |
| | | **अन्य ( स्पष्ट कीजिए)**<br>Other (Specify) | -777 |
| | | **जवाब नहीं देना**<br>Will not answer | -888 |
| | | **नहीं पता**<br>Does not know | -999 |
| A.7 | **आपका धर्म क्या है?**<br>What is your religion? | **हिंदू**<br>Hindu | 1 |
| | | **मुस्लिम**<br>Muslim | 2 |
| | | **सिक्ख**<br>Sikh | 3 |
| | | **ईसाई**<br>Christian | 4 |
| | | **जैन**<br>Jain | 5 |
| | | **कोई अन्य (विवरण दे)**<br>Other (Specify) | -777 |
| | | **जवाब नहीं देना**<br>Will not answer | -888 |
| A.8 | **क्या आप पढ़ और लिख सकते है?**<br>Can you read and write? | **हाँ**<br>Yes | 1 |
| | | **नहीं**<br>No | 2 |
| | | **पढ़ सकते है लेकिन लिख नहीं सकते**<br>Can read but not write | 3 |
| | | **कोई अन्य (विवरण दे)**<br>Other (Specify) | -777 |
| | | **जवाब नहीं देना**<br>Will not answer | -888 |
| A.9 | **क्या आप वर्तमान में किसी स्कूल(औपचारिक या अनौपचारिक) में पढ़ रहे ?**<br>Are you currently attending school (formal or non-formal)? | **हाँ**<br>Yes | 1 |
| | | **नहीं**<br>No | 2 |
| | | **जवाब नहीं देना**<br>Will not answer | -888 |

| | | | | |
|---|---|---|---|---|
| A.10 | **आपने सफलतापूर्वक कहा तक पढाई की है?**<br>What is the highest level of education you have successfully completed? | **बालवाड़ी**<br>Balwadi | 1 | |
| | | **आंगनवाडी**<br>Anganwadi | 2 | |
| | | **एल.के.जी / नर्सरी**<br>LKG/Nursery | 3 | |
| | | **यु.के.जी / प्रेप**<br>UKG/Prep | 4 | |
| | | **पहली कक्षा भी पूरी नहीं की**<br>Did not complete Class 1 | 5 | |
| | | **कक्षा १**<br>Class 1 | 6 | |
| | | **कक्षा २**<br>Class 2 | 7 | |
| | | **कक्षा ३**<br>Class 3 | 8 | |
| | | **कक्षा ४**<br>Class 4 | 9 | |
| | | **कक्षा ५**<br>Class 5 | 10 | |
| | | **कक्षा ६**<br>Class 6 | 11 | |
| | | **कक्षा ७**<br>Class 7 | 12 | |
| | | **कक्षा ८**<br>Class 8 | 13 | |
| | | **कक्षा ९**<br>Class 9 | 14 | |
| | | **कक्षा १०**<br>Class 10 | 15 | |
| | | **कक्षा १०**<br>Class 11 | 16 | |
| | | **कक्षा १२**<br>Class 12 | 17 | |
| | | **स्नात्तक और अधिक**<br>Graduate and above | 18 | |
| | | **अन्य डिप्लोमा**<br>Other diploma | 19 | |
| | | **अनौपचारिक/प्रौढ़ शिक्षा(डिग्री के प्रकार का विवरण दे)**<br>Non-formal / Adult education (specify degree type) | 20 | |
| | | **कभी स्कूल नहीं गए**<br>Never attended school | 21 | Skip to **A.12** |
| | | **जवाब नहीं देना**<br>Will not answer | -888 | Skip to **A.12** |
| | | **पता नहीं**<br>Does not know | -999 | Skip to **A.12** |

| | | | |
|---|---|---|---|
| A.11 | **आपने स्कूल/विश्वविद्यालय कब जाना छोड़ दिया?**<br>When did you stop going to school /college /university? | **वर्तमान में पढ़ाई कर रहे है**<br>Currently studying | 1 |
| | | **माह** month ☐☐ **वष** year ☐☐☐☐ | 2 |
| | | **जवाब नहीं देना**<br>Will not answer | -888 |
| | | **पता नहीं**<br>Does not know | -999 |
| A.12 | **बचपन में आप घर मे कौन सी भाषाएँ बोलते थे?**<br>What languages did you speak at home as a child?<br><br>**साक्षातकर्ता: भाषा कोड सूची का उल्लेख करें**<br><br>*INTERVIEWER: REFER TO LANGUAGE CODE LIST*<br><br>**(उन सब पर घेरा बनाये जो लागू होते है)**<br>CIRCLE ALL THAT APPLY | **भाषा १**<br>Language 1 ☐☐☐ | 1 |
| | | **भाषा २**<br>Language 2 ☐☐☐ | 2 |
| | | **भाषा ३**<br>Language 3 ☐☐☐ | 3 |
| | | **भाषा ४**<br>Language 4 ☐☐☐ | 4 |
| | | **अन्य (स्पष्ट कीजिए)** ____<br>Other (Specify): | -777 |
| | | **जवाब नही देना**<br>Will not answer | -888 |
| A.13 | **क्या आप अन्य भाषाएँ सहजता से बोल लेते है?**<br>Do you speak other languages fluently?<br><br>**साक्षातकर्ता: भाषा कोड सूची का उल्लेख करें**<br>*INTERVIEWER: REFER TO LANGUAGE CODE LIST*<br>**(उन सब पर घेरा बनाये जो लागू होते है)**<br>CIRCLE ALL THAT APPLY | **भाषा १**<br>Language 1 ☐☐☐ | 1 |
| | | **भाषा २**<br>Language 2 ☐☐☐ | 2 |
| | | **भाषा ३**<br>Language 3 ☐☐☐ | 3 |
| | | **भाषा ४**<br>Language 4 ☐☐☐ | 4 |
| | | **भाषा ५**<br>Language 5 ☐☐☐ | 5 |
| | | **भाषा ६**<br>Language 6 ☐☐☐ | 6 |
| | | **कोई नहीं**<br>None | 7 |
| | | **अन्य (स्पष्ट कीजिए )** ____<br>Other (Specify): | -777 |
| | | **जवाब नहीं देना**<br>Will not answer | -888 |

| | | | | |
|---|---|---|---|---|
| A.14 | **क्या आप विवाहित है?**<br>Are you married? | **विवाहित**<br>Married | 1 | |
| | | **अलग रह रहे या तलाकशुदा**<br>Separated or divorced | 2 | |
| | | **विधवा/विधुर**<br>Widow/widower | 3 | |
| | | **अविवाहित**<br>Never married | 4 | Skip to **A.17** |
| | | **जवाब नहीं देना**<br>Will not answer | 5 | Skip to **A.17** |
| A.15 | **आपने किस वर्ष में विवाह किया?**<br>In what year did you marry? | **विवाह का वर्ष**<br>Year of marriage ☐☐☐☐ | 1 | |
| | | **जवाब नहीं देना**<br>Will not answer | -888 | |
| | | **नहीं पता**<br>Does not know | -999 | |
| A.16 | **आप कितने साल के थे, जब आपकी शादी हूई?**<br>How old were you when you married? | **आयु**<br>Age ☐☐ | 1 | |
| | | **जवाब नहीं देना**<br>Will not answer | -888 | |
| | | **नहीं पता**<br>Does not know | -999 | |
| A.17 | **क्या आप अकेले रहते है?**<br>Do you live alone? | **हाँ**<br>Yes | 1 | Skip to **A.20** |
| | | **नहीं**<br>No | 2 | |
| | | **जवाब नहीं देना**<br>Will not answer | -888 | Skip to **A.20** |
| A.18 | **आप किसके साथ रहते है?**<br>With whom do you live? | **पति/पत्नी**<br>Husband / wife | 1 | |
| | | **संतान**<br>Children | 2 | |
| | ***साक्षातकर्ता:उन सब पर घेरा लगाये जो लागू होते है.***<br>*INTERVIEWER: Circle all that apply* | **सौतेले बेटा या बेटी/बहू /दामाद**<br>Other son or daughter: in law/step | 3 | |
| | | **माता-पिता(पति/पत्नी के माता-पित** सम्मिलित)<br>Parents (including in laws) | 4 | |
| | | **भाई-बहन(पति/पत्नी के भाई बहन** सम्मिलित<br>Siblings (including in laws) | 5 | |
| | | **भतीजा/भतीजी**<br>Nephew/Niece | 6 | |

| | | | |
|---|---|---|---|
| | | **दादा-दादी/नाना-नानी**<br>Grandparents 7 | |
| | | **संतान की संतान**<br>Grandchildren 8 | |
| | | **चाचा/मौसी/बुआ/मामा**<br>Aunt/uncle 9 | |
| | | **चचेरे/मौसेरे भाई/बहन**<br>Cousins 10 | |
| | | **दोस्त**<br>Friends 11 | |
| | | **अन्य(स्पष्ट कीजिए )**<br>Other (specify) -777 | |
| | | **जवाब नहीं देना**<br>Will not answer -888 | |
| A.19 | **आपके साथ कितने लोग रहते है?(आपके अतिरिक्त)**<br>How many people live with you (excluding you)? | **व्यक्तियों की संख्यां**<br>Number of people [ ][ ] 1 | |
| | | **जवाब नहीं देना**<br>Will not answer -888 | |
| | | **नहीं पता**<br>Does not know -999 | |
| A.20 | **आप इस मोहल्ले में कब से रह रहे हैं?**<br>For how long have you lived in this neighborhood? | **हमेशा**<br>Always 1 | Skip to A.23 |
| | | **१० वर्षो से अधिक**<br>For more than 10 years 2 | |
| | | **६ से १० वर्षो से**<br>From 6 to 10 years 3 | |
| | | **१ से ५ वर्षो से**<br>From 1 to 5 years 4 | |
| | | **१ वर्ष से भी कम**<br>Less than a year 5 | |
| | | **जवाब नहीं देना**<br>Will not answer -888 | |
| | | **नहीं पता**<br>Does not know -999 | |

| अ: व्यक्तिगत विवरण<br>**A: Personal Information** | | प्रश्नावली कोड/<br>Questionnaire code: \|__\|__\| \|__\|__\| \|__\|__\| \|__\|__\|__\| | |
|---|---|---|---|
| A.21 | **आप/आपका परिवार इस स्थान पर रहने के लिए क्यों आये/आया?**<br><br>Why did you / your family move to this location?<br><br>*साक्षातकर्ता:उन सब पर घेरा लगाये जो लागू होते है.*<br><br>*INTERVIEWER: Circle all that apply* | **विवाह उपरान्त अपने पति/पत्नीके साथ रहने के लिए./मेरे पति यहाँ काम करते है** 1<br>Join my spouse after marriage/ my husband is working here. | |
| | | **बेहतर नौकरी की तलाश में** 2<br>Find a better job | |
| | | **रोग के उपचार के लिए** 3<br>Treat my illness | |
| | | **परिवार के किसी सदस्य के रोग के उपचार के लि** 4<br>Treat the illness of a family member | |
| | | **रिश्तेदारों के नजदीक रहने के लिए** 5<br>Come closer to relatives/family | |
| | | **अन्य(स्पष्ट कीजिए )** -777<br>Other (specify) | |
| | | **जवाब नहीं देना** -888<br>Will not answer | |
| | | **नहीं पता** -999<br>Does not know | |
| A.22 | **यहाँ आने से पहले आप कहाँ रहते थे?**<br>Where did you live before moving here?<br><br>*साक्षात्कारकर्ता:राज्य,जिला,शहर और इलाके के बारे में पूछे*<br><br>*INTERVIEWER: ASK FOR THE STATE, DISTRICT, CITY AND AREA* | **राज्य का नाम**<br>State name: ____________ | |
| | | **जिले का नाम**<br>District name: ____________ | |
| | | **शहर का नाम**<br>City name: ____________ | |
| | | **इलाके का नाम**<br>Area name: ____________ | |
| | | **जवाब नहीं देना** -888<br>Will not answer | |
| | | **नहीं पता** -999<br>Does not know | |
| A.23 | **पिछले तीन सालों के दौरान क्या आप किसी अन्य स्थान पर एक महीने से अधिक समय के लिए रहने गए है?**<br><br>Over the last three years, have you moved to another location for a period of more than a month?<br>If yes, how many times?<br>**अगर हाँ, तो कितने बार?** | **नहीं** 1<br>No | Skip to **B.0** |
| | | **हाँ** 2<br>Yes<br>**बारंबारता**<br>Number of times \|__\|__\| | |
| | | **जवाब नहीं देना** -888<br>Will not answer | |
| | | **नहीं पता** -999<br>Does not know | |

| | | | |
|---|---|---|---|
| A.24 | एक सामान्य महीने में कोई भी कारणवर्श आप कितनी राते घर से दूर बिताते है (जैसे काम, बीमारी, पढाई के लिए)<br>In a usual month how many nights do you spend out of your main location for any reason (work, illness, study etc.) | \|__\|__\| राते /nights | 1 |
| | | जवाब नहीं देना<br>Will not answer | -888 |
| | | नहीं पता<br>Does not know | -999 |
| A.25 | पिछली बार जब आप किसी अन्य स्थान पर एक महीने से अधिक समय के लिए रहने गए थे तो कहा गए थे?<br><br>Last time you moved to an other location for a period of more than a month, where did you go? | गृहनगर<br>My hometown | 1 |
| | | अन्य गांव<br>Another village | 2 |
| | | अन्य शहर<br>Another city | 3 |
| | | कोई महानगर (मुंबई,दिल्ली..)<br>A metro (Delhi, Mumbai...) | 4 |
| | | उसी शहर में कोई अन्य जगह<br>Another area in the same city | 5 |
| | | जवाब नहीं देना<br>Will not answer | -888 |
| | | नहीं पता<br>Does not know | -999 |
| A.26 | पिछली बार जब आप किसी अन्य स्थान पर एक महीने से अधिक समय के लिए रहने गए थे तो उसके पीछे उद्देश्य क्या था?<br><br>Last time you moved to an other location for a period of more than a month, what was the purpose?<br><br>*साक्षातकर्ता:उन सब पर घेरा लगाये जो लागू होते हैं.*<br><br>*INTERVIEWER: Circle all that apply* | पारिवारिक समारोह(विवाह,अंतिम यात्रा..)<br>Family function (wedding, funeral...) | 1 |
| | | काम की तलाश में<br>Get some work | 2 |
| | | रोग के उपचार के लिए<br>Treat my illness | 3 |
| | | परिवार में किसी रोगी व्यक्ति से मिलना<br>Visit an ill family member | 4 |
| | | संपत्ति केरखरखाव के लिए(भूमि,घर..)<br>Take care of property (land, house) | 5 |
| | | सम्बन्धी की प्रसूति में सहायता<br>Assist a relative's delivery | 6 |
| | | मेरे बच्चे का जन्म/पत्नी के साथ जाना बच्चे के जन्म के लिए<br>Deliver my child/ Accompany my wife for her delivery | 7 |
| | | जवाब नहीं देना<br>Will not answer | -888 |
| | | नहीं पता<br>Does not know | -999 |
| | | अन्य (स्पष्ट कीजिए)<br>Other (specify) | -777 |

| | |
|---|---|
| **ब: वेतन उत्पादक कार्यक्रियाएँ**<br>**B: Income Generating Activities** | प्रश्नावली कोड/<br>Questionnaire code: \|__\|__\| \|__\|__\| \|__\|__\| \|__\|__\|__\| |

***सशत्कार्कर्ता अब मे आपसे आपके द्वारा किये गए वेतन रोज़गार के सम्बंधित काम या किया के बारे मे पूछूँगा***
***INTERVIEWER: Next, I would like to ask you about any work you do to generate income.***

| | | | |
|---|---|---|---|
| B.0 | **पिछले तीन महीने के दौरान,क्या आपने आमदानी के लिए कुछ कार्य किया था?**<br>Over the last three months, did you engage in any income generating activities? | **हाँ** Yes 1<br>**नहीं** No 2<br>**जवाब नहीं देना** Will not answer -888 | <br>Skip to **C.0**<br>Skip to **C.0** |
| B.1 | **पिछले तीन महीने के दौरान,आपने आमदानी के लिए किस किस प्रकार के कार्य किये थे?**<br>Over the last three months, how many different income generating activities did you engage in? | ***संख्या*** *RECORD NUMBER* \|__\|__\| 1<br>**जवाब नहीं देना** Will not answer -888<br>**पता नहीं** Does not know -999 | |

***साक्षात्कारकर्ता जाँच बिंदु:सभी क्रियाएँ के लिए B2 -B 9 दुबारा पूंछे***
***INTERVIEWER CHECKPOINT: Repeat B.2-B.9 for all jobs mentioned.***

| | | | |
|---|---|---|---|
| B.2 | **यहाँ किस प्रकार का कार्य है?**<br>What type of activity is this?<br>**साक्षात्कारकर्ता:पेशा कोड सूची की सहायता से सम्बंधित कोड लिखे.**<br>*INTERVIEWER: Refer to occupation codes above* | **व्यावसायिक कोड** Occupational Code \|__\|__\| 1<br>________ Other (specify) -777<br>**जवाब नहीं देना** Will not answer -888<br>Does not know -999 | |
| B.3 | **रोग के लक्षण दिखने से पहले,आप इस कार्य के लिए एक सप्ताह में कितने दिन काम पर जाते थे?**<br>Before the symptoms appeared, how many days per week did you work for this job?<br>**साक्षात्कारकर्ता: दिनों की संख्या लिखें**<br>*INTERVIEWER: Record days* | **दिन**/ days \|__\|__\| 1<br>**जवाब नही देना** Will not answer -888<br>**पता नहीं** Does not know -999 | |
| B.4 | **पिछले सात दिनों के दौरान ,आपने कितने दिन इस काम को किया था?**<br>Over the last seven days, how many days per week did you work for this job?<br>**साक्षात्कारकर्ता: दिनों की संख्या लिखें**<br>*INTERVIEWER: Record days* | **दिन**/ days \|__\|__\| 1<br>**जवाब नही देना** Will not answer -888<br>**पता नहीं** Does not know -999 | |

| | | | |
|---|---|---|---|
| B.5 | **रोग के लक्षण दिखने से पहले,आप इस कार्य के लिए एक दिन में कितने घंटे काम करते थे?**<br>Before the symptoms appeared, how many hours per day did you work for this job?<br>**साक्षात्कारकर्ता: घंटों की संख्या लिखें**<br>*INTERVIEWER: Record hours* | **घंटे**/ hours [ ][ ] 1<br>**जवाब नही देना** Will not answer -888<br>**पता नहीं** Does not know -999 | |
| B.6 | **पिछले सात दिनों के दौरान ,आपने इस नौकरी के लिए एक दिन में कितने घंटे काम किया?**<br>Over the last seven days, how many hours per day did you work for this job?<br>**साक्षात्कारकर्ता: घंटों की संख्या लिखें**<br>*INTERVIEWER: Record hours* | **घंटे**/ hours [ ][ ] 1<br>**जवाब नही देना** Will not answer -888<br>**पता नहीं** Does not know -999 | |
| B.7 | **रोग के लक्षण दिखने से पहले,आपको इस कार्य से कितनी आमदानी होती थी?**<br>Before the symptoms appeared, how much money / equivalence of money did you earn from this job?<br>**साक्षात्कारकर्ता: आय और सम्बंधित अवधि लिखें**<br>*INTERVIEWER: Record amount AND time period* | **रू.**/Rs. [ ][ ][ ][ ]<br>**प्रति दिन** Per day 1<br>**प्रति सप्ताह** Per week 2<br>**प्रति माह** Per month 3<br>**जवाब नही देना** Will not answer -888<br>**पता नहीं** Does not know -999 | |
| B.8 | **पिछले सात दिनों के दौरान ,आपको इस कार्य से कितनी आमदानी होती थी?**<br>Over the past seven days, how much money / equivalence of money did you earn from this job?<br>**साक्षात्कारकर्ता: आय और सम्बंधित अवधि लिखें**<br>*INTERVIEWER: Record amount AND time period* | **रू.**/Rs. [ ][ ][ ][ ]<br>**प्रति दिन** Per day 1<br>**प्रति सप्ताह** Per week 2<br>**प्रति माह** Per month 3<br>**जवाब नहीं देना** Will not answer -888<br>**पता नहीं** Does not know -999 | |

| | | | | |
|---|---|---|---|---|
| B.9 | **जिस दिन से रोग के लक्षण दिखे हैं ,उस समय से इस कार्य से आपकी आय क्यों बढ़ी/घटी है?**<br>Why did your income from this activity increase/decrease since the symptoms appeared?<br><br>Circle all that apply<br>**जो लागू हो उन सब पर घेरा बनाये** | **अगर आय घटी है:**<br>***In case it decreased:*** | | |
| | | **मैं काम करने के लिए बहुत कमज़ोर/थक जाता था.**<br>I'm too weak/tired to work | 1 | |
| | | **मुझे कम काम करने के लिए कहा गया**<br>I was told to work less | 2 | |
| | | **रोग का उपचार मेरे काम करने के समय को कम कर देता है**<br>Taking my treatment reduces time for work | 3 | |
| | | **मेरा काम मौसमी / अनियमित है**<br>My work is seasonal/irregular | 4 | |
| | | **अन्य(विवरण दे):**<br>Other (Specify): | -777 | |
| | | **अगर आय बढ़ी है:**<br>***In case it increased:*** | | |
| | | **आय के अन्य साधनों में आई कमी को पूरा करने के लिए**<br>To compensate for a loss in other jobs | 6 | |
| | | **दवाइयां खरीदने के लिए**<br>To buy medicine | 7 | |
| | | **जब तक मैं सक्षम हूँ तब तक और धन कमाने की इच्छा से**<br>To make more money while I am still able to work | 8 | |
| | | **मेरी आमदनी स्थिर रही है**<br>My income remained stable | 9 | |
| | | **अन्य(विवरण दे)**<br>Other (Specify): | -777 | |
| | | जवाब नहीं देना<br>Will not answer | -888 | |
| | | **पता नहीं**<br>Does not know | -999 | |

## ब: वेतन उत्पादक कार्यक्रियाएँ
## B: Income Generating Activities (Continued)

Questionnaire code: |__|__| |__|__| |__|__| |__|__|__|

| | क्रिया १ Activity 1 | क्रिया २ Activity 2 | क्रिया ३ Activity 3 | क्रिया ४ Activity 4 |
|---|---|---|---|---|
| B.2 | 1 | 1 | 1 | 1 |
| | -777 | -777 | -777 | -777 |
| | -888 | -888 | -888 | -888 |
| | -999 | -999 | -999 | -999 |
| B.3 | दिन/days 1 | दिन/days 1 | दिन/days 1 | दिन/days 1 |
| | -888 | -888 | -888 | -888 |
| | -999 | -999 | -999 | -999 |
| B.4 | दिन/days 1 | दिन/days 1 | दिन/days 1 | दिन/days 1 |
| | -888 | -888 | -888 | -888 |
| | -999 | -999 | -999 | -999 |

 Questionnaire code: |__|__| |__|__| |__|__| |__|__|__|

| | | | | |
|---|---|---|---|---|
| B.5 | घंटे/hours [ ][ ] 1 | घंटे/hours [ ][ ] 1 | घंटे/hours [ ][ ] 1 | घंटे/hours [ ][ ] 1 |
| | -888 | -888 | -888 | -888 |
| | -999 | -999 | -999 | -999 |
| B.6 | घंटे/hours [ ][ ] 1 | घंटे/hours [ ][ ] 1 | घंटे/hours [ ][ ] 1 | घंटे/hours [ ][ ] 1 |
| | -888 | -888 | -888 | -888 |
| | -999 | -999 | -999 | -999 |
| B.7 | रू./Rs. [ ][ ][ ][ ] | रू./Rs. [ ][ ][ ][ ] | रू./Rs. [ ][ ][ ][ ] | रू./Rs. [ ][ ][ ][ ] |
| | 1 | 1 | 1 | 1 |
| | 2 | 2 | 2 | 2 |
| | 3 | 3 | 3 | 3 |
| | -888 | -888 | -888 | -888 |
| | -999 | -999 | -999 | -999 |
| B.8 | रू./Rs. [ ][ ][ ][ ] | रू./Rs. [ ][ ][ ][ ] | रू./Rs. [ ][ ][ ][ ] | रू./Rs. [ ][ ][ ][ ] |
| | 1 | 1 | 1 | 1 |
| | 2 | 2 | 2 | 2 |
| | 3 | 3 | 3 | 3 |
| | -888 | -888 | -888 | -888 |
| | -999 | -999 | -999 | -999 |

ब: वेतन उत्पादक कार्यक्रियाएँ

**B: Income Generating Activities (Continued)** Questionnaire code: |__|__| |__|__| |__|__| |__|__|__|

| | | | | |
|---|---|---|---|---|
| B.9 | | | | |
| | 1 | 1 | 1 | 1 |
| | 2 | 2 | 2 | 2 |
| | 3 | 3 | 3 | 3 |
| | 4 | 4 | 4 | 4 |
| | -777 | -777 | -777 | -777 |
| | | | | |
| | 6 | 6 | 6 | 6 |
| | 7 | 7 | 7 | 7 |
| | 8 | 8 | 8 | 8 |
| | 9 | 9 | 9 | 9 |
| | -777 | -777 | -777 | -777 |
| | -888 | -888 | -888 | -888 |
| | -999 | -999 | -999 | -999 |

साक्षात्कारकर्ता अब मैं आपसे आपकी नौकरी सम्बन्धी अपेक्षा के बारे में कुछ प्रश्न पूछूगा
*INTERVIEWER: Now I am going to ask a few questions about your work aspirations.*

| | | | | |
|---|---|---|---|---|
| C.0 | **साक्षात्कार जांच बिंदु:क्या रोगी वर्तमान समय में कोई कार्य कर रहा है?**<br><br>INTERVIEW CHECKPOINT: Is the patient currently working? (Includes self employment and regular wage) | **हाँ**<br>Yes | 1 | Skip to **D.1** |
| | | **नहीं**<br>No | 2 | |
| | | **जवाब नहीं देना**<br>Will not answer | -888 | |
| C.1 | **क्या आप नौकरी की तलाश में है?**<br>Are you looking for a job? | **हाँ**<br>Yes | 1 | |
| | | **नहीं**<br>No | 2 | |
| | | **जवाब नहीं देना**<br>Will not answer | -888 | |
| C.2 | **क्या आप बेरोजगार रहने के बजाय काम करना पसंद करेंगे ?**<br><br>Would you rather be working than not working? | **हाँ**<br>Yes | 1 | |
| | | **नहीं**<br>No | 2 | Skip to **C.4** |
| | | **जवाब नहीं देना**<br>Will not answer | -888 | Skip to **D.1** |
| C.3 | **आप काम क्यों करना चाहेंगे?**<br>Why would you rather be working?<br><br>**साक्षात्कारकर्ता: उन सभी पर घेरा बनाये जो लागू होते है**<br><br>INTERVIEWER: Circle all that apply | **धन की आवश्यकता**<br>Need the money | 1 | Skip to **D.1** |
| | | **काम करने पर जीवन मे आनद आता हैं.**<br>Life is more interesting working | 2 | Skip to **D.1** |
| | | **अधिक लोगों से मिलने के लिए**<br>Meet more people | 3 | Skip to **D.1** |
| | | **काम करना अच्छा लगता है**<br>Enjoy working | 4 | Skip to **D.1** |
| | | **अन्य(विवरण दे)** ______<br>Other (specify) | -777 | Skip to **D.1** |
| | | **जवाब नहीं देना**<br>Will not answer | -888 | Skip to **D.1** |
| | | **पता नहीं**<br>Does not know | -999 | |

| | | | | |
|---|---|---|---|---|
| C.4 | आप काम क्यों नहीं करना चाहते?<br>Why don't you want to work? | अत्यंत व्यस्त<br>Too busy to work | 1 | |
| | | काम करना अच्छा नहीं लगता<br>Do not like to work | 2 | |
| | साक्षात्कारकर्ता: उन सभी पर घेरा बनाये जो लागू<br>*INTERVIEWER: Circle all that apply* | काम करने के लिए बहुत अस्वस्थ महसूस करता हूँ<br>Feel too unwell to work | 3 | |
| | | परिवार को घर पर मेरी आवश्यकता होती है<br>Family needs me at home | 4 | |
| | | लोग मुझे नौकरी पर नहीं रखना चाहते<br>Generally people don’t like to hire me for work | 5 | |
| | | योग्यता नहीं है इसलिए काम नहीं कर सकता<br>Have no skills so cannot work | 6 | |
| | | मुझे आर्थिक रूप से काम करने की आवश्यकता नहीं है<br>I don't need (financially) to work | 7 | |
| | | अन्य(विवरण दे)<br>Other (specify) | -777 | |
| | | जवाब नहीं देना<br>Will not answer | -888 | |
| | | पता नहीं<br>Does not know | -999 | |

# द.पारिवारिक इतिहास

# D: Family History

प्रश्नावली कोड/
Questionnaire code: |__|__| |__|__| |__|__| |__|__|__|

साक्षात्कारकर्ता अब मैं आपसे आपके परिवार के इतिहास के बारे में कुछ प्रश्न पूछूंगा
INTERVIEWER: Now I am going to ask a few questions about your family history.

| | | | |
|---|---|---|---|
| D.1 | **जबसे आपका जन्म हुआ है,आपके घर/ परिवार में कितने लोगों को टी.बी. हो चुका है ?**<br>How many members of your family / household have had TB since you were born?<br>*घर मे वह सभी शामिल हे जो एक रसोइ से खतें है। परिवार मे मात और पीता के तरफ़ के रिशतेदार और शादी के बाद ससुराल के लोग शामिल है।*<br>*Household includes people living with the patient and eating from the same kitchen. Family includes maternal, paternal and in-laws after marriage* | **संख्या**<br>RECORD NUMBER [ ][ ] 1 | If 0 Skip to **E.0** |
| | | **जवाब नहीं देना**<br>Will not answer -888 | Skip to **E.0** |
| | | **पता नहीं**<br>Doesn't Know -999 | Skip to **E.0** |

साक्षात्कारकर्ता जांच बिंदु: प्रत्येक सदस्य से प्रश्न संख्या D.2-D.14 पूछे ;ग्रिड पर दर्ज करें.
INTERVIEWER CHECKPOINT: Ask questions D.2-D.14 for each member; Record on grid

| | | | |
|---|---|---|---|
| D.2 | **इस व्यक्ति का नाम क्या है?**<br>What is this person's name? | **साक्षात्कारकर्ता: इस व्यक्ती का नाम ना लिखे सिर्फ़ पूछने के लिये इस्तमाल करे।**<br>INTERVIEWER: DO NOT RECORD THIS PERSON'S NAME ON THIS SURVEY<br>Use the name as a reference only | |
| D.3 | **इस व्यक्ति का आपसे क्या सम्बन्ध है?**<br>What is this person's relation to you? | **पति/पत्नी**<br>Husband / wife 1 | |
| | | **बच्चे**<br>Children 2 | |
| | | **अन्य बहू/दामाद/सौतेली संतान**<br>Other son or daughter: in law/step 3 | |
| | | **माता-पिता/सास-ससूर**<br>Parents (including in laws) 4 | |
| | | **भाई-बहन(पति/पत्नी के भाई भाई बहन भी सम्मिलित)**<br>Siblings (including in laws) 5 | |
| | | **भतीजा/भतीजी**<br>Nephew/Niece 6 | |
| | | **दादा-दादी/नाना-नानी**<br>Grandparents 7 | |
| | | **संतान की संतान**<br>Grandchildren 8 | |
| | | **चाचा/बूआ/मामा आदि**<br>Aunt/uncle 9 | |
| | | **चचेरे/मौसेरे भाई/बहन**<br>Cousins 10 | |
| | | **अन्य(विवरण दे)**<br>Other (specify) -777 | |
| | | **जवाब नही देना**<br>Will not answer -888 | |
| | | **पता नहीं**<br>Does not know -999 | |

# द.पारिवारिक इतिहास
# D: Family History

Questionnaire code: |__|__| |__|__| |__|__| |__|__|__|

| | | | |
|---|---|---|---|
| D.4 | **क्या उन्होंने टी.बी.का उपचार कराया था ?**<br>Did he/she take a treatment against TB? | **हॉ**<br>Yes 1<br><br>**नहीं**<br>No 2<br><br>**जवाब नही देना**<br>Will not answer -888<br><br>**पता नहीं**<br>Does not know -999 | <br><br>Skip to **D.9** |
| D.5 | **उन्होंने कब टी.बी. का उपचार शुरू किया?**<br>When did he/she start taking a TB treatment? | **माह**/month [ ][ ] **वर्ष**/ year [ ][ ][ ][ ] 1<br><br>**जवाब नहीं देना**<br>Will not answer -888<br><br>**पता नहीं**<br>Does not know -999 | |
| D.6 | **उनका उपचार कहाँ से हुआ?**<br>From where did they receive their treatment? | **निजी डॉक्टर**<br>Private doctor 1<br><br>**सरकारी अस्पताल/डिस्पेंसरी**<br>Govt hospital/dispensary 2<br><br>**सरकारी डॉट्स केंद्र**<br>Govt DOTS center 3<br><br>**एन.जी.ओ.द्वारा संचालित डॉट्स केंद्र**<br>NGO-run DOTS center 4<br><br>**निजी खरीदी गई दवाइयों से**<br>Medicines via private purchase 5<br><br>**अन्य (विवरण दे)**<br>Other (specify)________ -777<br><br>**जवाब नहीं देना**<br>Will not answer -888<br><br>**पता नहीं**<br>Does not know -999 | |

| | | | |
|---|---|---|---|
| D.7 | उनका उपचार कितने समय चला?<br>How long did he/she take the treatment? | कुछ दिन<br>A few days 1 | |
| | | कुछ सप्ताह<br>A few weeks 2 | |
| | | कुछ माह<br>A few months 3 | |
| | | छः माह या अधिक<br>Six months or more 4 | |
| | | अन्य(विवरण दे)<br>Other (specify) -777 | |
| | | जवाब नहीं देना<br>Will not answer -888 | |
| | | पता नहीं<br>Does not know -999 | |
| D.8 | उनके उपचार का क्या परिणाम रहा?<br>What was the result of their treatment? | उपचार अभी भी चल रहा है<br>Treatment is still ongoing 1 | Skip to **D.13** |
| | | उपचार बीच में रुक गया था<br>Treatment was interrupted 2 | |
| | | व्यक्ति की उपचार के दौरान मृत्यु होगयी<br>Person died during the treatment 3 | Skip to **D.10** |
| | | उपचार के दौरान अस्पताल मे दाखिल किया ग<br>Person was admitted during the treatment 4 | |
| | | उपचार पूरा हो गया परन्तु रोगी की स्थिति में कोई सुधर नहीं हुआ<br>Treatment was completed without improvement 5 | |
| | | वो ठीक होगये<br>Person was cured 6 | |
| | | अन्य(विवरण दे)<br>Other (specify) -777 | |
| | | जवाब नहीं देना<br>Will not answer -888 | |
| | | पता नहीं<br>Does not know -999 | |
| D.9 | क्या वे अभी भी जीवित है?<br>Is the person still alive? | हाँ<br>Yes 1 | Skip to **D.13** |
| | | नहीं<br>No 2 | |
| | | जवाब नहीं देना<br>Will not answer -888 | |
| | | पता नहीं<br>Does not know -999 | |

# द.पारिवारिक इतिहास
# D: Family History

Questionnaire code: |__|__| |__|__| |__|__| |__|__|__|

| | | | |
|---|---|---|---|
| D.10 | उनकी मृत्यु की क्या वजह थी?<br>What was the cause of death?<br><br>Circle all that apply<br>जो लागू हो उन सब पर घेरा बनाये | टी.बी.<br>TB 1<br>वृद्धावस्था<br>Old age 2<br>प्रसूति के समय<br>During child birth 3<br>हृदय रोग / उच्च रक्तचाप<br>Heart disease / hypertension 4<br>मधुमेह<br>Diabetes 5<br>निमोनिया<br>Pneumonia 8<br>दुर्घटना<br>Accident 9<br>अन्य(विवरण दे) ____<br>Other (specify) -777<br>जवाब नहीं देना<br>Will not answer -888<br>पता नहीं<br>Does not know -999 | |
| D.11 | उनकी मृत्यु कब हुई?<br>When did this person die? | month माह [ ][ ] year साल [ ][ ] 1<br>जवाब नहीं देना<br>Will not answer -888<br>पता नहीं<br>Does not know -999 | |
| D.12 | जब मृत्यु हुई तब उनकी उम्र क्या थी?<br>At what age did this person die?<br><br>साक्षात्कारकर्ता:वर्ष में अगर >२वर्ष,महीनो में अगर <२ वर्ष,दिनों में अगर <२ महीने ,<br>INTERVIEWER: In years if >2 years; in months is less than 2 years; in days if less than 2 months | आयु<br>Age<br>वर्ष years [ ][ ] 1<br>माह months [ ][ ] 2<br>दिन days [ ][ ] 3<br>जवाब नहीं देना<br>Will not answer -888<br>पता नहीं<br>Does not know -999 | Skip to **E.0**<br><br>Skip to **E.0**<br>Skip to **E.0** |

| | | | |
|---|---|---|---|
| D.13 | **अब उनकी उम्र क्या है?**<br>What is their age now?<br><br>**साक्षात्कारकर्ता:वर्ष में अगर >२वर्ष,महीनो में अगर <२ वर्ष,दिनों में अगर <२ महीने ,**<br>INTERVIEWER: In years if >2 years; in months is less than 2 years; in days if less than 2 months | **आयु** Age — **वर्ष** years [ ][ ] 1<br>**माह** months [ ][ ] 2<br>**दिन** days [ ][ ] 3<br>जवाब नहीं देना Will not answer -888<br>पता नहीं Does not know -999 | |
| D.14 | **क्या वे आपके साथ घर में रहते है?**<br>Does he/she live in your household? | **हाँ** Yes 1<br>**नहो** No 2<br>**जवाब नहीं देना** Will not answer -888<br>**पता नहीं** Does not know -999 | |

# द.पारिवारिक इतिहास
# D: Family History

Questionnaire code: |__|__| |__|__| |__|__| |__|__|__|

| | | | |
|---|---|---|---|
| 1 | 1 | 1 | 1 |
| 2 | 2 | 2 | 2 |
| 3 | 3 | 3 | 3 |
| 4 | 4 | 4 | 4 |
| 5 | 5 | 5 | 5 |
| 6 | 6 | 6 | 6 |
| 7 | 7 | 7 | 7 |
| 8 | 8 | 8 | 8 |
| 9 | 9 | 9 | 9 |
| 10 | 10 | 10 | 10 |
| -777 | -777 | -777 | -777 |
| -888 | -888 | -888 | -888 |
| -999 | -999 | -999 | -999 |

# द.पारिवारिक इतिहास
# D: Family History

Questionnaire code: |__|__| |__|__| |__|__| |__|__|__|

| | | | |
|---|---|---|---|
| 1 | 1 | 1 | 1 |
| Skip to **D.9** 2 | Skip to **D.9** 2 | Skip to **D.9** 2 | Skip to **D.9** 2 |
| -888 | -888 | -888 | -888 |
| -999 | -999 | -999 | -999 |
| **माह/month** [ ][ ] **वर्ष/year** [ ][ ][ ][ ] 1 | **माह/month** [ ][ ] **वर्ष/ year** [ ][ ][ ][ ] 1 | **माह/month** [ ][ ] **वर्ष/year** [ ][ ][ ][ ] 1 | **माह/month** [ ][ ] **वर्ष/year** [ ][ ][ ][ ] 1 |
| -888 | -888 | -888 | -888 |
| -999 | -999 | -999 | -999 |
| 1 | 1 | 1 | 1 |
| 2 | 2 | 2 | 2 |
| 3 | 3 | 3 | 3 |
| 4 | 4 | 4 | 4 |
| 5 | 5 | 5 | 5 |
| -777 ________ | -777 ________ | -777 ________ | -777 ________ |
| -888 | -888 | -888 | -888 |
| -999 | -999 | -999 | -999 |

# द.पारिवारिक इतिहास
# D: Family History

Questionnaire code: |__|__| |__|__| |__|__| |__|__|__|

| | | | |
|---|---|---|---|
| 1 | 1 | 1 | 1 |
| 2 | 2 | 2 | 2 |
| 3 | 3 | 3 | 3 |
| 4 | 4 | 4 | 4 |
| ________ -777 | ________ -777 | ________ -777 | ________ -777 |
| -888 | -888 | -888 | -888 |
| -999 | -999 | -999 | -999 |
| Skip to **D.13** 1 | Skip to **D.13** 1 | Skip to **D.13** 1 | Skip to **D.13** 1 |
| 2 | 2 | 2 | 2 |
| Skip to **D.10** 3 | Skip to **D.10** 3 | Skip to **D.10** 3 | Skip to **D.10** 3 |
| 4 | 4 | 4 | 4 |
| 5 | 5 | 5 | 5 |
| 6 | 6 | 6 | 6 |
| ________ -777 | ________ -777 | ________ -777 | ________ -777 |
| -888 | -888 | -888 | -888 |
| -999 | -999 | -999 | -999 |
| Skip to **D.13** 1 | Skip to **D.13** 1 | Skip to **D.13** 1 | Skip to **D.13** 1 |
| 2 | 2 | 2 | 2 |
| -888 | -888 | -888 | -888 |
| -999 | -999 | -999 | -999 |

## द.पारिवारिक इतिहास
## D: Family History

Questionnaire code: |__|__| |__|__| |__|__| |__|__|__|

| | | | |
|---|---|---|---|
| 1 | 1 | 1 | 1 |
| 2 | 2 | 2 | 2 |
| 3 | 3 | 3 | 3 |
| 4 | 4 | 4 | 4 |
| 5 | 5 | 5 | 5 |
| 8 | 8 | 8 | 8 |
| 9 | 9 | 9 | 9 |
| ____________ -777 | ____________ -777 | ____________ -777 | ____________ -777 |
| -888 | -888 | -888 | -888 |
| -999 | -999 | -999 | -999 |
| month माह [ ][ ] year साल [ ][ ] 1 | month माह [ ][ ] year साल [ ][ ] 1 | month माह [ ][ ] year साल [ ][ ] 1 | month माह [ ][ ] year साल [ ][ ] 1 |
| -888 | -888 | -888 | -888 |
| -999 | -999 | -999 | -999 |
| आयु Age वर्ष years [ ][ ] 1<br>माह months [ ][ ] 2<br>दिन days [ ][ ] 3<br>Skip to E.0 | आयु Age वर्ष years [ ][ ] 1<br>माह months [ ][ ] 2<br>दिन days [ ][ ] 3<br>Skip to E.0 | आयु Age वर्ष years [ ][ ] 1<br>माह months [ ][ ] 2<br>दिन days [ ][ ] 3<br>Skip to E.0 | आयु Age वर्ष years [ ][ ] 1<br>माह months [ ][ ] 2<br>दिन days [ ][ ] 3<br>Skip to E.0 |
| Skip to E.0 -888 | Skip to E.0 -888 | Skip to E.0 -888 | Skip to E.0 -888 |
| Skip to E.0 -999 | Skip to E.0 -999 | Skip to E.0 -999 | Skip to E.0 -999 |

# द.पारिवारिक इतिहास
# D: Family History

Questionnaire code: |__|__| |__|__| |__|__| |__|__|__|

| | | | |
|---|---|---|---|
| **आयु** Age **वर्ष** years ☐☐ 1<br>**माह** months ☐☐ 2<br>**दिन** days ☐☐ 3 | **आयु** Age **वर्ष** years ☐☐ 1<br>**माह** months ☐☐ 2<br>**दिन** days ☐☐ 3 | **आयु** Age **वर्ष** years ☐☐ 1<br>**माह** months ☐☐ 2<br>**दिन** days ☐☐ 3 | **आयु** Age **वर्ष** years ☐☐ 1<br>**माह** months ☐☐ 2<br>**दिन** days ☐☐ 3 |
| -888 | -888 | -888 | -888 |
| -999 | -999 | -999 | -999 |
| 1 | 1 | 1 | 1 |
| 2 | 2 | 2 | 2 |
| -888 | -888 | -888 | -888 |
| -999 | -999 | -999 | -999 |

***साक्षात्कारकर्ता  अब मैं आपसे आपके उन सभी बच्चो जो अभी तक रही हैएसे सम्बंधित प्रश्न पूछूँगा***
***INTERVIEWER: Next, I am going to ask a few questions about any children you have had.***

| | | | |
|---|---|---|---|
| E.0 | **वर्तमान मे आपके कितने बच्चें है?**<br>How many living children do you have?<br>*सारे सर्वे के समय जीवित बच्चे जो मरीज़ को जन्मे हे और जिनहें कनूनी तरीके से मरीज़ ने गोद लिये को शामिल करे*<br>*Include all childen born to the patient and legally adopted who are living at the time of survey* | **संख्या लिखें** *RECORD NUMBER* [ ][ ] 1<br>**जवाब नहीं देना** Will not answer -888<br>**पता नहीं** Does not know -999 | |
| E.1 | **क्या आपके किसी बच्चे की मृत्यु हुई है?(गर्भपात,नवजात शिशु मृत्यु आदि )यदि हाँ,तो कितने?**<br>Have any of your children died (including abortion, still birth, neo-natal death)? If yes, how many? | **हाँ** Yes 1<br>**संतानों की संख्या** Number of children [ ][ ] | |
| | | **नहीं** No 2 | Skip to **E.3** |
| | | **जवाब नहीं देना** Will not answer -888 | Skip to **E.3** |
| | | **पता नहीं** Does not know -999 | Skip to **E.3** |
| E.2 | **बच्चे की मृत्यु का कारन क्या था?**<br>What was the cause of death?<br>**जो भी लागू होता है उस पर घेरा बनाये**<br>*CIRCLE ALL THAT APPLY* | **गर्भपात** Abortion — संख्या लिखें *Record number* [ ][ ] 1<br>**मृत शिशु का जन्म** Still birth — संख्या लिखें *Record number* [ ][ ] 2<br>**नवजात शिशु सम्बन्धी रोग** Neonatal causes — संख्या लिखें *Record number* [ ][ ] 3<br>**टी.बी.** TB — संख्या लिखें *Record number* [ ][ ] 4<br>**दुर्घटना** Accident — संख्या लिखें *Record number* [ ][ ] 5<br>**मलेरिया** Malaria — संख्या लिखें *Record number* [ ][ ] 6<br>**अन्य(विवरण दे )** Other (specify)____________ — संख्या लिखें *Record number* [ ][ ] -777<br>जवाब नहीं देना Will not answer -888<br>**पता नहीं** Does not know -999 | |

***साक्षात्कारकर्ता  अब मैं आपसे कुछ प्रश्न उन सभी १४ वर्ष से कम बच्चो के बारे मै पूछूँगा जो आपके साथ रहते हैए***
***INTERVIEWER: Next, I am going to ask a few questions about all the children less than the age of 14 years who are living with you.***

| | | | |
|---|---|---|---|
| E.3 | **आपके साथ आपके घर में कितने बच्चे रहते है?**<br>How many children live in your household?<br>*सर्वेक्षक: कृपया जवाब दिने वाले तो ध्यान दिलादें कि 14 वर्ष से कम उम्र के बच्चे जो उनके साथ रहते हे, के बारे मे जवाब देना है।*<br>*Interviewer: Please remind the respondent to give number of all children below theage of 14 that are living with them.* | **सख्या लिखे** *RECORD NUMBER* [ ][ ] 1 | If **0** Skip to **F.0** |
| | | **जवाब नही देना** Will not answer -888 | |
| | | पता नहीं Does not know -999 | Skip to F. 0 |

| | | | |
|---|---|---|---|
| E.4 | **कितने बच्चे आपके कमरे मे सोते है?**<br>How many children sleep in your room? | **सख्या लिखें**<br>*RECORD NUMBER* [ ][ ] 1<br><br>**जवाब नही देना**<br>Will not answer -888<br>**पता नहीं**<br>Does not know -999 | |
| E.5 | **कितने बच्चो को बी सी जी का टीका लगा है?**<br>How many children got vaccinated for BCG? | **सख्या लिखें**<br>*RECORD NUMBER* [ ][ ] 1<br><br>**जवाब नहा दना**<br>Will not answer -888<br><br>**पता नहीं**<br>Does not know -999 | If **0** Skip to **E.7**<br><br><br><br>Skip to **E.7** |
| E.6 | **उनको बी.सी.जी. के टीके कब लगे?**<br>When were they vaccinated?<br><br>**जो भी लागू होता है उस पर घेरा बनाये**<br>*CIRCLE ALL THAT APPLY* | **जन्म पर** संख्या लिखें<br>At birth *Record number* [ ][ ] 1<br>**बचपन के दौरान** सख्या लिखें<br>During their childhood *Record number* [ ][ ] 2<br><br>**जब से लक्षण दिखे** संख्या लिखें<br>Since I got symptoms *Record number* [ ][ ] 3<br>**अन्य समय पर** संख्या लिखें<br>Some other time *Record number* [ ][ ] 4<br><br>**जवाब नहीं देना**<br>Will not answer -888<br><br>**पता नहीं**<br>Does not know -999 | |
| E.7 | **जब से आपके टी.बी. के लक्षण सामने आये तब से कितने बच्चों की टी.बी की जांच हुई है?**<br>How many children got tested for TB since your symptoms started? | **सख्या लिखें**<br>*RECORD NUMBER* [ ][ ] 1<br><br>**जवाब नहीं देना**<br>Will not answer -888<br><br>**पता नही**<br>Does not know -999 | If **0** Skip to **E.9** |
| E.8 | **उनमें से कितने बच्चों की टी.बी. होने की पुष्टि हुई?**<br>How many children are TB positive? | **संख्या लिखें**<br>*RECORD NUMBER* [ ][ ] 1<br><br>**जवाब नहीं देना**<br>Will not answer -888<br><br>**पता नहीं**<br>Does not know -999 | |

| | | | |
|---|---|---|---|
| E.9 | **क्या कोई छः वर्ष से कम उम्र का बच्चा है जो आपके साथ रहता हो?**<br>Do you have children under 6 living with you? | **सख्या लिखे**<br>*RECORD NUMBER* [ ][ ] 1<br>**जवाब नही देना**<br>Will not answer -888<br>**पता नहीं**<br>Does not know -999 | If **0** Skip to **F.0** |
| E.10 | **क्या उनका टी.बी. निरोधक उपचार हुआ?**<br>Do they get preventive treatment for TB? | **हाँ,सबका**<br>Yes, all of them 1<br>**हाँ,उनमे से कुछ का**<br>Yes, some of them 2<br>**किसी का नही**<br>None of them 3<br>**जवाब नही देना**<br>Will not answer -888<br>**पता नहीं**<br>Does not know -999 | <br><br><br><br>Skip to **F.0** |
| E.11 | **उन्हें इसके उपचार के लिए कितनी बार गोलियाँ लेनी पड़ती**<br>How often do they have to take pills? | **सख्या प्रति सप्ताह**<br>*RECORD NUMBER OF TIMES PER WEEK* [ ][ ] 1<br>**जवाब नही देना**<br>Will not answer -888<br>**पता नहीं**<br>Does not know -999 | |

## फ.स्वस्थ्य
## F: Health

प्रश्नावली कोड/Questionnaire code: | | || | || | || | | |

**साक्षात्कारकर्ता:अभी तक हमारे सभी प्रश्नों का उत्तर देने के लिए धन्यवाद.अब हम आपके स्वस्थ्य से जुड़े कुछ सटीक प्रश्न पूछेंगे.**

INTERVIEWER: Thanks for answering all of our questions so far. Now we are going to ask specific questions related to your health.

| | | | |
|---|---|---|---|
| F.0 | **आप इन दिनों अपने स्वस्थ्य को किस प्रकार वर्गीकृत करेंगे?अगर साथ में बनी सीढ़ीनुमा आकृति का सबसे उपरी हिस्सा बहुत अच्छे स्वास्थ्य का और सबसे नीचे का हिस्सा बेहद खराब स्वास्थ्य,को दर्शाता है .आप अपने स्वास्थ्य को इस आकृति में किधर दर्शाएंगे.**<br>How would you classify your health these days? If the top rung of this ladder represents very good health and the bottom rung represents very bad health, where would you place yourself?<br><br>**साक्षात्कारकर्ता:उत्तरदाता को सीढ़ीनुमा आकृति दिखाएँ और उस अंक पर घेरा बना दे जो वो चुने .**<br>INTERVIEWER: SHOW THE RESPONDENT THE PICTURE OF THE LADDER AND CIRCLE THE NUMBER THAT CORRESPONDS TO THE NUMBER THEY CHOOSE. | **सबसे अच्छा स्वास्थ्य**<br>Top rung | 10 |
| | | | 9 |
| | | | 8 |
| | | | 7 |
| | | | 6 |
| | | | 5 |
| | | | 4 |
| | | | 3 |
| | | | 2 |
| | | **बेहद खराब स्वास्थ्य**<br>Bottom rung | 1 |
| | | **जवाब नहीं देना**<br>Will not answer | -888 |
| | | **पता नहीं**<br>Does not know | -999 |

**क्या आपको निम्नलिखित गतिविधियां करने में कठिनाई होती है,उदाहरण के लिए...**
Have you experienced any difficulty in performing any of the following activities, for example…

| | | | **कोई कठिनाई नहीं**<br>No difficulty | **कठिन परन्तु कर लेता/लेती हूँ**<br>Difficult, but could do without help | **सहायता से कर सकता हूँ**<br>Can do with help | **असक्षम हूँ**<br>Not able to do | **नहीं बताना**<br>Will not say | **पता नहीं**<br>Does not know |
|---|---|---|---|---|---|---|---|---|
| F.1 | **कुएँ से पानी खीचना**<br>Drawing water from a well | | 1 | 2 | 3 | 4 | -888 | -999 |
| F.2 | **रोजाना का घरेलु काम करने में जैसे खाना बनाना, सफाई...**<br>Routine housework such as cleaning or cooking | | 1 | 2 | 3 | 4 | -888 | -999 |
| F.3 | **रोजाना दिन की गतिविधियाँ (कपड़े पहनना,नहाना...)**<br>Routine daily activities (dressing, bathing…) | | 1 | 2 | 3 | 4 | -888 | -999 |
| F.4 | **अपने आम तोर के काम करने में**<br>Maintaining your usual work schedule | 5<br>**लागू नही**<br>Not Applicable | 1 | 2 | 3 | 4 | -888 | -999 |

| | | | |
|---|---|---|---|
| F.5 | **क्या आप निम्न में से किसी प्रकार की विकलांगता/ बीमारियाँ से पीड़ित है?**<br>Do you have any of the following permanent disabilities/conditions?<br><br>किवल लम्बे समय से चल रही गंभीर बिमारी या विकलांता शामिल करे जो मरीज़ को आम काम करने मे बाधा डालती है।<br>Includes serious long term illnesses and disabilities that hamper patient's normal functioning.<br><br>**साक्षात्कारकर्ता:कृपया पढकर सुनायें**<br>*INTERVIEWER: Please prompt*<br>**जो भी लागू होता है उस पर घेरा बनाये**<br>*CIRCLE ALL THAT APPLY* | **पूर्ण/आंगिक लकुवा**<br>Full/partial paralysis | 1 |
| | | **किसी अंग का न होना/जख्मी होना**<br>Missing/injured limb | 2 |
| | | **याददाश्त का कमज़ोर होना/मानसिक रोग**<br>Memory loss / mental illness | 3 |
| | | **सुनने मे कठिनाई/बहरापन**<br>Hearing problems / deafness | 4 |
| | | **दृष्टि सम्बन्धी समस्या /अंधापन**<br>Vision problems / blindness | 5 |

| | | | |
|---|---|---|---|
| | | मधुमेह<br>Diabetes 6 | |
| | | उच्च रक्तचाप<br>Hypertension 7 | |
| | | दमा<br>Asthma 8 | |
| | | हृदय रोग<br>Heart disease 9 | |
| | | एच.आई.वी./एड्स<br>HIV/AIDS 10 | |
| | | कोई भी नहीं<br>None 11 | Skip to F.7 |
| | | अन्य(विवरण दे )<br>Other (specify) -777 | |
| | | जवाब नहीं<br>Will not answer -888 | Skip to F.7 |
| | | पता नहीं<br>Does not know -999 | Skip to F.7 |
| F.6 | क्या विकलांगता या जिन शारीरिक कठिनाइयों का आप सामना करते है ,आपको काम करने से रोकते है?<br><br>Do the disabilities /condition that you experience prevent you from working? | हां,महत्वपूर्ण रूप से<br>Yes, significantly 1 | |
| | | हां,कुछ हद तक<br>Yes, moderately 2 | |
| | | हां,बहुत कम<br>Yes, very little 3 | |
| | | नहीं<br>No 4 | |
| | | जवाब नहीं<br>Will not answer -888 | |
| F.7 | क्या आपको डॉट्स केंद्र से मिली गोलियाँ लेने से ३० दिन पहले खांसी हुई ?<br>Have you experienced a cough in the 30 days before you started taking pills at the DOTS center? | हाँ<br>Yes 1 | |
| | | नहीं<br>No 2 | Skip to F.9 |
| | | जवाब नहीं देना<br>Will not answer -888 | Skip to F.9 |
| | | पता नहीं<br>Does not know -999 | Skip to F.9 |

| | | | |
|---|---|---|---|
| F.8 | खांसी कैसी थी?<br>Was the cough…<br><br>जो भी लागू होता है उस पर घेरा बनाये<br>*CIRCLE ALL THAT APPLY* | अत्यत ददेनाक थी<br>Very painful 1 | |
| | | दर्दनाक थी<br>Painful 2 | |
| | | खांसी के साथ खून/बलगम आया<br>Accompanied with phlegm/blood 3 | |
| | | ऊपर से कोई भी नहीं<br>None of the above 4 | |
| | | जवाब नही देना<br>Will not answer -888 | |
| | | पता नही<br>Does not know -999 | |
| F.9 | जाब से आपने डॉट्स केंद्र की दवाइयां खानी शुरू की है क्या तब से कभी खांसी हई है?<br>Have you experienced a cough SINCE you started taking pills at the DOTS center? | हॉं<br>Yes 1 | |
| | | नहीं<br>No 2 | Skip to F.11 |
| | | जवाब नही देना<br>Will not answer -888 | Skip to F.11 |
| | | पता नही<br>Does not know -999 | Skip to F.11 |
| F.10 | खांसी कैसी थी?<br>Was the cough…<br><br>जो भी लागू होता है उस पर घेरा बनाये<br>*CIRCLE ALL THAT APPLY* | अत्यत ददेनाक थी<br>Very painful 1 | |
| | | दर्दनाक थी<br>Painful 2 | |
| | | के साथ खून/बलगम आया<br>Accompanied with phlegm/blood 3 | |
| | | ऊपर से कोई भी नहीं<br>None of the above 4 | |
| | | जवाब नही देना<br>Will not answer -888 | |
| | | पता नही<br>Does not know -999 | |

## फ.स्वास्थ्य (विस्तार )
## F: Health (Continued)

प्रश्नावली कोड/ Questionnaire code: |__|__| |__|__| |__|__| |__|__|__|

**जब से आपने टी.बी. कि गोलियॉ शुरू की है क्या आपके लक्षण कम हुए स्थिर रहे, या बढे?**

Since you started taking pills, have the following symptoms decreased, stayed stable, or increased?

| | | जब से आपने टी.बी. कि गोलियॉ शुरू की<br>कम हुए =१ स्थिर रहे =२ बढे =३ कुछ नहीं =4<br><br>Since you started taking pills<br>Decreased = 1 Stayed stable = 2 Increased = 3 None = 4 | | | | | |
|---|---|---|---|---|---|---|---|
| F.11 | **सांस की समस्याएं (जैसे खाँसी ,कठिन सांस, सीने में दर्द)**<br>Respiratory Problems (e.g. coughing, difficult/painful breakting, chest pains) | 1 | 2 | 3 | 4 | -888 | -999 |
| F.12 | **अन्य प्रकार के शरीर मे दर्द (जैसे सिर दर्द, पेट दर्द, दस्त, उल्टी, आंखों में दर्द, पीलिया, त्वचा की समस्याओं, लाल चकत्ते)**<br>Other types of body pain (e.g. headache, stomach pain, diarrhea, vomiting, eye pain, jaundice, skin problems, rash) | 1 | 2 | 3 | 4 | -888 | -999 |
| F.13 | **अन्य सामान्य स्वास्थ्य समस्याओं (जैसे बुखार, वजन घटाने, कमजोरी, पसीना, रक्ताल्पता, सुन्न होना)**<br>Other general health problems (e.g. fever, weight loss, weakness, sweats, anemia, numbness) | 1 | 2 | 3 | 4 | -888 | -999 |
| F.14 | **कोई अन्य स्वास्थ्य समस्या (विशेष)**<br>Any other health problem(SPECIFY) | 1 | 2 | 3 | 4 | -888 | -999 |

| | | | | |
|---|---|---|---|---|
| F.15 | **कुल मिलाकर, डॉट्स केंद्र की दवाई शुरू होने से पहले , क्या टी.बी. के लक्षण आपको अपने सामान्य काम(घर या कार्यस्थल पर ) को पूरा करने में बाधा डालते थे ?**<br><br>Altogether, before you started taking pills at the center, did the symptoms that you experienced prevent you from completing your usual activities, at home or at work? | **हाँ,महत्वपूर्ण रूप से**<br>Yes, significantly | 1 | |
| | | **हाँ,कुछ हद तक**<br>Yes, moderately | 2 | |
| | | **हाँ,बहुत कम**<br>Yes, very little | 3 | |
| | | **नहीं**<br>No | 4 | Skip to F.17 |
| | | **जवाब नही देना**<br>Will not answer | -888 | Skip to F.17 |

| | | | |
|---|---|---|---|
| F.16 | **कृपया निम्नलिखित लक्षणों को उस क्रम में रैंक करें जिसमें वे आपको गोलियाँ शुरू करने के 30 दिनों पहले प्रभावित करहे थे .शुरुवात उस लक्षण से करें जो आपकी दैनिक गतिविधियों को सबसे ज्यादा प्रभावित करता है.**<br>Please rank the following symptoms in the order in which they affected your daily activities at work and at home in the 30 days before you started taking pills at the center, beginning with the symptom that affected your daily activities the most.<br><br>**साक्षात्कारकर्ता:रोगी की द्वारा दी गई रैंकिंग को १ से ३ तक निर्धरित स्थान पर लिखें (१ उस लक्षण के लिए जिसने रोगी को सबसे अधिक प्रभावित किया ,३ से अधिक लक्षणों को रैंक न करें)**<br><br>*INTERVIEWER: Please write the patient's ranking from 1 up to 3 in the space provided (1 for the symptom that affected the patient the most; don't rank more than 3 symptoms)* | **रैंक**/RANK<br>**सांस की समस्याएं (जैसे खाँसी ,कठिन सांस, सीने में दर्द)**<br>Respiratory Problems (e.g. coughing, difficult/painful breathing, chest pains)<br><br>**अन्य प्रकार के शरीर में दर्द (जैसे सिर दर्द, पेट दर्द, दस्त, उल्टी, आंखों में दर्द, पीलिया, त्वचा की समस्याओं, लाल चकत्ते)**<br>Other types of body pain (e.g. headache, stomach pain, diarrhea, vomiting, eye pain, jaundice, skin problems, rash)<br><br>**अन्य सामान्य स्वास्थ्य समस्याओं (जैसे बुखार, वजन घटाने, कमजोरी, पसीना, रक्ताल्पता, सुन्न होना)**<br>Other general health problems (e.g. fever, weight loss, weakness, sweats, anemia, numbness)<br><br>**कोई अन्य समस्या स्वास्थ्य (विवरण दे)**<br>Any other health problem (*SPECIFY)*<br><br>**जवाब नहीं देना** -888<br>Will not answer<br><br>**पता नहीं** -999<br>Does not know | |
| F.17 | **दवाई शुरू होने के बाद से , टी.बी. के जो लक्षण आपने अनुभव किये,क्या उनकी वजह से आपको अपने सामान्य काम(घर या कार्यस्थल पर ) पूरा करने में समस्या आई ?**<br>Altogether, since you started taking pills at the center, have the symptoms that you experience prevented you from completing your usual activities, at home or at work? | **हाँ,महत्वपूर्ण रूप से** 1<br>Yes, significantly<br><br>**हां,कुछ हद तक** 2<br>Yes, moderately<br><br>**हां,बहुत कम** 3<br>Yes, very little<br><br>**नहीं** 4<br>No<br><br>**जवाब नही देना** -888<br>Will not answer | <br><br><br><br><br><br><br>Skip to G.0 |

| | | | |
|---|---|---|---|
| F.18 | **कृपया निम्नलिखित लक्षणों को उस क्रम में रैंक करें जिसमें वे आपको दैनिक गतिविधियों-घर और कार्यस्थल पर , प्रभावित कर रहे है जब से आपने दवा लेना शुरू की .शुरुवात उस लक्षण से करें जो आपकी दैनिक गतिविधियों को सबसे ज्यादा प्रभावित करता है**<br><br>Please rank the following symptoms in the order in which they affect your daily activities at work and at home since you started taking pills. Begin with the symptom that affect your daily activities the most.<br><br>**साक्षात्कारकर्ता:रोगी की द्वारा दी गई रैंकिंग को १ से ३ तक निर्धरित स्थान पर लिखें (१ उस लक्षण के लिए जिसने रोगी को सबसे अधिक प्रभावित किया ,३ से अधिक लक्षणों को रैंक न करें)**<br><br>INTERVIEWER: Please write the patient's ranking from 1 up to 3 in the space provided (1 for the symptom that affected the patient the most; don't rank more than 3 symptoms) | **रैंक** / RANK | |
| | | **सांस की समस्याएं (जैसे खाँसी ,कठिन सांस, सीने में दर्द)**<br>Respiratory Problems (e.g. coughing, difficult/painful breakting, chest pains) | |
| | | **अन्य प्रकार के शरीर में दर्द (जैसे सिर दर्द, पेट दर्द, दस्त, उल्टी, आंखों में दर्द, पीलिया, त्वचा की समस्याओं, लाल चकत्ते)**<br>Other types of body pain (e.g. headache, stomach pain, diarrhea, vomiting, eye pain, jaundice, skin problems, rash) | |
| | | **अन्य सामान्य स्वास्थ्य समस्याओं (जैसे बुखार, वजन घटाने, कमजोरी, पसीना, रक्ताल्पता, सुन्न होना)**<br>Other general health problems (e.g. fever, weight loss, weakness, sweats, anemia, numbness) | |
| | | **कोई अन्य समस्या स्वास्थ्य (विवरण दे)**<br>Any other health problem (*SPECIFY)* | |
| | | **जवाब नहीं देना**<br>Will not answer | -888 |
| | | **पता नहीं**<br>Does not know | -999 |

| ग: अन्य स्वास्थ्य सेवा प्रबन्धक के साथ पारस्पारिक प्रभाव<br>G: Interaction with other health care providers | | | प्रश्नावली कोड/Questionnaire code:<br>\|__\|__\| \|__\|__\| \|__\|__\| \|__\|__\|__\| |
|---|---|---|---|
| *अब, में आपसे कुछ सवाल आपके अन्य स्वास्थ्य कर्मचारी के साथ पारस्पारिक प्रभाव संबंधी, पूछूँगा*<br>***Now, I'd like to ask you some questions regarding your interactions with other health care providers*** | | | |
| G.0 | **टी बी की पुष्टि होने के बाद , क्या कोई स्वास्थ्य कर्मचारी आपके घर टी बी संबधित जानकारी देने आया था ?**<br><br>Has your house been visited by any health care workers providing information about TB since you were diagnosed? | **हां** Yes 1<br>**नही** No 2<br>**जवाब नही दिया** Will not answer -888<br>**मालूम नही** Does not know -999 | |
| G.1 | **टी बी की पुष्टि होने के बाद क्या आपके घर या परिवार के सदस्यों की टी.बी. जाँच की गयी है?**<br><br>Have your family / household members been tested for TB since you were diagnosed? | **हाँ सबकी** Yes, all 1<br>**हां कुछ की** Yes, some 2<br>**किसी की नही** None 3<br>**जवाब नही दिया** Will not answer -888<br>**मालूम नही** Does not know -999 | |
| G.2 | **पिछले तीन महिनें में क्या आप टी.बी. इलाज के अलावा किसी और कारण से डाक्टर या स्वास्थय सेवा प्रबन्धक से मिले है ?**<br><br>During the last 3 months, have you seen health care providers unrelated to your TB treatment? | **हाँ** Yes 1<br>**नहीं** No 2<br>**जवाब नही दिया** Will not say -888<br>**मालूम नहीं** Does not know -999 | Skip to **H.0** (for 2) |
| G.3 | **पिछले तीन महिनें में आप टी.बी. इलाज के अलावा किसी और कारण से डाक्टर या स्वास्थ्य सेवा प्रबन्धक से कितनी बार मिले है?**<br>During the last 3 months, how many times did you see a health care provider unrelated to your TB treatment? | ***संख्या*** *RECORD NUMBER* \|__\|__\| 1<br>**जवाब नही दिया** Will not say -888<br>**मालूम नहीं** Does not know -999 | |

| ग: अन्य स्वास्थ्य सेवा प्रबन्धक के साथ पारस्पारिक प्रभाव<br>G: Interaction with other health care providers | | | प्रश्नावली कोड/Questionnaire code:<br>\|__\|__\| \|__\|__\| \|__\|__\| \|__\|__\|__\| |
|---|---|---|---|
| हर परामर्श G.4- G.14 के लिए, प्रश्न को दुबारा पूछे. ग्रिड मे नोट करे<br>FOR EACH CONSULTATION, REPEAT QUESTIONS G.4-G.14. RECORD ON GRID | | | |
| G.4 | किस स्थिति या स्थितियों में आप डाक्टर या स्वास्थ्य सेवा प्रबन्धक से मिलने गये ?<br>For which condition or conditions did you make this visit?<br>बीमारी / परेशानी के कोड के लिए कोड बुकलेट का इस्तमाल करें. सभी उपयोगी कोड को दर्ज करें. ( अगर बीमारी/ अवस्था कोड बुकलेट में क्रमित नहीं हैं तो स्पष्ट कीजिए )<br>USE CONDITION CODES FROM THE CODE BOOKLET; RECORD ALL THAT APPLY (IF CONDITION IS NOT LISTED IN CODE BOOKLET, THEN SPECIFY) | \|__\|__\|__\| 1<br>\|__\|__\|__\| 2<br>\|__\|__\|__\| 3<br>\|__\|__\|__\| 4<br>स्पष्ट करें Other (Specify): -777<br>जवाब नहीं दिया Will not answer -888<br>मालूम नही Does not know -999 | |
| G.5 | आप किस तरह के स्वास्थ्य सेवा प्रबन्धक से मिले थे?<br>What type of health facility did you visit? | प्राथमिक स्वास्थ्य केंद्र/ सामुदायिक स्वास्थ्य केंद्र CHC / PHC 1<br>सरकारी निर्दिष्ट अस्पताल Gov't referral hospital 2<br>निजी अस्पताल Private hospital 3<br>टी बी अस्पताल T.B. hospital 4<br>उप स्वास्थ्य केन्द्र Aidpost / subcenter 5<br>डिस्पेन्सरी Dispensary 6<br>सरकारी डाक्टर Government doctor 7<br>प्राईवेट डाक्टर Private doctor 8<br>प्राईवेट कोम्पौंडर या नर्स Private compounder / nurse 9<br>सहायक नर्स प्रसविका ( ऐ.एन. एम) ANM (Auxiliary Nurse Midwife) 10<br>होमियोपैथिक Homeopathic 11<br>ऑगनबाड़ी Angwadi 12<br>ग्रामीण स्वास्थ्य कार्यकर्ता NGO Village health worker (NGO) 13<br>सरकारी ग्रामीण स्वास्थ्य कार्यकर्ता Village health worker (Gov't) 14<br>बंगाली डाक्टर Bengali doctor 15<br>स्वास्थ्य शिविर Health camp 16<br>भोपा / ओझा Bhopa / traditional healer 17 | |

| | | | | |
|---|---|---|---|---|
| | | अन्य(स्पष्ट करे)<br>Other (specify): | -777 | |
| | | जवाब नहीं दिया<br>Will not answer | -888 | |
| | | मालूम नही<br>Does not know | -999 | |
| G.6 | आप इसी व्यक्ति/ स्वास्थ्य सेवा प्रबन्धक के पास क्यों गये ?<br><br>Why did you go to this person/ health care provider?<br><br>साक्षात्कारकर्ता : कृपया जवाब बोले नहीं, जो लागू हो सभी पर गोला लगायें।<br><br>INTERVIEWER: DO NOT PROMPT, CIRCLE ALL THAT APPLY | इस छेत्र में यही है।<br>Only one in the area | 1 | |
| | | घर या दफ़्तर से सबसे नजदीक है<br>Closest from my house / office | 2 | |
| | | लोगों ने सुझाया था।<br>Recommended | 3 | |
| | | पैसे ठीक लगते है ।<br>Good price | 4 | |
| | | शुरु से यहीं जाता हूँ।<br>Have always been there | 5 | |
| | | भरोसेमंद<br>Reliable | 6 | |
| | | अन्य(स्पष्ट करें)<br>Other (specify): | -777 | |
| | | जवाब नहीं दिया<br>Will not say | -888 | |
| | | मालूम नहीं<br>Does not know | -999 | |

| | | | | | |
|---|---|---|---|---|---|
| G.7 | अगर आपको निम्नलिखित सुविधा मिली तो क्या उसके लिए आपने पैसे भी दिए थे? स्पष्ट कीजिए.<br>State if you received any of the following benefits and payment made if any | हाँ<br>YES | नहीं<br>NO | खर्चा( अगर कोई )<br>Payment, if any | **Will not answer (-888)/ Doesn't Know (-999)** |
| | a सलाह<br>Consultation | 1 | 2 | \|__\|__\|__\| | |
| | b दवाईयाँ जो स्वास्थ्य सेवा प्रबन्धक से मिली<br>Medication given in the facility | 1 | 2 | \|__\|__\|__\| | |
| | c दवाईयाँ बाहर से खरीदी<br>Medication bought outside | 1 | 2 | \|__\|__\|__\| | |
| | d इंजेक्शन या सुई<br>Injection | 1 | 2 | \|__\|__\|__\| | |
| | e ड्रिप/ पानी चढ़ना<br>Drip | 1 | 2 | \|__\|__\|__\| | |
| | f ऑपरेशन<br>Operation | 1 | 2 | \|__\|__\|__\| | |
| | g लैब जाँच<br>Lab test | 1 | 2 | \|__\|__\|__\| | |
| | h अन्य इलाज<br>Other treatment | 1 | 2 | \|__\|__\|__\| | |
| | i आने जाने का खर्चा<br>Transportation (round trip) | 1 | 2 | \|__\|__\|__\| | |

| | | | | |
|---|---|---|---|---|
| G.8 | **परामर्श के दौरान अपनी तबियत के बारें में बताने में आप कितने सहज थे?**<br>How comfortable did you feel to talk about your condition during the consultation?<br><br>**साक्षात्कारकर्ता: दुबारा जाँच करें कि रोगी को सलाह मिली या नहीं।**<br><br>INTERVIEWER: CHECK THAT THE PATIENT HAS RECEIVED CONSULTATION | **बहुत सहजता से**<br>Very comfortable | 1 | Skip to G.**10** |
| | | **ठीक- ठीक सहजता से**<br>Quite comfortable | 2 | Skip to G.**10** |
| | | **उतनी सहजता से नहीं**<br>Not so comfortable | 3 | |
| | | **बिल्कुल असहजता से ।**<br>Not comfortable at all | 4 | |
| | | **जवाब नहीं दिया**<br>Will not say | -888 | Skip to G.**10** |
| | | **मालूम नहीं**<br>Does not know | -999 | Skip to G.**10** |

प्रश्नावली कोड/Questionnaire code: |__|__| |__|__| |__|__| |__|__|__|

| | | | |
|---|---|---|---|
| G.9 | आप जाँच के दौरान सहज क्यों नहीं थे ?<br><br>Why did you not feel comfortable during the consultation?<br><br>Circle all that apply<br>जो लागू हो उन सब पर घेरा बनाये<br><br>साक्षात्कारकर्ता: दुबारा जाँच करें कि रोगी को सलाह मिली या नहीं।<br><br>INTERVIEWER: CHECK THAT THE PATIENT HAS RECEIVED CONSULTATION | उस कमरें में और लोग थे<br>There were other people in the room 1<br><br>तबियत ठीक नहीं लग रही थी।<br>I was feeling unwell 2<br><br>अपने बारें में बात करने में शर्म आ रही<br>I felt ashamed to talk about my condition 3<br><br>डाक्टर ध्यान नहीं दे रहा था ।<br>The doctor was not really paying attention 4<br><br>अन्य (स्पष्ट करें)<br>Other (please specify) -777<br><br>जवाब नहीं दिया<br>Will not say -888<br><br>मालूम नहीं<br>Does not know -999 | |
| G.10 | क्या आपको लगता है कि इस व्यक्ति/ स्वास्थ्य सेवा प्रबन्धक के पास डाक्टर के रूप में सही योग्यता और प्रशिक्षण है ?<br><br>Do you feel that this person has good training and good qualifications as a doctor? | हाँ<br>Yes 1<br><br>नहीं<br>No 2<br><br>जवाब नहीं दिया<br>Will not answer -888<br><br>मालूम नहीं<br>Does not know -999 | |
| G.11 | आप कितने निश्चिन्त है कि आपकी हालत को डाक्टर / स्वास्थ्य सेवा प्रबन्धक द्वारा गंभीरता से लिया गया ?<br>How confident are you that your condition was taken seriously by the health worker / doctor? | पूरी तरह निश्चिन्त<br>Very confident 1<br><br>निश्चिन्त<br>Quite confident 2<br><br>उतना निश्चिन्त नहीं<br>Not so confident 3<br><br>बिल्कुल निश्चिन्त नहीं<br>Not confident at all 4<br><br>जवाब नहीं दिया<br>Will not say -888<br><br>मालूम नहीं<br>Does not know -999 | |

प्रश्नावली कोड/Questionnaire code: |__|__| |__|__| |__|__| |__|__|__|

| | | | |
|---|---|---|---|
| G.12 | क्या स्वास्थ्यकर्मी / डाक्टर ने आपको फिर दो महिनें में बुलाया है ?<br><br>Did the doctor / health worker invite you to visit again in the next 2 months? | हाँ<br>Yes 1<br><br>नही<br>No 2<br><br>जवाब नही दिया<br>Will not answer -888<br><br>मालूम नही<br>Does not know -999 | |
| G.13 | क्या आप दुबारा गये थे ?<br><br>Did you visit again? | हाँ<br>Yes 1<br><br>नही<br>No 2<br><br>जवाब नही दिया<br>Will not answer -888<br><br>मालूम नही<br>Does not know -999 | |
| G.14 | क्या आप दुबारा जाने की सोच रहे हैं ?<br><br>Are you planning to visit again? | हाँ<br>Yes 1<br><br>नही<br>No 2<br><br>जवाब नही दिया<br>Will not answer -888<br><br>मालूम नही<br>Does not know -999 | |
| G.15 | आपको जो स्वास्थ्य सेवायें मिलती है आप कुल मिलाकर उससे कितना संतुष्ट है ?<br><br>Overall, how satisfied are you with the health services that you received? | बहुत संतुष्ट<br>Very satisfied 1<br><br>संतुष्ट<br>Quite satisfied 2<br><br>बहुत संतुष्ट नहीं<br>Not very satisfied 3<br><br>बिल्कुल संतुष्ट नहीं<br>Not satisfied at all 4<br><br>जवाब नहीं दिया<br>Will not say -888<br><br>मालूम नहीं<br>Does not know -999 | |

| ग: अन्य स्वास्थ्य सेवा प्रबन्धक के साथ पारस्पारिक प्रभाव<br>**G: Interaction with other health care providers** | | | | प्रश्नावली कोड/Questionnaire code:<br>\|__\|__\| \|__\|__\| \|__\|__\| \|__\|__\|__\| |
|---|---|---|---|---|
| | परामर्श 1<br>CONSULTATION 1 | **परामर्श 2**<br>CONSULTATION 2 | **परामर्श 3**<br>CONSULTATION 3 | **परामर्श 4**<br>CONSULTATION 4 |
| G.4 | \|__\|__\|__\| 1 | \|__\|__\|__\| 1 | \|__\|__\|__\| 1 | \|__\|__\|__\| 1 |
| | \|__\|__\|__\| 2 | \|__\|__\|__\| 2 | \|__\|__\|__\| 2 | \|__\|__\|__\| 2 |
| | \|__\|__\|__\| 3 | \|__\|__\|__\| 3 | \|__\|__\|__\| 3 | \|__\|__\|__\| 3 |
| | \|__\|__\|__\| 4 | \|__\|__\|__\| 4 | \|__\|__\|__\| 4 | \|__\|__\|__\| 4 |
| | **स्पष्ट करें** -777 | **स्पष्ट करें** -777 | **स्पष्ट करें** -777 | **स्पष्ट करें** -777 |
| | Specify: | Specify: | Specify: | Specify: |
| | -888 | -888 | -888 | -888 |
| | -999 | -999 | -999 | -999 |
| G.5 | 1 | 1 | 1 | 1 |
| | 2 | 2 | 2 | 2 |
| | 3 | 3 | 3 | 3 |
| | 4 | 4 | 4 | 4 |
| | 5 | 5 | 5 | 5 |
| | 6 | 6 | 6 | 6 |
| | 7 | 7 | 7 | 7 |
| | 8 | 8 | 8 | 8 |
| | 9 | 9 | 9 | 9 |
| | 10 | 10 | 10 | 10 |
| | 11 | 11 | 11 | 11 |
| | 12 | 12 | 12 | 12 |
| | 13 | 13 | 13 | 13 |
| | 14 | 14 | 14 | 14 |
| | 15 | 15 | 15 | 15 |
| | 16 | 16 | 16 | 16 |
| | 17 | 17 | 17 | 17 |

| | | | | |
|---|---|---|---|---|
| | -777 | -777 | -777 | -777 |
| | | | | |
| | -888 | -888 | -888 | -888 |
| | -999 | -999 | -999 | -999 |
| G.6 | 1 | 1 | 1 | 1 |
| | 2 | 2 | 2 | 2 |
| | 3 | 3 | 3 | 3 |
| | 4 | 4 | 4 | 4 |
| | 5 | 5 | 5 | 5 |
| | 6 | 6 | 6 | 6 |
| | -777 | -777 | -777 | -777 |
| | | | | |
| | -888 | -888 | -888 | -888 |
| | -999 | -999 | -999 | -999 |

| G.7 | हाँ | नहीं | खचा (अगर कोई) | हाँ | नहीं | खचा( अगर कोई) | हाँ | नहीं | खचा( अगर कोई ) | हाँ | नहीं | खचा( अगर कोई ) |
|---|---|---|---|---|---|---|---|---|---|---|---|---|
| | YES | NO | Payment | YES | NO | Payment | YES | NO | Payment | YES | NO | Payment |
| a | 1 | 2 | | 1 | 2 | | 1 | 2 | | 1 | 2 | |
| b | 1 | 2 | | 1 | 2 | | 1 | 2 | | 1 | 2 | |
| c | 1 | 2 | | 1 | 2 | | 1 | 2 | | 1 | 2 | |
| d | 1 | 2 | | 1 | 2 | | 1 | 2 | | 1 | 2 | |
| e | 1 | 2 | | 1 | 2 | | 1 | 2 | | 1 | 2 | |
| f | 1 | 2 | | 1 | 2 | | 1 | 2 | | 1 | 2 | |
| g | 1 | 2 | | 1 | 2 | | 1 | 2 | | 1 | 2 | |
| h | 1 | 2 | | 1 | 2 | | 1 | 2 | | 1 | 2 | |
| i | 1 | 2 | | 1 | 2 | | 1 | 2 | | 1 | 2 | |

| | | | | | | | | |
|---|---|---|---|---|---|---|---|---|
| G.8 | 1 | Skip to G.**10** | 1 | Skip to G.**10** | 1 | Skip to G.**10** | 1 | Skip to G.**10** |
| | 2 | Skip to G.**10** | 2 | Skip to G.**10** | 2 | Skip to G.**10** | 2 | Skip to G.**10** |
| | 3 | | 3 | | 3 | | 3 | |
| | 4 | | 4 | | 4 | | 4 | |
| | -888 | Skip to G.**10** | -888 | Skip to G.**10** | -888 | Skip to G.**10** | -888 | Skip to G.**10** |
| | -999 | Skip to G.**10** | -999 | Skip to G.**10** | -999 | Skip to G.**10** | -999 | Skip to G.**10** |

| | | | | |
|---|---|---|---|---|
| G.9 | 1 | 1 | 1 | 1 |
| | 2 | 2 | 2 | 2 |
| | 3 | 3 | 3 | 3 |
| | 4 | 4 | 4 | 4 |
| | -777 | -777 | -777 | -777 |
| | -888 | -888 | -888 | -888 |
| | -999 | -999 | -999 | -999 |
| G.10 | 1 | 1 | 1 | 1 |
| | 2 | 2 | 2 | 2 |
| | -888 | -888 | -888 | -888 |
| | -999 | -999 | -999 | -999 |
| G.11 | 1 | 1 | 1 | 1 |
| | 2 | 2 | 2 | 2 |
| | 3 | 3 | 3 | 3 |
| | 4 | 4 | 4 | 4 |
| | -888 | -888 | -888 | -888 |
| | -999 | -999 | -999 | -999 |

| | | | | |
|---|---|---|---|---|
| G.12 | 1 | 1 | 1 | 1 |
| | 2 | 2 | 2 | 2 |
| | -888 | -888 | -888 | -888 |
| | -999 | -999 | -999 | -999 |
| G.13 | 1 | 1 | 1 | 1 |
| | 2 | 2 | 2 | 2 |
| | -888 | -888 | -888 | -888 |
| | -999 | -999 | -999 | -999 |
| G.14 | 1 | 1 | 1 | 1 |
| | 2 | 2 | 2 | 2 |
| | -888 | -888 | -888 | -888 |
| | -999 | -999 | -999 | -999 |

## ह.टीकाकरण और पूर्व टी.बी.अनुभव
## H: Vaccination and past TB

प्रश्नावली कोड/Questionnaire code: |__|__| |__|__| |__|__| |__|__|__|

*साक्षात्कारकर्तारू अब आपके टी॰बी॰ के अनुभव से जुड़े कुछ प्रश्न*

*INTERVIEWER: The next set of questions concern your experience with tuberculosis.*

| | | | | |
|---|---|---|---|---|
| H.0 | **क्या आपको बी.सी.जी. का टीका लगा है?** Have you been vaccinated with BCG? | **हाँ** Yes | 1 | |
| | | **नहीं** No | 2 | Skip to **H.4** |
| | | **क्या आपको टीका लगा पर आप आश्वस्त नहीं है की उसमें बी.सी.जी.निरोधक टीका भी** Have been vacinated but not certain if it included the BCG vaccine | 3 | |
| | | **जवाब नहीं देना** Will not answer | -888 | Skip to **H.4** |
| | | **पता नहीं** Does not know | -999 | Skip to **H.4** |
| H.1 | **क्या आप शरीर पर लगे टीके का निशान मुझे दिखाना चाहेंगे?** Would you mind showing me the scar of the vaccine? | **निशान द्रश्य है** Mark visible | 1 | |
| | | **निशान अद्रश्य है** Mark non visible | 2 | |
| | | **निशान नहीं दिखाना चाहेंगे** Does not want to show | 3 | |
| H.2 | **आपको टीका किसने लगाया था ?** Who administered the vaccine? | **सरकारी डॉक्टर** Government Doctor / MO | 1 | |
| | | **सरकारी कम्पाउन्डर** Government Compounder | 2 | |
| | | **सरकारी दावा खाने से** Government Pharmacist | 3 | |
| | | **एम्.पी.डब्लू** MPW | 4 | |
| | | **ए.एन.एम्. / सरकारी सहायिका नुर्स/ प्रसाविका** ANM | 5 | |
| | | **स्टाफ़ नर्स** Staff nurse | 6 | |
| | | **लेडी हेल्थ विसीटर** LHV | 7 | |
| | | **निजी डॉक्टर** Private Doctor | 8 | |
| | | **बंगाली डॉक्टर** Bengali doctor | 9 | |
| | | **निजी कम्पाउन्डर** Pivate compounder | 10 | |
| | | **निजी औषध विक्रेता** Private pharmacist | 11 | |
| | | **धाईं** TBA / Daima | 12 | |
| | | **गाव स्वस्थ्य सेवक** VHW / CHW | 13 | |
| | | **भोपा** Bhopa | 14 | |
| | | **स्कूल शिक्षक /आंगनवाड़ी कार्यकर्ता** School teacher / anganwadi worker | 15 | |
| | | **अन्य न. जी. ओ.** Other NGO | 16 | |
| | | **अन्य(विवरण दे)** Other. Please specify | -777 | |
| | | **जवाब नहा** Will not answer | -888 | |
| | | **पता नहीं** Does not know | -999 | |

प्रश्नावली कोड/Questionnaire code: |__|__| |__|__| |__|__| |__|__|__|

| | | | | |
|---|---|---|---|---|
| H.3 | **यह टीकाकरण कहां हुआ?**<br>Where was this vaccine given? | **प्राथमिक स्वस्थ्य केंद्र / सामुदायिक स्वस्थ्य**<br>CHC / PHC | 1 | |
| | | **सरकारी अस्पताल**<br>Government Hospital | 2 | |
| | | **निजी अस्पताल**<br>Private Hospital | 3 | |
| | | **आयुर्वेदिक अस्पताल**<br>Ayurvedic Hospital | 4 | |
| | | **टी.बी.अस्पताल**<br>TB Hospital | 5 | |
| | | **दवाई की दूकान**<br>Medicine Shop | 6 | |
| | | **सहायता केंद्र**<br>Aid Post | 7 | |
| | | **आंगनवाड़ी/बालवाड़ी/स्कूल**<br>Anganwadi / Balwadi / School | 8 | |
| | | **स्वास्थ्य शिविर**<br>Health Camp | 9 | |
| | | **एन.जी.ओ. क्लीनिक**<br>NGO Clinic | 10 | |
| | | **सरकारी मोबाइल** क्लीनिक<br>Government Mobile Clinic | 11 | |
| | | **घर**<br>Home | 12 | |
| | | **सामुदायिक केंद्र**<br>Community Center | 13 | |
| | | **अन्य(विवरण द)** ____________________<br>Other. Please specify | -777 | |
| | | **जवाब नहा**<br>Will not answer | -888 | |
| | | **पता नहा**<br>Does not know | -999 | |

| | | | |
|---|---|---|---|
| H.4 | **क्या इससे पहले भी आपको कभी टी.बी. हुआ है?**<br>Have you had TB before the present occurrence? | **हा** Yes — 1<br>**अगर हाँ,तो कितनी बार?**<br>If yes, how many times? ☐☐ | |
| | | **नहीं** No — 2 | Skip to **I.0** |
| | | **जवाब नहीं देना** Will not answer — -888 | Skip to **I.0** |
| | | **पता नहीं** Does not know — -999 | Skip to **I.0** |
| **अब,मैं आपसे आपके टी.बी. के इससे पहले के अनुभव से जुड़े कुछ प्रश्न पूछूँगा**<br>*Now: I am going to ask you questions about the last time you had TB BEFORE this occurrence.* | | | |
| H.5 | **इससे पहले कब आपको टी.बी. हुआ था?**<br>When were you previously diagnosed with the disease? | **माह**/month ☐☐ **वर्ष**/year ☐☐☐☐ — 1 | |
| | | **जवाब नहीं देना** Will not answer — -888 | |
| | | **पता नहीं** Does not know — -999 | |
| H.6 | **पिछली बार टी.बी. आपके फेफडों में था या फेफडो से बाहर?यदि बाहर,तो कहा?**<br>Was this previous occurrence in your lungs or outside your lungs? If outside, where?<br>INTERVIEWER: DO NOT PROMPT<br>**सक्षत्कार्यकर्ता: कपया जवाब पढ कर ना सनाए** | **फेफडो मे** Lungs (Pulmonary) — 1 | |
| | | **फेफडो से बाहर** Outside lungs (Extra Pulmonary) — 2<br>**विवरण दे,कहाँ?**<br>Specify where | |
| | | **जवाब नहीं देना** Will not answer — -888 | |
| | | **पता नहीं** Does not know — -999 | |

| | | | | |
|---|---|---|---|---|
| H.7 | **क्या अपने टी.बी. संक्रमण को ठीक करने के लिए गोलियाँ ली थी?** Did you take pills to treat your last TB infection? | **हाँ** Yes | 1 | |
| | | **नहीं** No | 2 | Skip to **H.10** |
| | | जवाब नहीं देना Will not answer | -888 | Skip to **H.10** |
| | | **पता नहीं** Does not know | -999 | Skip to **H.10** |
| H.8 | **आपने वे गोलियाँ कहा से ली थी?** Where did you take them? | **निजी डॉक्टर** Private doctor | 1 | |
| | | **सरकारी डॉक्टर** Govt. Doctor | 2 | |
| | | **सरकारी डाट्स कद्र** Govt DOTS center | 3 | |
| | | **एन.जी.ओ.संचालित डॉट्स केंद्र** NGO-run DOTS center | 4 | |
| | | **घर पर** At home | 5 | |
| | | **अन्य(विवरण दे)** ____ Other (specify) | -777 | |
| | | **जवाब नहीं देना** Will not answer | -888 | |
| | | **पता नहीं** Does not know | -999 | |
| H.9 | **आपका इलाज कितने समय चला?** How long did your treatment last? **साक्षात्कारकर्ता :दिनों की संख्यां लिखें यदि इलाज २ माह से कम चला हो** *INTERV* IEWER: Use days if treatment lasted less *than two months* | **माह/** Months [ ][ ] | 1 | |
| | | **दिन/** Days [ ][ ] | 2 | |
| | | **जवाब नहीं देना** Will not answer | -888 | |
| | | **पता नहीं** Does not know | -999 | |

| | | | |
|---|---|---|---|
| H.10 | **टी.बी. के पिछले संक्रमण का क्या परिणाम रहा**<br>What was the outcome of your last infection with TB? | **उपचार पूरा हुआ, एक्स-रे या बलगम जांच द्वारा पुष्टि**<br>Cured, confirmed by X-ray and/ or sputum test 1 | Skip to **H.12** |
| | | **उपचार पूरा हुआ पर टी.बी. ठीक नहीं हुआ**<br>Treatment completed but not cured 2 | Skip to **H.12** |
| | | **उपचार को बीच में रोकना पड़ा**<br>Treatment interrupted 3 | |
| | | **अन्य(विवरण दें)** ____________<br>Other (specify) -777 | |
| | | **जवाब नहीं देना**<br>Will not answer -888 | Skip to **H.12** |
| | | **पता नहीं**<br>Does not know -999 | Skip to **H.12** |
| H.11 | **उपचार बीच में क्यों रुका?**<br>Why was the treatment interrupted?<br><br>**जो भी लागू होता है उस पर घेरा बनाये**<br>*CIRCLE ALL THAT APPLY* | **मुझे स्वास्थ्य मे सुधार लगा और मैंने गोलियाँ लेनी बंद कर दी**<br>I was feeling better so I stopped taking pills 1 | |
| | | **मुझे कहीं और जाकर बसना पड़ा**<br>I had to move to another place 2 | |
| | | **मुझे लगा की उपचार पूरा हो गया**<br>I thought the treatment was finished 3 | |
| | | **गोलियों का सेवन करने के लिए मैं बहुत कमज़ोर था**<br>I was too weak to keep taking pills 4 | |
| | | **१ या २ बार गोली लेना भूल गया उसके बाद लगा गोली खाना व्यर्थ होगा**<br>I forgot 1 or 2 times and thought it was then useless to continue 5 | |
| | | **दवाइ बहुत गरम थी/मुझे दुशप्रभाव हुए थे**<br>Effect of taking pill was too strong/I had side effects 6 | |
| | | **अन्य(विवरण दें)**<br>Other (specify) -777 | |
| | | **जवाब नहीं देना**<br>Will not answer -888 | |
| | | **पता नहीं**<br>Does not know -999 | |
| H.12 | **आपको क्या लगता है उपचार पूरा होने के बाद आपकी स्थिति सुधरी ?**<br>Do you think that your condition improved at the end of that treatment?<br><br>*INTERVIEWER: This question refers to both interrupted and completed prior treatments*<br><br>**साक्षात्कारकर्ता: यह प्रश्न दोनों बाधित और पूरा पूर्व उपचार के लिए है** | **हाँ,बहुत**<br>Yes, a lot 1 | |
| | | **हाँ,कुछ-कुछ**<br>Yes, somewhat 2 | |
| | | **कुछ खास नहीं**<br>Not really 3 | |
| | | **बिलकुल नहीं**<br>Not at all 4 | |
| | | **जवाब नहीं देना**<br>Will not answer -888 | |
| | | **पता नहीं**<br>Does not know -999 | |

| | | | |
|---|---|---|---|
| H.13 | क्या आप इस बार भी उसी स्वास्थ्य प्रबंधक के पास गए जिसकी सेवाएं आपने पिछली बार ली थी?<br>Did you revisit the same health care provider that you had the last time, for this illness? | हाँ<br>Yes 1 | Skip to **I.0** |
| | | नहीं<br>No 2 | |
| | | जवाब नहीं देना<br>Will not answer -888 | Skip to **I.0** |
| | | पता नहीं<br>Does not know -999 | Skip to **I.0** |
| H.14 | अगर आप इस बार किसी नए स्वास्थ्य प्रबंधक के पास गए ,तो ऐसा करने के कारणों की सूची बनाये.<br>If you went to a different health provider please list your reasons.<br>साक्षात्कारकर्ता: उन सभी पर घेरा बनाये जो लागू होते है<br>INTERVIEWER: Circle all that apply | नए स्वास्थ्य प्रबंध की दवाइयां अच्छी है 1<br>This provider had better medicines | |
| | | यहाँ जाने की सलाह एक दोस्त/परिवार के सदय ने दी जिसे टी.बी.हुआ था 2<br>Was advised to go here by another friend/ family member who had TB | |
| | | उपचार कम समय का था 3<br>The treatment was for a shorter period | |
| | | नए स्वास्थ्य प्रबंधक की सेवाएं सस्ती/मुफ्त थी 4<br>This treatment/ provider was cheaper/free | |
| | | यह उपचार ज्यादा विश्वसनीय था 5<br>This treatment was most trustworthy/reliable | |
| | | क्योंकि मैं दूसरी जगह चला गया<br>Because I moved 6 | |
| | | -777<br>अन्य(विवरण दे)<br>Other (specify) | |
| | | जवाब नहीं देना -888<br>Will not answer | |
| | | पता नहीं -999<br>Does not know | |

| आई.पूर्व उपचार और जांच<br>**I: Earlier treatment and detection** | प्रश्नावली कोड<br>/Questionnaire code: \|__\|__\| \|__\|__\| \|__\|__\| \|__\|__\|__\| |
|---|---|

**अब, मैं आपसे आपके वर्तमान के संक्रमण के बारे में कुछ प्रश्न पूछूँगा.**
Now, I'd like to ask you some questions regarding your current disease.

| | | | | |
|---|---|---|---|---|
| I.0 | **क्या आपने टीबी के लक्षण दिखने के बाद किसी एक या अन्य स्वास्थ्य कर्मचारी से सलाह ली? अगर हा तो कितने?**<br>Did you consult one or several doctors and/or health workers regarding your TB symptoms? If yes, how many? | **हां**<br>Yes ☐☐ | 1 | |
| | | **नहीं**<br>No | 2 | Skip to **I.7** |
| **हर स्वाथ्य कर्मचारी के लिए, प्रश्न I.1 - I.6 को दुबारा पूछे. ग्रिड मे नोट करे**<br>FOR EACH HEALTH PROVIDER CONSULTED, REPEAT QUESTIONS I.1 - I.6. RECORD ON GRID | | | | |
| I.1 | **आपने टीबी के लक्षण दिखने के बाद कौन से स्वाथ्य कर्मचारी से सलाह ली?**<br>Which health worker did you consult for these symptoms? | **निजी डॉक्टर**<br>Private doctor | 1 | |
| | | **भोपा**<br>A bhopa | 2 | |
| | | **ऑपरेशन आशा परामर्शदाता**<br>Operation ASHA Counselor | 3 | |
| | | **ऐ. एन.एम**<br>ANM | 4 | |
| | | **आंगनवाडी कर्मचारी**<br>Anganwadi worker | 5 | |
| | | **अन्य सरकारी स्वास्थ्य कर्मचारी**<br>Other government health worker | 6 | |
| | | **अन्य(विवरण दें)** ________<br>Other (specify) | -777 | |
| | | **जवाब नहीं देना**<br>Will not answer | -888 | Skip to **I.7** |
| | | **पता नहीं**<br>Does not know | -999 | Skip to **I.7** |
| I.2 | **इस स्वास्थ्य कर्मचारी ने क्या किया?**<br>What did this health worker do?<br><br>**उन सब पर घेरा बनाये जो लागू होते है**<br>CIRCLE ALL THAT APPLY<br><br>**एक से जायदा स्किप पर सबसे नज़दीक के प्रशन पर जाए**<br>In case of multiple skips, skip to the lowest numbered question | **गोली दिया**<br>Gave me tablets | 1 | Skip to **I.4** |
| | | **सिरप दिया**<br>Gave me syrup | 2 | Skip to **I.4** |
| | | **सुई लगाई/ड्रिप**<br>Gave me an injection/drip | 3 | Skip to **I.4** |
| | | **टी.बी.का उपचार किया**<br>Gave me TB treatment | 4 | Skip to **I.4** |
| | | **मुझे टी.बी. की जांच कराने को कहा**<br>Told me to get detected for TB | 5 | Skip to **I.6** |
| | | **मुझे अन्य स्वास्थ्य कर्मचारी से सलाह लेने को कहा**<br>Directed me to another health worker | 6 | |
| | | **कुछ नहीं किया**<br>Did nothing | 7 | Skip to **I.6** |
| | | **अन्य(विवरण दे)** ________<br>Other (specify) | -777 | Skip to **I.4** |
| | | **जवाब नहीं देना**<br>Will not answer | -888 | Skip to **I.6** |
| | | **पता नहीं**<br>Does not know | -999 | Skip to **I.6** |

| | | | | |
|---|---|---|---|---|
| I.3 | **जिससे आपने सबसे पहले सलाह ली थी,उस व्यक्ति ने आपको किससे सलाह लेने को कहा था?**<br>To whom did this first person you consulted direct you? | **निजी डॉक्टर**<br>private doctor | 1 | |
| | | **भोपा**<br>A bhopa | 2 | |
| | | **ऑपरेशन आशा सलाहकार**<br>Operation ASHA Counselor | 3 | |
| | | **ऐ.एन.एम**<br>ANM | 4 | |
| | | **आंगनवाडी कर्मचारी**<br>Anganwadi worker | 5 | |
| | | **अन्य सरकारी स्वास्थ्य कर्मचारी**<br>Other government health worker | 6 | |
| | | **अन्य(विवरण दे)** ______<br>Other (specify) | -777 | |
| | | **जवाब नहीं देना**<br>Will not answer | -888 | |
| | | **पता नहीं**<br>Does not know | -999 | |
| I.4 | **क्या आपको उपचार कराने क लिए दोबारा किसी से सलाह लेनी पड़ी?**<br>Did you have to consult again for taking this treatment? | **हॉ,एक बार**<br>Yes, once | 1 | |
| | | **हॉ,२-५ बार**<br>Yes, 2-5 times | 2 | |
| | | **हाँ,५-१० बार**<br>Yes, 5-10 times | 3 | |
| | | **हाँ,१० से ज्यादा बार**<br>Yes, more than 10 times | 4 | |
| | | **नहीं**<br>No | 5 | |
| | | **जवाब नहीं देना**<br>Will not answer | -888 | |
| | | **पता नहीं**<br>Does not know | -999 | |

प्रश्नावली कोड
/Questionnaire code: |__|__| |__|__| |__|__| |__|__|__|

| | | | | |
|---|---|---|---|---|
| I.5 | आपका उपचार कितने समय चला ?<br>How long did you take a treatment?<br><br>साक्षात्कारकर्ता :दिनों की संख्यां लिखें यदि इलाज २ माह से कम चला हो<br>INTERVIEWER: Use days if treatment lasted less *than two months* | माह/ Months [ ][ ] | 1 | |
| | | दिन/ Days [ ][ ] | 2 | |
| | | जवाब नहीं देना<br>Will not answer | -888 | |
| | | पता नहीं<br>Does not know | -999 | |
| I.6 | आपको इस उपचार और परामर्श के लिए कुल कितना खर्च करना पड़ा?<br>How much total have you paid for this treatment and consultations? | रू./Rs. [ ][ ][ ][ ] | 1 | |
| | | जवाब नहीं देना<br>Will not answer | -888 | |
| | | पता नहीं<br>Does not know | -999 | |
| I.7 | आपको टी.बी.की जांच कराने की सलाह किसने दी?<br>Who advised you to go and get detected for TB?<br><br>उन सभी पर घेरा बांये जो लागू होते है.<br>CIRCLE ALL THAT APPLY | किसी ने नहीं ,मैंने ख़ुद पहल की<br>Nobody, it was my own initiative | 1 | |
| | | निजी डॉक्टर<br>Private doctor | 2 | |
| | | भोपा<br>A bhopa | 3 | |
| | | ऐ.एन.एम<br>ANM | 4 | |
| | | आंगनवाड़ी<br>Anganwadi | 5 | |
| | | अन्य सरकारी स्वास्थ्य कर्मचारी<br>Other government health worker | 6 | |
| | | ऑपेरशन आशा सलाहकार<br>Operation ASHA Counselor | 7 | |
| | | मेरे पति/पत्नी/अभिभावक/संतान<br>My spouse/parents/children | 8 | |
| | | दोस्त/रिश्तेदार<br>A friend/relative | 9 | |
| | | अन्य(विवरण दे) ________<br>Other (specify) | -777 | |
| | | जवाब नहीं देना<br>Will not answer | -888 | |
| | | पता नहीं<br>Does not know | -999 | |

## आई.पूर्व उपचार और जांच
## I: Earlier treatment and detection

प्रश्नावली कोड /Questionnaire code: |__|__||__|__||__|__||__|__|__|

**हर स्वाथ्य कर्मचारी के लिए, प्रश्न I.1 - I.6 को दुबारा पूछे. ग्रिड मे नोट करे**
FOR EACH HEALTH PROVIDER CONSULTED, REPEAT QUESTIONS I.1 - I.6. RECORD ON GRID

| | | | | |
|---|---|---|---|---|
| I.1 | 1 | 1 | 1 | 1 |
| | 2 | 2 | 2 | 2 |
| | 3 | 3 | 3 | 3 |
| | 4 | 4 | 4 | 4 |
| | 5 | 5 | 5 | 5 |
| | 6 | 6 | 6 | 6 |
| | -777 | -777 | -777 | -777 |
| | -888 Skip to **I.7** | -888 Skip to **I.7** | -888 Skip to **I.7** | -888 Skip to **I.7** |
| | -999 Skip to **I.7** | -999 Skip to **I.7** | -999 Skip to **I.7** | -999 Skip to **I.7** |
| I.2 | 1 Skip to **I.4** | 1 Skip to **I.4** | 1 Skip to **I.4** | 1 Skip to **I.4** |
| | 2 Skip to **I.4** | 2 Skip to **I.4** | 2 Skip to **I.4** | 2 Skip to **I.4** |
| | 3 Skip to **I.4** | 3 Skip to **I.4** | 3 Skip to **I.4** | 3 Skip to **I.4** |
| | 4 Skip to **I.4** | 4 Skip to **I.4** | 4 Skip to **I.4** | 4 Skip to **I.4** |
| | 5 Skip to **I.6** | 5 Skip to **I.6** | 5 Skip to **I.6** | 5 Skip to **I.6** |
| | 6 | 6 | 6 | 6 |
| | 7 Skip to **I.6** | 7 Skip to **I.6** | 7 Skip to **I.6** | 7 Skip to **I.6** |
| | -777 Skip to **I.4** | -777 Skip to **I.4** | -777 Skip to **I.4** | -777 Skip to **I.4** |
| | -888 Skip to **I.6** | -888 Skip to **I.6** | -888 Skip to **I.6** | -888 Skip to **I.6** |
| | -999 Skip to **I.6** | -999 Skip to **I.6** | -999 Skip to **I.6** | -999 Skip to **I.6** |

| | | | | |
|---|---|---|---|---|
| I.3 | 1 | 1 | 1 | 1 |
| | 2 | 2 | 2 | 2 |
| | 3 | 3 | 3 | 3 |
| | 4 | 4 | 4 | 4 |
| | 5 | 5 | 5 | 5 |
| | 6 | 6 | 6 | 6 |
| | -777 | -777 | -777 | -777 |
| | -888 | -888 | -888 | -888 |
| | -999 | -999 | -999 | -999 |
| I.4 | 1 | 1 | 1 | 1 |
| | 2 | 2 | 2 | 2 |
| | 3 | 3 | 3 | 3 |
| | 4 | 4 | 4 | 4 |
| | 5 | 5 | 5 | 5 |
| | -888 | -888 | -888 | -888 |
| | -999 | -999 | -999 | -999 |

| | | | | |
|---|---|---|---|---|
| I.5 | माह/Months ☐☐ 1<br>दिन/Days ☐☐ 2 | माह/Months ☐☐ 1<br>दिन/Days ☐☐ 2 | माह/Months ☐☐ 1<br>दिन/Days ☐☐ 2 | माह/Months ☐☐ 1<br>दिन/Days ☐☐ 2 |
| | -888 | -888 | -888 | -888 |
| | -999 | -999 | -999 | -999 |
| I.6 | रू./Rs. ☐☐☐☐ 1 | रू./Rs. ☐☐☐☐ 1 | रू./Rs. ☐☐☐☐ 1 | रू./Rs. ☐☐☐☐ 1 |
| | -888 | -888 | -888 | -888 |
| | -999 | -999 | -999 | -999 |

| जे:वर्तमान उपचार<br>**J: Current treatment** | प्रश्नावली कोड/<br>**Questionnaire code: I__I__I I__I__I I__I__I I__I__I__I** | | |
|---|---|---|---|
| अब,मै आपसे वर्तमान मे चल रहे उपचार के बारे मे कुछ प्रश्न पूछना चाहूँगा.<br>Now, I'd like to ask you some questions regarding your current treatment | | | |
| J.0 | आपने अपनी लैब जांच कब कराई?<br>When did you have your lab test done? | दिन/day माह/month वर्ष/year 1<br>\|__\|__\| \|__\|__\| \|__\|__\|__\|__\|<br>जवाब नही देना -888<br>Will not answer<br>पता नही -999<br>Does not know | |
| J.1 | आपको टी.बी. फेफडों में है या फेफडों के बाहर?अगर बाहर .तो कहाँ?<br>Is your TB in your lungs or outside your lungs? If outside, where?<br><br>INTERVIEWER: DO NOT PROMPT<br>सक्षत्कार्यकर्ता; कृपया जवाब पढ़ कर ना जो भी लागू होता है उस पर घेरा बनाये<br>*CIRCLE ALL THAT APPLY* | फेफडो मे 1<br>Lungs (Pulmonary)<br>फेफडो के बाहर 2<br>Outside lungs (Extra Pulmonary)<br>विवरण दे,कहाँ?<br>Specify where<br>जवाब नही देना -888<br>Will not say<br>पता नही -999<br>Does not know | |
| J.2 | आपने पहली गोली कब ली ?<br>When did you take your first pill?<br><br>Surveyor: Please see the patient's treatment card if available.<br>सर्वेक्षक: कृपया मरीज़ का जाच पत्र देखे, अगर उपलब्ध हो | दिन/day माह/month वर्ष/year 1<br>\|__\|__\| \|__\|__\| \|__\|__\|__\|__\|<br>जवाब नही देना -888<br>Will not say<br>पता नही -999<br>Does not know | |
| J.3 | क्या आप सी पी या आई पी के मरीज हो?<br><br>Are you currently a CP or an IP patient? | आई पी<br>IP 1<br>सी पी<br>CP 2<br>जवाब नहीं देना -888<br>Will not say<br>पता नहीं -999<br>Does not know | |

## जे:वर्तमान उपचार
## J: Current treatment

**प्रश्नावली कोड/**
**Questionnaire code: |__|__| |__|__| |__|__| |__|__|__|**

| | | | |
|---|---|---|---|
| J.4 | **जिस डॉट्स केंद्र से आपका इलाज चल रहा है उसे कौन चलाता है?(कृपया उत्तरदाता को उत्तर देने के लिए प्रेरित करे)**<br><br>Who runs your current DOTS center? (PLEASE PROMPT THE POSSIBLE ANSWERS) | **आपरेशन आशा**<br>Operation ASHA | 1 |
| | | **सरकारी डिस्पेंसरी**<br>Government dispensary | 2 |
| | | **अन्य सरकारी केंद्र पर**<br>Other government run center | 3 |
| | | **एन.जी.ओ.संचालित केंद्र**<br>NGO run center | 4 |
| | | **निजी केंद्र**<br>Private center | 5 |
| | | **अन्य(विवरण दे)**<br>Other (Specify) | -777 |
| | | **जवाब नहीं देना**<br>Will not say | -888 |
| | | **पता नहीं**<br>Does not know | -999 |
| J.5 | **आपका डॉट्स केंद्र आपके घर के सबसे नज़दीक है या कार्यस्थल के?**<br>Is your DOTS center nearest to your house or your work? | **घर**<br>House | 1 |
| | | **कार्यस्थल**<br>Work | 2 |
| | | **कोई नहीं**<br>Neither | 3 |
| | | **जवाब नहीं देना**<br>Will not say | -888 |
| | | **पता नहीं**<br>Does not know | -999 |
| J.6 | **आप सामान्यतयः डॉट्स केंद्र कैसे जाते है और कैसे वापिस आते है?**<br>How do you usually go to the DOTS center and come back from there?<br><br>**उन सभी पर घेरा बांये जो लागू होते है.**<br>CIRCLE ALL THAT APPLY | **पैदल**<br>Walk | 1 |
| | | **साइकिल**<br>Bicycle | 2 |
| | | **मोटर बाइक**<br>Motor bike | 3 |
| | | **कार**<br>Car | 4 |
| | | **बस**<br>Bus | 5 |
| | | **ऑटो रिक्शा**<br>Auto rickshaw | 6 |
| | | **साइकिल रिक्शा**<br>Bicycle rickshaw | 7 |
| | | **कोई और उन्हे मेरे लिए लाता है**<br>Someone else gets them for me | 8 |
| | | **अन्य(विवरण दे)**<br>Other. Specify: | -777 |
| | | **जवाब नहीं देना**<br>Will not answer | -888 |

| | | | | |
|---|---|---|---|---|
| J.7 | **डॉट्स केंद्र तक जाने में कितना समय लगता है (एक तरफ से )?**<br>How much time does it take you to go to the center (one way)? | **मिनट**/Minutes [ ][ ][ ] | 1 | |
| | | **जवाब नहीं देना**<br>Will not answer | -888 | |
| | | **पता नहीं**<br>Does not know | -999 | |
| J.8 | **आपको एक सप्ताह में कितनी बार डॉट्स केंद्र जाना पड़ता है?**<br>How many times per week do you have to go to the center? | **संख्या** [ ][ ]<br>Number of times | 1 | |
| | | **जवाब नहीं देना**<br>Will not answer | -888 | |
| | | **पता नहीं**<br>Does not know | -999 | |
| J.9 | **सप्ताह के किन दिनों पर केंद्र सामान्यतयः खुला होता है?**<br>Which days of the week is the center usually open?<br><br>**उन सब पर घेरा बनाये जो लागू होते हैं.**<br>*CIRCLE ALL THAT APPLIES* | सोमवार<br>Monday | 1 | |
| | | **मंगलवार**<br>Tuesday | 2 | |
| | | **बुधवार**<br>Wednesday | 3 | |
| | | **ब्रस्पतिवार**<br>Thursday | 4 | |
| | | **शुक्रवार**<br>Friday | 5 | |
| | | **शनिवार**<br>Saturday | 6 | |
| | | **रविवार**<br>Sunday | 7 | |
| | | **हर सप्ताह समय बदलता रहता है.**<br>It changes from one week to another | 8 | |
| | | **जवाब नहीं देना**<br>Will not answer | -888 | |
| | | **पता नहीं**<br>Does not know | -999 | |
| J.10 | **सामान्य दिन पर,केंद्र कितने बजे खुल जाता है?**<br>On a usual opening day, at what time does the center usually open?<br>*सर्वेक्षक: 24 घंटा समय स्वरुप का उपयोग करें*<br>*Survevor: Use 24 hr time format* | **कोई निश्चित समय नहीं**<br>It varies a lot | 1 | |
| | | **घंटे**/Hour [ ][ ] **मिनट**/Minute [ ][ ] | 2 | |
| | | **जवाब नहीं देना**<br>Will not answer | -888 | |
| | | **पता नहीं**<br>Does not know | -999 | |

| | | | |
|---|---|---|---|
| J.11 | **एक सामान्य दिन पर,केंद्र कितने बजे बंद होता है?**<br>On a usual opening day, at what time does the center usually close?<br>*सर्वेक्षक: 24 घंटा समय स्वरूप का उपयोग करें*<br>*Surveyor: Use 24 hr time format* | **कोई निश्चित समय नही**<br>It varies a lot 1 | |
| | | **घंटे**/Hour [ ][ ] **मिनट**/Minute [ ][ ] 2 | |
| | | **जवाब नहीं देना**<br>Will not answer -888 | |
| | | **पता नहीं**<br>Does not know -999 | |
| J.12 | **आप केंद्र सामान्यतयः किस समय पर जाते**<br>At what time in the day do you usually go to the center?<br>**उन सब पर घेरा बनाये जो**<br>*CIRCLE ALL THAT APPLIES* | **प्रातः७ बजे से प्रातः १० बजे तक**<br>7am - 10am 1 | |
| | | **प्रातः १० बजे से दोपहर १ बजे तक**<br>10am - 1pm 2 | |
| | | **दोपहर १ बजे से सांय ४ बजे तक**<br>1pm - 4pm 3 | |
| | | **सांय ४ बजे से सांय ७ बजे तक**<br>4pm - 7pm 4 | |
| | | **सांय ७ बजे के बाद**<br>Later than 7pm 5 | |
| | | **जवाब नहीं देना**<br>Will not answer -888 | |
| | | **पता नहीं**<br>Does not know -999 | |
| J.12.1 | **जब आप दवा लेने जाते है/थे तो क्य कभी अपको केंद्र बन्द मिला?**<br>Have you ever found that the center was closed when you went to take medicine? | **हाँ**<br>Yes 1 | |
| | | **नहीं**<br>No 2 | Skip to J.13 |
| | | **जवाब नहीं देना**<br>Will not say -888 | Skip to J.13 |
| | | **पता नहीं**<br>Does not know -999 | Skip to J.13 |
| J.12.2 | **अगर हा तो कितनी बार?**<br>If yes, then how many times? | [ ][ ] 1 | |
| | | **जवाब नहीं देना**<br>Will not say -888 | |
| | | **पता नहीं**<br>Does not know -999 | |
| J.13 | **आपको सामान्यतयःअपनी गोलियाँ लेने के लिए केंद्र में कितनी देर लगती है?**<br>How long do you usually have to wait in the center before getting your pills? | **कोई निश्चित समय नहीं**<br>It varies a lot 1 | |
| | | **घंटे**/Hour [ ][ ] **मिनट**/Minute [ ][ ] 2 | |
| | | **जवाब नहीं देना**<br>Will not answer -888 | |
| | | **पता नहीं**<br>Does not know -999 | |
| J.14 | **क्या डॉट्स केंद्र जाना सुविधाजनक है?**<br>Do you find going to your DOTS center convenient? | **हाँ**<br>Yes 1 | Skip to **J.16** |
| | | **नहीं**<br>No 2 | |
| | | **जवाब नहीं देना**<br>Will not say -888 | Skip to **J.16** |
| | | **पता नहीं**<br>Does not know -999 | Skip to **J.16** |

| | | | | |
|---|---|---|---|---|
| J.15 | **डॉट्स केंद्र जाना असुविधाजनक क्यों है ? (उन सब पर घेरा बनाये जो लगो होते हैं)**<br>Why do you find going to the DOTS center inconvenient? (Circle all that apply) | **केन्द्र जाने के लिए मैं बहुत कमज़ोर हूँ.**<br>Feel too weak to go to the center | 1 | |
| | | **केन्द्र बहुत दूर है**<br>Distance is too far | 2 | |
| | | **यातायात बहुत महंगा है**<br>Transportation is too expensive | 3 | |
| | | **स्वास्थ्य कर्मचारी केन्द्र में उपलभ नहीं होते**<br>Health workers not present during opening hours | 4 | |
| | | **काम कर रहा होता हू केन्द्र के खुले होने के समय**<br>Work during center's opening hours | 5 | |
| | | **बार बार जाना पड़ता है**<br>I have to go there too often | 6 | |
| | | **केन्द्र में बहुत देर तक प्रतीक्षा करनी पड़ती है**<br>I have to wait too long in the center | 7 | |
| | | **केन्द्र के खुले रहने का समय असुविधाजनक है.**<br>Opening time is not convenient | 8 | |
| | | **अन्य(विवरण दे)**<br>Other (Specify) | -777 | |
| | | **जवाब नहीं देना**<br>Will not say | -888 | |
| | | **पता नहीं**<br>Does not know | -999 | |
| J.16 | **डॉट्स केन्द्र में आप दवाई लेने के आलावा और क्या करते है?**<br>When you are at the DOTS center, what do you do in addition to taking your pills?<br>**जो भी लागू होता है उस पर घेरा बनाये**<br>*CIRCLE ALL THAT APPLY* | **कुछ नहीं**<br>Nothing | 1 | |
| | | **उपचार सम्बन्धी सलाह लेना**<br>Seek advice regarding the treatment | 2 | |
| | | **दूसरी दवाइयां मांगना**<br>Ask for other medicine / painkillers | 3 | |
| | | **बातचीत करना**<br>Have a chat | 4 | |
| | | **अन्य(विवरण दे)**<br>Other. Specify: | -777 | |
| | | **जवाब नहीं देना**<br>Will not say | -888 | |
| | | **पता नहीं**<br>Does not know | -999 | |

| | | | |
|---|---|---|---|
| J.17 | आप सामान्यतयः केन्द्र में किससे बातचीत करते है?<br>Whom do you usually interact with at the center?<br><br>जो भी लागू होता है उस पर घेरा बनाये<br>*CIRCLE ALL THAT APPLY* | डॉक्टर<br>Doctor | 1 |
| | | ए.एन.एम्.<br>ANM | 2 |
| | | ऑपेरशन आशा सलाहकार<br>Operation ASHA's counselor | 3 |
| | | अन्य स्वास्थ्य कर्मचारी<br>Other health worker | 4 |
| | | आपरेशन आशा के डॉट्स कार्यकर्ता<br>Operation ASHA's DOTS provider | 5 |
| | | अन्य मरीज<br>Other patients | 6 |
| | | कोई नहीं<br>No one | 7 |
| | | अन्य(विवरण दे)<br>Other. Specify: | -777 |
| | | जवाब नहीं देना<br>Will not say | -888 |
| | | पता नहा<br>Does not know | -999 |
| J.18 | क्या केन्द्र पर कोई कर्मचारी आपके वाह जाने और दवाइयाँ लेने का लेखा जोखा रखता है?<br>Do you know if someone at the center keeps track of the days you come to the center and take your pills? | हाँ,कोई लिखता है<br>Yes, someone does | 1 |
| | | नहीं,कोई नहीं लिखता<br>No, nobody does | 2 |
| | | जवाब नहा देना<br>Will not say | -888 |
| | | पता नहीं<br>Does not know | -999 |
| J.19 | क्या आप अपने डॉट्स केन्द्र के डॉट्स प्रदानकर्ता, [नाम] को जानते है?<br>Do you know *[NAME]* , the DOTS provider for this center?<br>सर्वेक्षक:कृपया डॉट्स प्रदानकर्ता के नाम का उलेख करें.<br>*SURVEYOR: please mention the name of the DOTS provider to the patient* | हाँ<br>Yes | 1 |
| | | नहीं<br>No | 2 |
| | | जवाब नहीं देना<br>Will not answer | -888 |

| | | | |
|---|---|---|---|
| J.20 | क्या आपने कभी अपनी स्थिति के बारे में (नाम)डॉट्स प्रदानकर्ता से बात की है?<br>Have you ever talked about your condition with *[NAME]* , the DOTS provider for this center? | हाँ,कई बार<br>Yes often | 1 |
| | | कभी-कभी<br>Yes sometimes | 2 |
| | | शायद ही कभी<br>Rarely | 3 |
| | | कभी नहीं<br>Never | 4 |
| | | जवाब नहीं देना<br>Will not answer | -888 |
| | | पता नहीं<br>Does not know | -999 |
| J.21 | क्या आप अपने डॉट्स केन्द्र के टी.बी.एच.वी. [नाम] को जानते है?<br>Do you know *[NAME]* , the TB Health Visitor for this community?<br><br>सर्वेक्षक:क्रप[या टी. बी.एच.वी.के नाम का उल्लेख करें.<br>*SURVEYOR: please mention the name of the TB Health Visitor to the patient* | हाँ<br>Yes | 1 |
| | | नहीं<br>No | 2 |
| | | जवाब नहीं देना<br>Will not answer | -888 |
| J.22 | क्या आपने कभी अपनी स्थिति के बारे में (नाम), टी.बी.एच.वी. से बात की है?<br>Have you ever talked about your condition with *[NAME]* , the TB Health Visitor for this community? | हाँ,कई बार<br>Yes often | 1 |
| | | हाँ,कुछेक बार<br>Yes sometimes | 2 |
| | | शायद ही कभी<br>Rarely | 3 |
| | | कभी नहीं<br>Never | 4 |
| | | जवाब नहीं देना<br>Will not answer | -888 |
| | | पता नहीं<br>Does not know | -999 |
| J.23 | क्या आप [ नाम] , वरिष्ट टी. बी सुपर्व्यसर को जानते हैं ?<br><br>Do you know *[NAME]* , the Senior TB Supervisor ?<br>सर्वेक्षक:क्रप[या वरिष्ट टी. बी सुपर्व्यसर के नाम का उल्लेख करें.<br>*SURVEYOR: please mention the name of the STS to the patient* | हाँ<br>Yes | 1 |
| | | नहीं<br>No | 2 |
| | | जवाब नहीं देना<br>Will not answer | -888 |

| | | | |
|---|---|---|---|
| J.24 | क्या आपने कभी अपनी स्थिति के बारे में (नाम), वरिष्ट टी.बी सुपर्व्यर्सर से बात की है?<br><br>Have you ever talked about your condition with *[NAME]* , the Senior TB Supervisor ? | हाँ,कई बार<br>Yes often 1<br>हाँ,कुछेक बार<br>Yes sometimes 2<br>शायद ही कभी<br>Rarely 3<br>कभी नहीं<br>Never 4<br>जवाब नहीं देना<br>Will not answer -888<br>पता नहा<br>Does not know -999 | |
| J.25 | क्या आप ए.एन.एम्. , [नाम], को जानती हैं?<br>Do you know *[NAME]* , the ANM?<br><br>सर्वेक्षक:क्रप[या ए.एन.एम्.के नाम का<br>*SURVEYOR: please mention the name of the ANM to the patient* | हाँ<br>Yes 1<br>नहीं<br>No 2<br>जवाब नहीं देना<br>Will not answer -888 | |
| J.26 | क्या आपने कभी अपनी स्वास्थ्य स्थिति के बारे में (नाम)ए.एन.एम्. से बात की है?<br>Have you ever talked about your condition with *[NAME]* , the ANM? | हाँ,कई बार<br>Yes often 1<br>हाँ,कुछेक बार<br>Yes sometimes 2<br>शायद ही कभी<br>Rarely 3<br>कभी नहीं<br>Never 4<br>जवाब नहीं देना<br>Will not answer -888<br>पता नहीं<br>Does not know -999 | |

| | | | |
|---|---|---|---|
| J.27 | क्या आपके टी.बी.उपचार मे कुछ दुश प्रभाव/गौण प्रभाव भी हो रहे है?<br>Does your TB treatment cause side effects? | हाँ,कई बार<br>Yes often 1 | |
| | | हाँ,कुछेक बार<br>Yes sometimes 2 | |
| | | शायद ही कभी<br>Rarely 3 | |
| | | कभी नहीं<br>Never 4 | Skip to **K.0** |
| | | जवाब नहीं देना<br>Will not answer -888 | Skip to **K.0** |
| | | पता नहा<br>Does not know -999 | Skip to **K.0** |
| J.28 | किस प्रकार के दुश प्रभाव हो रहे है?<br>What type of secondary effects?<br><br>उन सब पर घेरा बनाए जो लागू होते है)<br>Circle all that apply | ददौरे/पीलिया<br>Skin rash/Jaundice 1 | |
| | | पेट खराब/पेट दर्द<br>Upset stomach/Abdominal pain 2 | |
| | | धुँधला दिखना/रंग न पहचान पाना<br>Blurry vision or color-blindness 3 | |
| | | गहरे रंग का मूत्र आना<br>Dark urine 4 | |
| | | बुखार<br>Fever 5 | |
| | | उल्टी आना/ उबकाई<br>Nausea/Vomiting 6 | |
| | | अन्य(विवरण दे)<br>Other. Specify: -777 | |
| | | जवाब नहो देना<br>Will not answer -888 | |
| | | पता नहीं<br>Does not know -999 | |
| J.29 | क्या आप उपचार के दुश प्रभावों को रोकने के लिए गोलियाँ लेते है?<br>Do you take pills to limit secondary effects? | हाँ<br>Yes 1 | |
| | | नहीं<br>No 2 | |
| | | जवाब नहीं देना<br>Will not answer -888 | |
| | | पता नहीं<br>Does not know -999 | |

## क:परामर्शदाता से बातचीत
## K: Interaction with the counselor

Questionnaire code: |__|__| |__|__| |__|__| |__|__|__|

*Surveyor: Please take patient's response only for this section.*
*सर्वेक्षक: सिर्फ़ मरीज़ का जवाब लेना है*

**अब ,मैं आपसे आपके टी.बी.उपचार केंद्र में कार्यरत ऑपरेशन आशा सलाहकार से हुए वार्तलाप से सम्बंधित प्रश्न पूछना चाहूंगी**
*Now, I'd like to ask you some questions regarding your interactions with the Operation ASHA counselor working in your TB treatment center*

| | | | | |
|---|---|---|---|---|
| K.0 | **आप ओ.ए. सलाहकार से पहली बार कहाँ मिले?**<br>Where did you meet OA counselor for the first time?<br><br>**सर्वेक्षक:कृपया मरीज़ के सामने ऑपरेशन आशा सलाहकार के नाम का उल्लेख करे**<br><br>*SURVEYOR: please mention the name of the Operation ASHA counselor to the patient* | **आपके घर में**<br>In your house | 1 | |
| | | **जांच केंद्र पर.**<br>At the detection center | 2 | |
| | | **डॉट्स केंद्र पर**<br>At the DOTS center | 3 | |
| | | **दोस्त / रिश्तेदार के घर पर**<br>At a friend's/relative's house | 4 | |
| | | **कभी ओ.ए. सलाहकार से नहीं मिले**<br>Never met OP Asha Counselor | 5 | |
| | | **अन्य (विवरण दे)** ____________<br>Other (specify) | -777 | |
| | | **जवाब नहीं देना**<br>Will not answer | -888 | |
| | | **पता नहीं**<br>Does not know | -999 | |
| K.1 | **आप ऑपरेशन आशा सलाहकार को कितनी बार देखते है?**<br>How often do you see the Operation ASHA counselor? | [ ][ ] | | |
| | | **प्रति दिन**<br>Per day | 1 | |
| | | **प्रति सप्ताह**<br>Per Week | 2 | |
| | | **प्रति महीना**<br>Per Month | 3 | |
| | | **कभी नहीं**<br>Never | 4 | |
| | | **अन्य(विवरण दे)** ____________<br>Other (specify) | -777 | |
| | | **जवाब नहीं देना**<br>Will not answer | -888 | |
| | | **पता नहीं**<br>Does not know | -999 | |
| K.2 | **जब आप डॉट्स केद्र जाते है तो कितनी बार ऑपरेशन आशा सलाहकार से मिलते है?**<br>When you visit the DOTS center, how often do you meet the Operation ASHA counselor in the center? | [ ][ ] | | |
| | | **प्रति दिन**<br>Per day | 1 | |
| | | **प्रति सप्ताह**<br>Per Week | 2 | |
| | | **प्रति महीना**<br>Per Month | 3 | |
| | | **कभी नहीं**<br>Never | 4 | |
| | | **अन्य(विवरण दे)** ____________<br>Other (specify) | -777 | |
| | | **जवाब नहीं देना**<br>Will not answer | -888 | |
| | | **पता नहीं**<br>Does not know | -999 | |

## क:परामर्शदाता से बातचीत
## K: Interaction with the counselor

Questionnaire code: |__|__| |__|__| |__|__| |__|__|__|

| | | | | |
|---|---|---|---|---|
| K.3 | क्या तुमने कभी ऑपरेशन आशा सलाहकार को डॉट्स केंद्र के अलावा किसी और जगह देखा है? यदि हां, तो कितनी बार?<br><br>Have you ever seen the Operation ASHA counselor anywhere outside of the center? If so, how often? | ☐☐ | | |
| | | प्रति दिन<br>Per day | 1 | |
| | | प्रति सप्ताह<br>Per Week | 2 | |
| | | प्रति महीना<br>Per Month | 3 | |
| | | कभी नहीं<br>Never | 4 | |
| | | अन्य(विवरण दे) ______<br>Other (specify) | -777 | |
| | | जवाब नहीं देना<br>Will not answer | -888 | |
| | | पता नहीं<br>Does not know | -999 | |
| K.4 | क्या ऑपरेशन आशा सलाहकार ने कभी अपकी जगह का दौरा किया? यदि हां, तो कितनी बार?<br>Has the Operation ASHA counselor ever visited your place? If so, how often? | ☐☐ | | |
| | | प्रति दिन<br>Per day | 1 | |
| | | प्रति सप्ताह<br>Per Week | 2 | |
| | | प्रति महीना<br>Per Month | 3 | |
| | | कभी नही<br>Never | 4 | |
| | | अन्य(विवरण दे) ______<br>Other (specify) | -777 | |
| | | जवाब नहीं देना<br>Will not answer | -888 | |
| | | पता नहीं<br>Does not know | -999 | |
| K.5 | क्या ऑपरेशन आशा सलाहकार आपकी दोस्त,पडोसी या रिश्तेदार में से कोई है?<br>Is OA counselor a friend or relative or neighbour?<br><br>जो भी लागू होता है उस पर घेरा बनाये<br>*CIRCLE ALL THAT APPLY* | दोस्त<br>Friend | 1 | |
| | | रिश्तेदार<br>Relative | 2 | |
| | | पडोसी<br>Neighbor | 3 | |
| | | कोई नहीं<br>Neither of them | 4 | |
| | | जवाब नही देना<br>Will not answer | -888 | |
| | | पता नहीं<br>Does not know | -999 | |

## क:परामर्शदाता से बातचीत
## K: Interaction with the counselor

Questionnaire code: |__|__| |__|__| |__|__| |__|__|__|

| | | | |
|---|---|---|---|
| K.6 | क्या आप ऑपरेशन आशा सलाहकार से अपनी सामान्य स्वास्थ्य स्थिति के बारे में बात करने में भरोसा रखते हो?<br>Do you feel confident enough to talk with the Operation ASHA counselor about your general health condition? | हाँ Yes 1<br>नहीं No 2<br>जवाब नहीं देना Will not answer -888<br>पता नही Does not know -999 | |
| K.7 | क्या आप ऑपरेशन आशा सलाहकार से अपने परिवार के सामान्य स्वास्थ्य स्थिति के बारे में बात करने में भरोसा रखते हो?<br>Do you feel confident enough to talk with the Operation ASHA counselor about your family's health condition? | हाँ Yes 1<br>नही No 2<br>जवाब नही देना Will not answer -888<br>पता नही Does not know -999 | |
| K.8 | क्या ऑपरेशन आशा सलाहकार आपको टी.बी. सम्बन्धी सलाह देती है?<br>Does the Operation ASHA counselor give you advice related to TB? | हां,बहृत Yes a lot 1<br>हां,थोड़ी बहृत Yes a little 2<br>नहीं No 3<br>जवाब नही देना Will not answer -888<br>पता नहीं Does not know -999 | <br><br>Skip to **K.10**<br>Skip to **K.10**<br>Skip to **K.10** |
| K.9 | किस तरह की सलाह देती है ?<br>What kind of advice?<br>(उन सब पर घेरा बनाये जो लागू होते है)<br>CIRCLE ALL THAT APPLY | उपचार सम्बन्धी Advice on treatment 1<br>संक्रमण सम्बन्धी Advice on contamination 2<br>पक्षीयप्रभाव/दुष्प्रभाव सम्बन्धी Advice on side effects 3<br>पहचान/ अभिज्ञान सम्बन्धी Advice on detection 4<br>अन्य(विवरण दे) ____ Other (specify) -777<br>जवाब नहीं देना Will not answer -888<br>पता नहीं Does not know -999 | |

# क:परामर्शदाता से बातचीत
# K: Interaction with the counselor

Questionnaire code: |__|__| |__|__| |__|__| |__|__|__|

| | | | | |
|---|---|---|---|---|
| K.10 | वे कौन से अन्य लोग है जो आपको टी.बी. सम्बन्धी जानकारी देते है?<br>Who else gives you advice related to TB?<br><br>(उन सब पर घेरा बनाये जो लागू होते है)<br>CIRCLE ALL THAT APPLY | कोई नहीं<br>Nobody | 1 | Skip to **K.13** |
| | | निजी डॉक्टर<br>A private doctor | 2 | |
| | | भोपा<br>A bhopa | 3 | |
| | | ए.एन.एम्.<br>ANM | 5 | |
| | | आंगनवाड़ी<br>Anganwadi | 6 | |
| | | अन्य सरकरी स्वास्थ्य कर्मचारी<br>Other government health worker | 7 | |
| | | मेरे अभिभावक/बच्चे/पति/पत्नी<br>My spouse/parents/children | 8 | |
| | | दोस्त/रिश्तेदार<br>A friend/relative | 9 | |
| | | अन्य(विवरण दे) ____<br>Other (specify) | -777 | |
| | | जवाब नहीं देना<br>Will not answer | -888 | Skip to **K.13** |
| | | पता नहीं<br>Does not know | -999 | Skip to **K.13** |
| K.11 | वे लगभग कितनी बार आपको सलाह देते है?<br>How often do they give you advice? | सप्ताह में कम से कम एक बार<br>At least every week | 1 | |
| | | एक सप्ताह में एक बार से लेकर एक महीने में एक बार<br>Between once a week and once a month | 2 | |
| | | एक महीने में एक बार से लेकर एक वर्ष में<br>Between once a month and once a year | 3 | |
| | | एक वर्ष में एक बार से भी कम<br>Less than once a year | 4 | |
| | | जवाब नहीं देना<br>Will not answer | -888 | |
| | | पता नहीं<br>Does not know | -999 | |
| K.12 | किस तरह की सलाह देते है ?<br>What kind of advice?<br><br>(उन सब पर घेरा बनाये जो लागू होते है)<br>CIRCLE ALL THAT APPLY | उपचार सम्बन्धी<br>Advice on treatment | 1 | |
| | | संक्रमण सम्बन्धी<br>Advice on contamination | 2 | |
| | | पक्षीयप्रभाव/दुष्प्रभाव सम्बन्धी<br>Advice on side effects | 3 | |
| | | पहचान/ अभिज्ञान सम्बन्धी<br>Advice on detection | 4 | |
| | | अन्य(विवरण दे) ____<br>Other (specify) | -777 | |
| | | जवाब नहीं देना<br>Will not answer | -888 | |
| | | पता नहीं<br>Does not know | -999 | |

## क:परामर्शदाता से बातचीत
## K: Interaction with the counselor

Questionnaire code: |__|__| |__|__| |__|__| |__|__|__|

| | | | |
|---|---|---|---|
| K.13 | **क्या ऑपरेशन आशा सलाहकार आपको उपचार के दौरान सहायता करती है?**<br><br>Does OA counselor support you during your treatment? | **हॉ,अत्यधिक** 1<br>Yes a lot<br>**हॉ,थोड़ी बहुत** 2<br>Yes a little<br>**नहीं,कुछ खास नहीं** 3<br>No, not much<br>**नहीं,बिलकुल भी नहीं** 4<br>No, not at all<br>**जवाब नहीं देना** -888<br>Will not answer<br>**पता नहीं** -999<br>Does not know | |
| K.14 | **क्या ऑपरेशन आशा सलाहकार ने आपको आपके बीमार होने के दौरान आपको डॉक्टर /अस्पताल ले जाने की कभी स्वयं व्यवस्था की?**<br>If you have been sick, has the OA counselor arranged to personally take you to the doctor/ hospital? | **हाँ, हर बार जब भी मैं बीमार हुआ हूँ.** 1<br>Yes every time I've been sick<br>**हॉ,कभी -कभी** 2<br>Yes sometimes<br>**नहीं,बहुत अधिक बार नहीं** 3<br>No, not much<br>**नहीं,कभी नहीं** 4<br>No, never<br>**जवाब नहीं देना** -888<br>Will not answer<br>**पता नहीं** -999<br>Does not know | |
| K.15 | **क्या ऑपरेशन आशा सलाहकार ने कभी आपके खाने,यातायात के लिए व्यय किये या कोई अन्य आर्थिक सहायता की हो?**<br>Has the OA counselor paid for your meals, transport or provided any other financial support? | **हाँ,हर बार जब भी में केंद्र आता हूँ** 1<br>Yes every time I come to the center<br>**हॉ,कभी -कभी** 2<br>Yes sometimes<br>**नहीं,कुछ खास नहीं** 3<br>No, not much<br>**नहीं,कभी नहीं** 4<br>No, never<br>**जवाब नही देना** -888<br>Will not answer<br>**पता नहीं** -999<br>Does not know | |

 Questionnaire code: |__|__| |__|__| |__|__| |__|__|__|

| | | | |
|---|---|---|---|
| K.16 | क्या ऑपरेशन आशा सलाहकार ने तुम्हें एक सप्ताह या लंबी अवधि के लिए एक साथ दवाई दी?<br>Has the OA counselor given you medicines for a week or longer duration, together? | हाँ,कई बार<br>Yes, very often | 1 |
| | | हां,कभी-कभी<br>Yes sometimes | 2 |
| | | हाँ एक बार,<br>Yes, once | 3 |
| | | नहीं,बहुत बार नहीं<br>No, not much | 4 |
| | | नहीं,कभी नहीं<br>No, never | 5 |
| | | जवाब नहीं देना<br>Will not answer | -888 |
| | | पता नहीं<br>Does not know | -999 |
| K.17 | क्या ऑपरेशन आशा सलाहकार आपको आगाह करती हैं जब आप दवाइयां नहीं लेते हैं?<br>Does OA counselor warn you when you don't take your pills? | हाँ, हर बार जब मैं एक खुराक भी न लूं.<br>Yes every time I miss a pill | 1 |
| | | हां,कभी -कभी<br>Yes sometimes | 2 |
| | | नहीं,कुछ खास नहीं<br>No, not much | 3 |
| | | नहीं,कभी नहीं<br>No, never | 4 |
| | | मै अपनी दवाई लेना कभी नहीं भूला<br>I have never missed my pills | 5 |
| | | जवाब नहीं देना<br>Will not answer | -888 |
| | | पता नहीं<br>Does not know | -999 |
| K.18 | ऑपरेशन आशा सलाहकार कितनी बार आपके घर दवा देने आते है?<br>How often does the counselor deliver medicine to your home? | कभी कभी<br>Sometimes | 1 |
| | | हमेशा<br>Always | 2 |
| | | कभी नहीं<br>Never | 3 |
| | | जवाब नहीं देना<br>Will not answer | -888 |
| | | पता नहीं<br>Does not know | -999 |

## क:परामर्शदाता से बातचीत
## K: Interaction with the counselor

Questionnaire code: |__|__| |__|__| |__|__| |__|__|__|

| | | | |
|---|---|---|---|
| K.19 | पिछले कुछ सप्ताह में ,क्या आपने टी.बी.से सम्बंधित कोई प्रश्न या शंका ऑपरेशन आशा सलाहकार से पूछी है?<br>In the past few weeks, did you discuss any doubt or question related to TB with OA counselor? | हॉ कई बार<br>Yes several times 1 | |
| | | हॉ कुछ बार<br>Yes a few times 2 | |
| | | नहीं<br>No 3 | |
| | | जवाब नहीं देना<br>Will not answer -888 | |
| | | पता नहीं<br>Does not know -999 | |
| K.20 | पिछले कुछ सप्ताहो में ,क्या आपने अपने खुद के सामान्य स्वास्थ्य या आपके परिवार के किसी सदस्य के स्वास्थ्य से सम्बंधित कोई प्रश्न या शंका ऑपरेशन आशा सलाहकार से पूछी है?<br>In the past few weeks, did you discuss any doubt or question related to your health in general or to the health of any of your family members with OA counselor? | हॉ,कई बार<br>Yes several times 1 | |
| | | हॉ,कुछ बार<br>Yes a few times 2 | |
| | | नहीं<br>No 3 | |
| | | जवाब नहीं देना<br>Will not answer -888 | |
| | | पता नहीं<br>Does not know -999 | |
| K.21 | क्या आप ऑपरेशन आशा सलाहकार से होने वाले अपने साक्षात्कार से संतुष्ट है?<br>Are you satisfied with your interaction with OA counselor? | हॉ,बहुत संतुष्ट<br>Yes, very satisfied 1 | **Skip to L.0** |
| | | हॉ,संतुष्ट<br>Yes, rather satisfied 2 | **Skip to L.0** |
| | | नहीं, कुछ असंतुष्ट<br>No, rather dissatisfied 3 | |
| | | नहीं,अत्यंत असंतुष्ट<br>No, very dissatisfied 4 | |
| | | जवाब नहीं देना<br>Will not answer -888 | **Skip to L.0** |
| | | पता नहीं<br>Does not know -999 | **Skip to L.0** |
| K.22 | आप क्यों असंतुष्ट है?<br>(उन सब पर घेरा बनाये जो लागू होते है)<br>Why are you dissatisfied?<br>(CIRCLE ALL THAT APPLY) | कभी उपलब्ध नही होती<br>Is never available 1 | |
| | | केंद्र पर कभी उपस्थित नहीं होती<br>Is never present at the center 2 | |
| | | अच्छी सेवा नही प्रदान करती<br>Does not provide good advice 3 | |
| | | मेरी स्वास्थ्य स्थिति के प्रति गंभीर नहीं लग<br>Does not seem to care about my condition 4 | |
| | | अन्य(विवरण दे)<br>Other (Please specify) -777 | |
| | | जवाब नहीं देना<br>Will not answer -888 | |
| | | पता नहीं<br>Does not know -999 | |

| ल: टी. बी सम्बंधित जानकारी<br>L: TB Knowledge | | प्रश्नावली कोड/<br>Questionnaire code: \|__\|__\| \|__\|__\| \|__\|__\| \|__\|__\|__\| | |
|---|---|---|---|
| L.0 | **क्या आपको टी बी के कुछ लक्षण पता है ?**<br>Do you know some symptoms of TB? | **हा** 1<br>Yes | |
| | | **नहीं** 2<br>No | Skip to **L.2** |
| | | **जवाब नहीं दिया** -888<br>Will not say | Skip to **L.2** |
| | | **मालूम नही** -999<br>Does not know | Skip to **L.2** |
| L.1 | **आप टी बी के कौन कौन से लक्षण जानतें है ?**<br>What symptoms of TB do you know?<br><br>*INTERVIEWER: DO NOT PROMPT*<br><br>**साक्षात्कारकर्ता : कृपया जवाब पढकर न सुनायें**<br><br>**जो भी लागू होता है उस पर घेरा बनाये**<br>*CIRCLE ALL THAT APPLY* | **वजन में कमी** 1<br>Loss of weight | |
| | | **उर्जा में कमी** 2<br>Loss of energy | |
| | | **भूख कम होना** 3<br>Poor appetite | |
| | | **बुखार** 4<br>Fever | |
| | | **खाँसी** 5<br>Cough | |
| | | **रात में पसीन होना** 6<br>Night sweats | |
| | | **अन्य 1. स्पष्ट करें** 7<br>Other 1. Specify: ……………………………………… | |
| | | **अन्य 2. स्पष्ट करें** 8<br>Other 2. Specify: ……………………………………… | |
| | | **अन्य 3. स्पष्ट करें** 9<br>Other 3. Specify: ……………………………………… | |
| | | **जवाब नहीं दिया** -888<br>Will not say | |
| | | **मालूम नही** -999<br>Does not know | |

## ल: टी. बी सम्बंधित जानकारी
## L: TB Knowledge

प्रश्नावली कोड/
Questionnaire code: |__|__| |__|__| |__|__| |__|__|__|

| | क्या आप टी बी के बारें में निम्न कथनों से सहमत है ?<br>Do you agree with the following statements about TB? | हाँ<br>Yes | नहीं<br>No | मालूम नहीं<br>Does not know | जवाब नही दिया<br>Will not say | |
|---|---|---|---|---|---|---|
| L.2 | टीबी एक संक्रामक रोग है<br>TB is a communicable disease | 1 | 2 | -999 | -888 | |
| L.3 | टी बी वंशानुगत है ।<br>TB is hereditary | 1 | 2 | -999 | -888 | |
| L.4 | टी बी जीवाणु की वजह से होती हैं ।<br>TB is caused by a germ | 1 | 2 | -999 | -888 | |
| L.5 | टी.बी खासने और छींकने से बूंदों द्वारा फैलता है<br>TB is spread by droplets through cough and sneeze | 1 | 2 | -999 | -888 | |
| L.6 | टी बी के इलाज के लिये कारगर दवायें उपलब्ध है ।<br>There are effective drugs to treat TB | 1 | 2 | -999 | -888 | |
| L.7 | टी. बी के इलाज के लिए दवाइयां छे से आठ महीने तक लेना चाहिए<br>Anti-TB drugs should be given for 6 to 8 months | 1 | 2 | -999 | -888 | |
| L.8 | टी.बी को ठीक करने वाली दवाई, एक या दो दिन तक छोड़ सकतें हें<br>Anti-TB drugs can be skipped for a day or two | 1 | 2 | -999 | -888 | |
| L.9 | टी.बी से मृत्यु हो सकती है<br>TB can cause death | 1 | 2 | -999 | -888 | |
| L.10 | अगर मरीज़ बेहतर महसूस करने लगता है तो टी.बी की दवाइयां रोकी जा सकता है<br>TB drugs should be discontinued if patient starts feeling better | 1 | 2 | -999 | -888 | |
| L.11 | एच. आई .वी के मरीज़ों को टी.बी होने का ज्यादा खतरा रहता हें<br>HIV patients have a higher chance of getting TB | 1 | 2 | -999 | -888 | |
| L.12 | दवाई ना लेने पर टी.बी का और संजीदा रूप से मरीज़ ग्रस्त हो सकती है.<br>Failure to take medicine can yield to more serious forms of TB | 1 | 2 | -999 | -888 | |

| ल: टी. बी सम्बंधित जानकारी<br>L: TB Knowledge | प्रश्नावली कोड/<br>Questionnaire code: \|__\|__\| \|__\|__\| \|__\|__\| \|__\|__\|__\| |
|---|---|

| | | | |
|---|---|---|---|
| L.13 | क्या आप मुझे बता सकते है कि कोई टी. बी का संक्रमण कैसे करता है?<br>Can you please tell me how one can contract TB?<br><br>*INTERVIEWER: DO NOT PROMPT CIRCLE ALL THAT APPLY*<br>*सक्षत्कार्यकर्ता; कृपया जवाब पढ़ कर ना सुनाए जो लागू हो, उन पर गोला लगाएँ* | जब कोई संक्रमित व्यक्ति खाँसता या छींकता है ।<br>When another infected person coughs or sneezes | 1 |
| | | संक्रमित व्यक्ति के बर्तन या बोतल इस्तेमाल करने से ।<br>By sharing dishes or bottles with an infected person | 2 |
| | | छोटे बच्चों को चूमने से<br>By kissing little children | 3 |
| | | किसी विशेष भोजन से<br>By eating certain food | 4 |
| | | बहुत समय तक खाना न खाने से<br>By going without food for a long time | 5 |
| | | शराब पीने से<br>By consuming alcohol | 6 |
| | | अन्य(स्पष्ट करें) ______<br>Other (specify) | -777 |
| | | जवाब नही दिया<br>Will not answer | -888 |
| | | मालूम नही<br>Does not know | -999 |
| L.14 | टी बी की पुष्टि होने के पहले क्या आप अलग कमरे में सोते थे?<br><br>Before you were diagnosed with TB, did you sleep in a separate room? | हाँ<br>Yes | 1 |
| | | नहीं<br>No | 2 |
| | | जवाब नही दिया<br>Will not answer | -888 |
| L.15 | टी बी की पुष्टि होने के बाद से क्या आप परिवार के दूसरे सदस्यों से अलग कमरे में सोते है ?<br>Have you been sleeping in another room separate from other household members since being diagnosed? | हाँ<br>Yes | 1 |
| | | नहीं<br>No | 2 |
| | | जवाब नही दिया<br>Will not answer | -888 |
| | | मालूम नही<br>Does not know | -999 |

प्रश्नावली कोड/
Questionnaire code: |__|__| |__|__| |__|__| |__|__|__|

| | | | | |
|---|---|---|---|---|
| L.16 | टी बी फैलने से रोकने के लिये क्या आप मुँह को हाथ से ढ़ँकते है? यदि हाँ तो कैसे ?<br><br>Do you cover your mouth to prevent the further spread of TB? If yes, how?<br><br>*INTERVIEWER: CIRCLE ALL THAT APPLY*<br>*सक्षत्कार्यकर्ता; जो लागू हो, उन पर गोला लगाएँ* | **हाँ, खाँसी आने पर मैं अपने हाथों से अपना मुँह ढँक लेता हूँ।**<br>Yes, I cover my mouth with my hand when coughing | 1 | |
| | | **हाँ, मैं अपने मुँह पर एक कपड़ा/रुमाल रखता हूँ।**<br>Yes, I cover my mouth with a cloth/tissue | 2 | |
| | | **हाँ, मै अपने हाथ धोलेता हूँ**<br>Yes, I wash my hands afterwards | 3 | |
| | | **नहीं**<br>No | 4 | |
| | | **नहीं, मुझे खासी नहीं आती**<br>I do not have a cough | 5 | |
| | | **अन्य( स्पष्ट कीजिए )**<br><br>Other (specify) | -777 | |
| | | **जवाब नहीं दिया**<br>Will not answer | -888 | |
| | | **नहीं पता**<br>Does not know | -999 | |
| L.17 | **क्या आपने किसी ऐसी समुदयिक रैली या सभा में भाग लिया है जहाँ स्वास्थ्यकर्मी टी बी के बारें में बात करते है ?**<br>Have you attended any community rallies / meetings where health providers have talked about TB? | **हाँ**<br>Yes | 1 | |
| | | **नहीं**<br>No | 2 | Skip to **L.19** |
| | | **जवाब नही दिया**<br>Will not answer | -888 | Skip to **L.19** |
| | | **मालूम नहीं।**<br>Does not know | -999 | Skip to **L.19** |
| L.18 | **आपने ऐसी रैली या सभा में कितनी बार भाग लिया है ?**<br>How many meetings / rallies have you attended?<br><br>**जो भी लागू होता है उस पर घेरा बनाये**<br>*CIRCLE ALL THAT APPLY* | \|__\|__\| **मीटिंग**<br>meetings | 1 | |
| | | \|__\|__\| **रेली**<br>rallies | 2 | |
| | | **जवाब नही दिया**<br>Will not answer | -888 | |
| | | **मालूम नहीं।**<br>Does not know | -999 | |
| L.19 | **क्या आप अपने घर परिवार से बाहर किसी ऐसे व्यक्ति को जानते है जिसे टी बी हो ?**<br><br>Do you know anyone outside of your household/ family infected with TB? | **हाँ**<br>Yes | 1 | |
| | | **नहीं**<br>No | 2 | Skip to **L.21** |
| | | **जवाब नही दिया**<br>Will not answer | -888 | Skip to **L.21** |
| | | **मालूम नहीं।**<br>Does not know | -999 | Skip to **L.21** |

प्रश्नावली कोड/
Questionnaire code: |__|__| |__|__| |__|__| |__|__|__|

| | | | |
|---|---|---|---|
| L.20 | **उनके टी बी संक्रमण के बारें में जानने के बाद क्या आपने उनके साथ बिताये जाने वाले समय में कोई बदलाव किया ?**<br><br>After learning about their TB infection did you change the amount of time you spent with them? | **नहीं मैं उतना ही समय बिताता हूँ।**<br>No, I spent the same amount of time | 1 |
| | | **हाँ मैं संक्रमण के पहले के मुकाबले ज़्यादा समय बिताता हूँ।**<br>Yes, I spent more time with them than before their infection | 2 |
| | | **हाँ मैं संक्रमण के पहले के मुकाबले कम समय बिताता हूँ।**<br>Yes, I spent less time with them than before their infection | 3 |
| | | **जवाब नही दिया**<br>Will not answer | -888 |
| | | **मालूम नहीं।**<br>Does not know | -999 |
| L.21 | **आपकी बीमारी का इलाज कैसे होगा?**<br>How do you think your illness can be cured?<br><br>*सक्षत्कायकता; कृपया जवाब पढ़ कर ना सुनाए जो लागू हो, उन पर गोला लगाएँ*<br><br>*( INTERVIEWER : Do NOT prompt the options; circle all that is applicable)* | **मुझे नही लगता मेरे टी.बी ( इस बीमारी ) का कभी इलाज होगा**<br>I don't think I will ever get cured from this disease | 1 |
| | | **अगर मे रोज पूजा करू**<br>If I pray everyday | 2 |
| | | **अगर मे अच्छा व्यवहार करू**<br>If I behave well | 3 |
| | | **अगर मे अपनी दवाई डॉट्स केद्र से सही तरह से लूँ**<br>If I take my medicines properly from the DOTS center | 4 |
| | | **अगर मुझे कोई निजी स्किक्षित डाक्टर से अच्छी दवाई मिल जाये**<br>If some qualified private doctor gives me good medicines | 5 |
| | | **अगर मे भोजन सही तरह से लू**<br>If I eat properly | 6 |
| | | **अगर मुझे भोपा/ तांत्रिक बाबा/ अन्य झाड फूँक बाबा से कोई तंत्र मिल जाएँ**<br>If the Bhopa/ tantrik/ magico-religious person can give me a charm | 7 |
| | | मैं बिमारी से सवस्थय हो गया हूं<br>I have been cured of my illness | 8 |
| | | **अन्य ( स्पष्ट कीजिए )**<br>Any other ;<br>Please specify ____________ | -777 |
| | | **जवाब नहीं दिया**<br>Will not answer | -888 |
| | | **नहीं पता**<br>Does not know | -999 |

## म.सामाजिक भागीदारी

## M: Social insertion

प्रश्नावली कोड/Questionnaire code: |__|__| |__|__| |__|__| |__|__|__|

**साक्षात्कारकर्ता:अब,अगले कुछ प्रश्न आपके अपने समुदाय से कैसे सम्बन्ध है,इस पर आधारित होंगे.शुरुआत क्लब,समितियों और समूहों में आपकी भागीदारी से करेंगे.**

INTERVIEWER: The next set of questions concern your relations to your community, starting with your participation to clubs, groups, and associations.

| | | | |
|---|---|---|---|
| M.0 | **क्या आप समूह,क्लब या समितियों में भाग लेते है ?**<br><br>Do you take part in groups, clubs, or associations?<br><br>*Examples: caste commities, neighborhood commities, religious affliations, savings and/ or credit groups*<br><br>***उदाहरण: जाति समूह, पड़ोस की समितियाँ,धार्मिक जुड़ाव, बचत/ ऋण समूह*** | **हा** Yes 1 | |
| | | **नहीं** No 2 | Skip to M.**2** |
| | | **जवाब नहीं दिया** Will not answer -888 | Skip to M.**2** |
| | | **मालूम नहीं** Does not know -999 | Skip to M.**2** |
| M.1 | **ये किस तरह के समूह है ?**<br><br>What type of groups are these?<br><br>**साक्षात्कारकर्ता : जो भी लागू हो सब पर गोला लगाये ।**<br><br>*INTERVIEWER: Circle all that apply* | **स्त्रियो के सगठन** Women's organization 1 | |
| | | **जाति समूह** Caste committee 2 | |
| | | **युवा क्लब** Youth club 3 | |
| | | **पड़ोस की सामितिया** Neighborhood committee 4 | |
| | | **राजनीतिक दल** Political party 5 | |
| | | **चर्च,मंदिर,मस्जिद, देवरा, पंथ या गुरु** Church, temple, mosque, devra, sect or guru 6 | |
| | | **सहकारी,बचत/ऋण/अतिसूक्ष्म ऋण/लाभ समितियाँ या बीमा समूह** Cooperative, savings / credit / microcredit / profit or insurance group 7 | |
| | | **स्कूल सामितिया** School committee 8 | |
| | | **NGO या NGO से जुड़ी गतिविधियाँ** NGO or activities with an NGO 9 | |
| | | **अन्य: (स्पष्ट करे)** ______ Other (specify) -777 | |
| | | **जवाब नहीं दिया** Will not answer -888 | |
| | | **मालूम नहीं** Does not know -999 | |

## म.सामाजिक भागीदारी

## M: Social insertion

प्रश्नावली कोड/Questionnaire code: |__|__| |__|__| |__|__| |__|__|__|

| | | | |
|---|---|---|---|
| M.2 | क्या आपके परिवार के अलावा ऐसे लोग है जिनके साथ आप नियमित गतिविधियों(जैसे बाज़ार जाना, बात-चीत ) में भाग लेते है।<br><br>Are there other groups of people (besides your family) with whom you share regular activities (like going to the market or talking)? | हा<br>Yes 1<br><br>नहीं<br>No 2<br><br>जवाब नहीं दिया<br>Will not answer -888<br><br>मालूम नहीं<br>Does not know -999 | |
| M.3 | क्या आपको लगता है कि आपको अपनी स्थिति छुपा कर रखनी चाहिये नहींतो लोग आप से भेद-भाव करेंगे<br><br>Do you feel you have to keep your condition secret otherwise people will discriminate you? | हा बिल्कुल<br>Yes, absolutely 1<br><br>हाँ थोड़ा बहत<br>Yes, somewhat 2<br><br>नहीं बिल्कुल नहीं<br>No, not at all 3<br><br>जवाब नहीं दिया<br>Will not answer -888<br><br>मालूम नहीं<br>Does not know -999 | |
| M.4 | क्या आपके पड़ोसी आपकी बीमारी के बारें में जानते है ?<br><br>Do your neighbors know about your disease? | हाँ, उनमें से ज़्यादातर<br>Yes, most of them 1<br><br>हा, उनमे से कुछ<br>Yes, some of them 2<br><br>नहीं उनमें से कोई नही<br>No, none of them 3<br><br>जवाब नहीं दिया<br>Will not answer -888<br><br>मालूम नहीं<br>Does not know -999 | |
| M.5 | क्या आपके सहकर्मी आपकी बीमारी के बारें में जानते है ?<br><br>Do your co-workers know about your disease? | हा, उनमे से ज़्यादातर<br>Yes, most of them 1<br><br>हा, उनमे से कुछ<br>Yes, some of them 2<br><br>नहीं उनमे से कोइ नहीं<br>No, none of them 3<br><br>जवाब नहीं दिया<br>Will not answer -888<br><br>मालूम नहीं<br>Does not know -999 | |

## म.सामाजिक भागीदारी

## M: Social insertion

प्रश्नावली कोड/Questionnaire code: |__|__| |__|__| |__|__| |__|__|__|

| | | | |
|---|---|---|---|
| M.6 | **क्या आपको लगता है कि जब से आप बीमार हुये लोग आपसे कम मेल जोल रखते हैं ?**<br>Do you feel that ever since you have been sick, people interact less with you? | **हा, बिल्कुल** 1<br>Yes, definitely | |
| | | **हा थोड़ा** 2<br>Yes, a little | |
| | | **नहीं** 3<br>No | Skip to **M.8** |
| | | **जवाब नहीं दिया** -888<br>Will not answer | |
| | | **मालूम नहीं** -999<br>Does not know | |
| M.7 | **आपको क्या लगता है कि लोग आपसे कम मेल जोल क्यों रखते है ?**<br>Why do you think they interact less with you?<br>**जो भी लागू होता है उस पर घेरा बनाये**<br>*CIRCLE ALL THAT APPLY* | **उन्हे डर हैं कि उन्हे भी बीमारी लग जायेगी** 1<br>They are afraid of contracting the disease | |
| | | **उन्हे लगता हैं कि मैं गदा/ घृणित हूं।** 2<br>They think I am dirty / despicable | |
| | | **वे सोचते हैं कि मैंने ठीक व्यवहार नही किए** 3<br>They think I misbehaved | |
| | | **वे नहीं चाहते को कोई उन्हे मुझसे मेल जोल रखते देखे।** 4<br>They don't want to be seen interacting with me | |
| | | -777<br>**अन्य: स्पष्ट करे** ____________________<br>Other (specify) | |
| | | **जवाब नहीं दिया** -888<br>Will not answer | |
| | | **मालूम नहीं** -999<br>Does not know | |
| M.8 | **क्या आप अपने समुदाय में किसी ऐसे अन्य व्यक्तियों को जानते है जिन्हें टी बी है ?**<br>Do you know other people in your community who have TB? | **हाँ, कई लोग** 1<br>Yes, several | |
| | | **हाँ एक** 2<br>Yes, one | |
| | | **नहीं** 3<br>No | |
| | | **जवाब नहीं दिया** -888<br>Will not answer | |

प्रश्नावली कोड/ Questionnaire code: |__|__| |__|__| |__|__| |__|__|__|

| | | | |
|---|---|---|---|
| N.0 | क्या आपको लगता है कि आपके घर कि स्थिति भविष्य में सुधरेगी ?<br>Do you think that your household's situation will improve in the future? | हाँ बहुत<br>Yes, a lot 1<br>हाँ थोड़ा<br>Yes, a little 2<br>नहीं चीजें वैसे ही रहेंगी<br>No, things are likely to stay the same 3<br>नहीं चीजे और खराब होंगी<br>No, things might well become worse 4<br>जवाब नही दिया<br>Will not answer -888<br>मालूम नही<br>Does not know -999 | |
| N.1 | आप अपनी और अपने परिवार का जीवन सुधारने के लिये क्या कुछ कर रहे है या करने की योजना बना रहे है ? (जैसे पैसा कमाने के लिये कुछ नया शुरु करना, घर का नवीनीकरण कराना, कुछ सामान खरीदना)<br><br>Do you have an ongoing or future project to improve your life and your family's life (for instance start a new activity to earn money, renew your house, purchase some good)? | हाँ<br>Yes 1<br>नही<br>No 2<br>जवाब नही दिया<br>Will not answer -888<br>मालूम नही<br>Does not know -999 | <br><br>Skip to **N.5**<br><br>Skip to **N.5**<br><br>Skip to **N.5** |
| N.2 | क्या योजना है ?<br>What is this project?<br><br>साक्षात्कारकर्ता:अगर कई योजनायें है तो जिसका उल्लेख सबसे पहले किया गया उसे लिखे ।<br><br>*INTERVIEWER: IF SEVERAL PROJECTS, WRITE DOWN THE FIRST THAT IS MENTIONED* | योजना<br>Project:<br><br>जवाब नहीं दिया<br>Will not answer -888<br>मालूम नहीं<br>Does not know -999 | |
| N.3 | क्या योजना पर काम शुरु हो गया है ?<br>Has the project begun? | हा<br>Yes 1<br>नही<br>No 2<br>जवाब नही दिया<br>Will not answer -888<br>मालूम नही<br>Does not know -999 | Skip to **N.5** |

## न आशावाद और खुशी
## N: Optimism and happiness

प्रश्नावली कोड/ Questionnaire code: |__|__| |__|__| |__|__| |__|__|__|

| | | | |
|---|---|---|---|
| N.4 | कब शुरु करने का सोचा है ?<br>When do you plan to start the project?<br><br>*INTERVIEWER: DO NOT PROMPT*<br>साक्षात्कारकर्ता: उत्तरदाता को ज्यादा प्रेरित न करें. | अगले महिनें, निश्चित तारीख<br>In the coming month, PRECISE DATE | 1 |
| | | अगले महिने, तारीख तय नही<br>In the coming month. UNPRECISE DATE | 2 |
| | | अगले साल निश्चित तारीख<br>In the coming year, PRECISE DATE | 3 |
| | | आने वाले साल में तारीख तय नहीं<br>In the coming year, UNPRECISE DATE | 4 |
| | | आने वाले एकाध साल में निश्चित तारीर<br>In more than a year, PRECISE DATE | 5 |
| | | आने वाले एकाध साल के बाद तारीख तय नहीं<br>In more than a year, UNPRECISE DATE | 6 |
| | | जवाब नहीं दिया<br>Will not answer | -888 |
| | | मालूम नही<br>Does not know | -999 |

| | | |
|---|---|---|
| पिछले सात दिनो के दौरन, आपको कितनी बार लगा कि.......<br>During the past 7 days, about how often did you feel… | | ज़्यादातर = 1 कभी-कभी=2 कभी नहीं=3<br>Often = 1 Sometimes = 2 Never = 3 |
| N.5 | में निराश हूँ<br>Hopeless | 1 2 3 -888 -999 |
| N.6 | हर चीज बहुत मुश्किल है<br>That everything is an effort | 1 2 3 -888 -999 |
| N.7 | सब व्यर्थ है<br>Worthless | 1 2 3 -888 -999 |
| N.8 | में शांतिपूर्ण तरीके से शुकून से हूँ<br>Relaxed and peaceful | 1 2 3 -888 -999 |
| N.9 | में खुश हूँ<br>Happy | 1 2 3 -888 -999 |
| N.10 | मुझे दूसरों से जलन होती है<br>Envious of others | 1 2 3 -888 -999 |
| N.11 | मुझे गुस्सा आया<br>Angry | 1 2 3 -888 -999 |
| N.12 | मे सो नही पाया<br>Unable to sleep | 1 2 3 -888 -999 |
| N.13 | मुझे रोने का दिल कर रहा था<br>Prone to crying | 1 2 3 -888 -999 |

## न आशावाद और खुशी
## N: Optimism and happiness

प्रश्नावली कोड/ Questionnaire code: |__|__| |__|__| |__|__| |__|__|__|

| | | | |
|---|---|---|---|
| | जब से आपने दवाई लेना शुरू की है तब से आप निम्म्लिक्थित कौन सी बातों से सेहमत हैं<br>Since you started taking the pills which of the following statements do you agree with | | |
| | | सेहमत हूँ = 1 सेहमत नहीं हूँ = 2 जवाब नहीं देना = -888 मालूम नहीं = -999<br>Agree = 1 Disagree = 2 Will not answer = -888 Doesn't Know = -999 | |
| N.14 | मैं अब ज्यादा सोता हूँ<br>I tend to sleep more now | 1 2 -888 -999 | |
| N.15 | मैं अब घर पर कम काम करता हूँ<br>I tend to work less at home/work | 1 2 -888 -999 | |
| N.16 | मै अब पडोसी और दोस्तों से कम मिलता हूँ<br>I tend to interact less with neighbours and friends | 1 2 -888 -999 | |

| | | | | |
|---|---|---|---|---|
| N.17 | क्या आप कहेंगें कि आप कुल मिलाकर खुश है ?<br><br>Overall, would you say that you are happy? | हाँ,मैं बहुत खुश हूँ।<br>Yes, I am very happy | 1 | |
| | | हाँ मैं थोड़ा बहुत खुश हूँ<br>Yes, I am somewhat happy | 2 | |
| | | नहीं मैं बहुत खुश नहीं हूँ<br>No, I am not very happy | 3 | |
| | | जवाब नही दिया<br>Will not answer | -888 | |
| | | मालूम नही<br>Does not know | -999 | |
| N.18 | संतुष्टि के वैश्विक पैमाने पर आप अपना इन दिनों का जीवन कहाँ रखेंगे? अगर सबसे उँचा पायदान बहुत अच्छे जीवन का और सबसे निचला पायदान सबसे खराब जीवन का है तो आप अपने जीवन को कहाँ रखेंगें?<br><br>How would you classify your global satisfaction regarding your life these days? If the top rung of this ladder represents very good life and the bottom rung represents very bad life, where would you place yourself?<br><br>**साक्षात्कारकर्ता:** उत्तरदाता को सीढ़ी का चित्र दिखायें और उस संख्या पर गोला लगाये जिसे उत्तरदाता ने चुना है ।<br>*INTERVIEWER: SHOW THE RESPONDENT THE PICTURE OF THE LADDER AND CIRCLE THE NUMBER THAT CORRESPONDS TO THE NUMBER THEY CHOOSE.* | सबसे उपर का पायदान<br>Top rung | 10 | |
| | | | 9 | |
| | | | 8 | |
| | | | 7 | |
| | | | 6 | |
| | | | 5 | |
| | | | 4 | |
| | | | 3 | |
| | | | 2 | |
| | | सबसे नीचे का पायदान<br>Bottom rung | 1 | |
| | | जवाब नही दिया<br>Will not say | -888 | |
| | | मालूम नही।<br>Does not know | -999 | |

## ओ.तम्बाकू उपभोग प्रश्नावली कोड/Questionnaire code: |__|__| |__|__| |__|__| |__|__|__|

## O: Tobacco Use

**साक्षात्कारकर्ता अब मैं आपसे आपके तंबाकू के प्रयोग के बारे में कुछ सवाल पूछना चाहूँगा।**

***INTERVIEWER: Now I am going to ask a few questions about your use of tobacco.***

| | | | |
|---|---|---|---|
| O.0 | **क्या पिछले तीस दिनों में आपने सिगरेट, बीड़ी या हुक्का पीया है ?**<br>Have you smoked cigarettes or bidis or hukkas in the last 30 days? | **हाँ** Yes 1 | |
| | | **नहीं** No 2 | Skip to O**.3** |
| | | **जवाब नहीं दिया** Will not answer -888 | Skip to O**.3** |
| | | **मालूम नहीं** Does not know -999 | Skip to O**.3** |
| O.1 | **आपने पिछले चौबीस घंटो में कितनी बीड़ी/सिगरेट/हुक्का पी ?**<br>How many cigarettes/bidis/hukkas did you smoke in the last 24 hours? | **संख्या लिखें** *RECORD NUMBER* [ ][ ] 1 | |
| | | **जवाब नहीं दिया** Will not answer -888 | |
| | | **मालूम नहीं** Does not know -999 | |
| O.2 | **पिछले तीस दिनों में आपने सिगरेट/बीड़ी/हुक्का पर कितना खर्च किया ?**<br>How much have you spent on cigarettes/bidis/hukkas in the last 30 days? | **राशि लिखें** रू./ Rs *RECORD AMOUNT* [ ][ ][ ] 1 | |
| | | **जवाब नहीं दिया** Will not answer -888 | |
| | | **मालूम नहीं** Does not know -999 | |
| O.3 | **क्या पिछले तीस दिनों में आपने खैनी, तंबाकू या गुटखा खाया है ?**<br>Have you chewed khaini or tobacco or gudakhu in the last 30 days? | **हाँ** Yes 1 | |
| | | **नहीं** No 2 | Skip to **P.0** |
| | | **जवाब नहीं दिया** Will not answer -888 | Skip to **P.0** |
| | | **मालूम नहीं** Does not know -999 | Skip to **P.0** |
| O.4 | **पिछले चौबीस घंटो में आपने कितना खैनी, तंबाकू या गुटखा खाया है ?**<br>How many khaini or tobacco or gudakhu did you chew in the last 24 hours? | **संख्या लिखें** *RECORD NUMBER* [ ][ ] 1 | |
| | | **जवाब नहीं दिया** Will not answer -888 | |
| | | **मालूम नहीं** Does not know -999 | |
| O.5 | **पिछले तीस दिनों में आपने खैनी, तंबाकू या गुटखा पर कितना खर्च किया ?**<br>How much have you spent on khaini or tobacco or gudakhu in the last 30 days? | **राशि लिखें** रू./ Rs *RECORD AMOUNT* [ ][ ][ ] 1 | |
| | | **जवाब नहीं दिया** Will not answer -888 | |
| | | **मालूम नहीं** Does not know -999 | |

| प.ऋण और बचत<br>**P: Borrowing & Saving** | प्रश्नावली कोड/<br>Questionnaire code: \|__\|__\| \|__\|__\| \|__\|__\| \|__\|__\|__\| |
|---|---|

साक्षात्कारकर्ताः मेरे सवालों का धैर्यपूर्वक जवाब देने के लिये धन्यवाद।अब में आपसे आपकी बचत और उधार संबंधी आदतों और आपके घर के सामानों के बारें में पूछना चाहूँगा ।

INTERVIEWER: Thank you for your patience in answering our questions thus far. Now I am going to ask you some questions about your household including questions about savings and borrowing habits, and goods in your house.

| | | | | |
|---|---|---|---|---|
| P.0 | क्या आपके घर के किसी भी सदस्य ने बैंक, साहूकार,गैर सरकारी संस्था या किसी दोस्त से कर्ज लिया है ?<br><br>Does anyone in this household have any debts, such as loans from banks, moneylenders, NGO or friends? | हाँ<br>Yes | 1 | |
| | | नहीं<br>No | 2 | Skip to **Q.1** |
| | | जवाब नहीं दिया<br>Will not answer | -888 | Skip to **Q.1** |
| | | मालूम नहीं<br>Does not know | -999 | Skip to **Q.1** |
| P.1 | इस घर को कितने कर्ज चुकाने है ?( इस घर ने अलग अलग कितने लोगों/ जगहों से कर्ज लिया हुआ है ?)<br><br>How many outstanding loans / debts does this household have? (How many different entities has this household borrowed money from?) | संख्या<br>*NUMBER* \|__\|__\| | 1 | |
| | | जवाब नहीं दिया<br>Will not answer | -888 | |
| | | मालूम नहीं<br>Does not know | -999 | |
| P.2 | सारे कर्जे मिलाकर आपके परिवार को कुल कितना पैसा चुकाना है ?<br>What is the total amount of money that your household has to reimburse, when considering all these loans together? | रू./Rs. \|__\|__\|__\|__\| , \|__\|__\|__\| | 1 | |
| | | जवाब नहीं दिया<br>Will not answer | -888 | |
| | | मालूम नहीं<br>Does not know | -999 | |
| P.3 | इस घर ने मुख्य तौर पर कहाँ से उधार लिया है?<br>What are the lending source of the household's loans?<br><br>साक्षात्कारकर्ताः जो भी लागू हो उन सब पर गोला लगाये<br>*INTERVIEWER: CIRCLE ALL THAT APPLIES* | बैंक<br>Bank | 1 | |
| | | गैरसरकारी संगठन<br>NGO | 2 | |
| | | स्वय सहायता समूह/ माइक्रोफ़ाइनेन्स समू<br>Self-Help Group / microfinance group | 3 | |
| | | दुकानदार<br>Shopkeeper | 4 | |
| | | समाज-बिरदारी का साहूकार<br>Moneylender in the community | 5 | |
| | | दोस्त/रिश्तेदार<br>Friends/ relatives | 6 | |
| | | अन्यः स्पष्ट करें ____________<br>Other (specify) | -777 | |
| | | जवाब नहीं दिया<br>Will not answer | -888 | |
| | | मालूम नहीं<br>Does not know | -999 | |

प्रश्नावली कोड/
Questionnaire code: |__|__| |__|__| |__|__| |__|__|__|

| | | | |
|---|---|---|---|
| P.4 | किन तरह के खर्चों के लिये आपको कर्ज लेना पड़ा?<br>To cover what kind of expenses did your household take these loans?<br><br>साक्षात्कारकर्ता: जो भी लागू हो उन सब पर गोला लगाये<br>*INTERVIEWER: CIRCLE ALL THAT APPLIES* | स्वास्थ्य<br>Health | 1 |
| | | पढाई के लिये<br>Schooling | 2 |
| | | काम संबंधी<br>Work related | 3 |
| | | शादी, तीज-त्योहार, कोई विशेष आयोजन<br>Wedding, festival, other special event | 4 |
| | | अन्य: स्पष्ट करें ____________<br>Other (specify) | -777 |
| | | जवाब नहीं दिया<br>Will not answer | -888 |
| | | मालूम नहीं<br>Does not know | -999 |
| P.5 | क्या इस घर में किसी के नाम पर बचत खाता है (चाहे वो स्वंय सहायता समूह में हो, बैंक या डाकघर में)<br><br>Does anyone in this household have any savings accounts in their name (including in SHGs, bank or post office)? | हाँ<br>Yes | 1 |
| | | नहीं<br>No | 2 |
| | | जवाब नहीं दिया<br>Will not answer | -888 |
| | | मालूम नहीं<br>Does not know | -999 |

**क.परिसंपत्ति**
**Q: Assets**

प्रश्नावली कोड/
Questionnaire code: |__|__| |__|__| |__|__| |__|__|__|

**आपके सहयोग के लिये धन्यवाद। अब मैं आपसे आपके घर की संपत्तियों के बारें में कुछ सवाल पूछना चाहता हूँ।**
*Thank you for your cooperation. Now I would like to ask some questions about your household's assets.*

**हर संपति के लिये 1या 2 पर गोला लगायें**
*FOR EACH ASSET, CIRCLE 1 OR 2.*

| | कृपया बताये कि आपके पास निम्न में से कौन-कौन सी परिसंपत्तियाँ हैं:<br>Please tell me if your household owns any of the following items: | हाँ<br>Yes | नहीं<br>No | यदि,हाँ.तो कितनी?<br>If yes, how many? | जवाब नहीं दिया<br>Will not answer | मालूम नहीं<br>Does not know |
|---|---|---|---|---|---|---|
| Q.1 | टेलीफोन(लैण्डलाईन/मोबाईल)<br>Telephone (landline / cell phone) | 1 | 2 | [ ][ ] | -888 | -999 |
| Q.2 | घड़ी<br>Clock or watch | 1 | 2 | [ ][ ] | -888 | -999 |
| Q.3 | चूल्हा(बिज़ली, गैस या केरोसीन का)<br>Stove (*electric, gas or kerosene* ) | 1 | 2 | [ ][ ] | -888 | -999 |
| Q.4 | चूल्हा (कोयले या लकड़ी का)<br>Stove (*coal or wood* ) | 1 | 2 | [ ][ ] | -888 | -999 |
| Q.5 | बिज़ली<br>Electricity | 1 | 2 | | -888 | -999 |
| Q.6 | बिज़ली का पंखा<br>Electric fan | 1 | 2 | [ ][ ] | -888 | -999 |
| Q.7 | नल(टोटी) का पानी<br>Tap water | 1 | 2 | | -888 | -999 |
| Q.8 | पेयजल के अन्य स्रोत<br>Other source of water at your place | 1 | 2 | | -888 | -999 |
| Q.9 | ब्लैक एंड व्हाईट टी वी<br>Black and white television set | 1 | 2 | [ ][ ] | -888 | -999 |
| Q.10 | रंगीन टी वी<br>Color television set | 1 | 2 | [ ][ ] | -888 | -999 |
| Q.11 | वी सी आर/ वी सी पी<br>VCR / VCP | 1 | 2 | [ ][ ] | -888 | -999 |
| Q.12 | डी वी डी प्लेयर<br>DVD player | 1 | 2 | [ ][ ] | -888 | -999 |
| Q.13 | कुकर<br>Pressure cooker | 1 | 2 | [ ][ ] | -888 | -999 |
| Q.14 | फ़्रिज<br>Refrigerator / freezer | 1 | 2 | [ ][ ] | -888 | -999 |
| Q.15 | कपड़े धोने की मशीन<br>Washing machine | 1 | 2 | [ ][ ] | -888 | -999 |
| Q.16 | एसी / कूलर<br>AC/ Cooler | 1 | 2 | [ ][ ] | -888 | -999 |
| Q.17 | जनरेटर<br>Generator | 1 | 2 | [ ][ ] | -888 | -999 |
| Q.18 | कंप्यूटर (डेस्कटॉप या लैपटॉप)<br>Computer (desktop/ labptop) | 1 | 2 | [ ][ ] | -888 | -999 |

| | | | | | |
|---|---|---|---|---|---|
| Q.19 | इन्वर्टर<br>Inverter | 1 2 | \|__\|__\| | -888 | -999 |
| Q.20 | रेडियो(ट्रांजिस्टर या स्टीरियो) - टेप<br>Radio (transistor or stereo) - cassette player | 1 2 | \|__\|__\| | -888 | -999 |
| Q.21 | सिलाई मशीन<br>Sewing machine | 1 2 | \|__\|__\| | -888 | -999 |
| Q.22 | कार या जीप<br>Car or jeep | 1 2 | \|__\|__\| | -888 | -999 |
| Q.23 | साईकल<br>Bicycle | 1 2 | \|__\|__\| | -888 | -999 |
| Q.24 | मोटरसाईकल, मोपेड या स्कूटर<br>Motorcycle, moped or scooter | 1 2 | \|__\|__\| | -888 | -999 |
| Q.25 | कुर्सी, तिपाई, मोढ़ा<br>Chair / stool | 1 2 | \|__\|__\| | -888 | -999 |
| Q.26 | खाट या बेड<br>Cot or bed | 1 2 | \|__\|__\| | -888 | -999 |
| Q.27 | मेज<br>Table | 1 2 | \|__\|__\| | -888 | -999 |
| Q.28 | साईड टेबल (किनारे वाली छोटी मेज)<br>Side tables | 1 2 | \|__\|__\| | -888 | -999 |
| Q.29 | आलमारी<br>Shelves | 1 2 | \|__\|__\| | -888 | -999 |
| Q.30 | सोफ़ा<br>Couch | 1 2 | \|__\|__\| | -888 | -999 |
| Q.31 | दरवाजे वाली आलमारी<br>Cupboard | 1 2 | \|__\|__\| | -888 | -999 |
| Q.32 | साड़ी/ सूट<br>Sarees / Suits | 1 2 | \|__\|__\| | -888 | -999 |
| Q.33 | शादी के गहनें<br>Wedding Ornament | 1 2 | \|__\|__\| | -888 | -999 |

## र: सम्पत्ति और साफ सफाई
## R: Wealth and Sanitation

प्रश्नावली कोड/
Questionnaire code: |__|__| |__|__| |__|__| |__|__|__|

***साक्षात्कारकर्ताः यह सम्पत्ति सम्बंधित प्रश्न आपके घर ज़मीन और पारिवारिक आय से जुड़े हैं***
*INTERVIEWER: These wealth questions concern your house, land you own and your family incomes.*

| | | | | |
|---|---|---|---|---|
| R.0 | **इस घर या फ़्लैट में कितने कमरें है ?**<br>How many rooms are there in this house / apartment?<br><br>**रसोई घर, बाथरूम और वेरंदाह को छोड़ कर**<br>Exclude: Kitchen, Bathroom and Veranda | **कमरों की संख्या**<br>Number of rooms [ ][ ] | 1 | |
| | | **जवाब नहीं दिया**<br>Will not answer | -888 | |
| | | **मालूम नहीं**<br>Does not know | -999 | |
| R.1 | **आप घर का गंदा पानी कहाँ जाता है ?**<br>How do you dispose wastewater?<br><br>**जो भी लागू होता है उस पर घेरा बनाये**<br>*CIRCLE ALL THAT APPLY* | **नाली में जो सीवर से जुड़ी है।**<br>In a drain linked to underground sewage | 1 | |
| | | **घर के बाहर के गटर में**<br>In a gutter outside the house | 2 | |
| | | **कूड़े के ढेर मे**<br>In a dumpyard | 3 | |
| | | बाहर सड़क पर<br>Outside on the street | 4 | |
| | | **अन्य, स्पष्ट करे** ____________<br>Other (specify) | -777 | |
| | | **जवाब नहीं दिया**<br>Will not answer | -888 | |
| | | **मालूम नहीं**<br>Does not know | -999 | |
| R.2 | **आप कूड़े को कहा डालते हो?**<br>How do you dispose garbage?<br><br>**जो भी लागू होता है उस पर घेरा बनाये**<br>*CIRCLE ALL THAT APPLY* | **एक कूड़ेदान में जिसे समय समय पर खाली किया जाता है ।**<br>In a bin collected periodically | 1 | |
| | | **कूड़े के ढेर में**<br>In a dumpyard | 2 | |
| | | **कूड़े के ढेर मे जला देते हैं**<br>Burn them in a dumpyard | 3 | |
| | | **ज़मीन मे गाड़ देते हैं ।**<br>Bury them in the soil/ landfill | 4 | |
| | | **कचरा बाहर फेंक देते है और नगरपालिका उसे साफ कराती है ।**<br>Throw waste outside the house and the muncipality cleans it up | 5 | |
| | | **अन्य स्पष्ट करे** ____________<br>Other (specify) | -777 | |
| | | **जवाब नहीं दिया**<br>Will not answer | -888 | |
| | | **मालूम नहीं**<br>Does not know | -999 | |

| | | | |
|---|---|---|---|
| R.3 | **आप बलगम को कहाँ डालते हो?**<br>How do you dispose sputum?<br><br>**जो भी लागू होता है उस पर घेरा बनाये**<br>*CIRCLE ALL THAT APPLY* | **घर के एक कोने में**<br>In a corner in the house — 1 | |
| | | **घर के लैट्रिन/ शौचालय में**<br>In the toilet/latrine in the house — 2 | |
| | | **एक थैली में जिसे बाद में घर के बाहर फेंक देते है ।**<br>In a bag and then thrown outside the house — 3 | |
| | | **एक थैली मे जिसे बाद मे जला देते हैं ।**<br>In a bag and then burn it — 4 | |
| | | **नालों मे**<br>In a drain — 5 | |
| | | **ज़मीन मे गाड़ देते हैं।**<br>Bury it — 6 | |
| | | **मैं सड़क पर थूकता हू**<br>I spit on the street — 7 | |
| | | **अन्य, स्पष्ट करें।**<br>________________<br>Other (specify) — -777 | |
| | | **जवाब नही दिया**<br>Will not answer — -888 | |
| | | **मालूम नहीं**<br>Does not know — -999 | |
| R.4 | **क्या यह मकान आपका अपना है ?**<br>Does this household own this house? | **हाँ**<br>Yes — 1 | |
| | | **नहीं**<br>No — 2 | Skip to **R.7** |
| | | **जवाब नही दिया**<br>Will not answer — -888 | Skip to **R.7** |
| | | **मालूम नहीं**<br>Does not know — -999 | Skip to **R.7** |
| R.5 | **क्या यह ज़मीन जिस पर यह मकान बना है इस परिवार की है ?**<br><br>Does this household own land on which this house is built? | **हाँ**<br>Yes — 1 | |
| | | **नहीं**<br>No — 2 | |
| | | **जवाब नही दिया**<br>Will not answer — -888 | |
| | | **मालूम नहीं**<br>Does not know — -999 | |

## र: सम्पत्ति और साफ सफाई
## R: Wealth and Sanitation

प्रश्नावली कोड/
Questionnaire code: |__|__| |__|__| |__|__| |__|__|__|

| | | | |
|---|---|---|---|
| R.6 | **इस पूरे रिहाईश जो इस परिवार की है की कुल कीमत क्या है ?(मकान और, या ज़मीन)**<br><br>What is the total value of this dwelling owned by the household? (House and / or land) | Rs./रु [ ][ ] , [ ][ ] , [ ][ ][ ] 1 | |
| | | **जवाब नहीं दिया** -888<br>Will not answer | |
| | | **मालूम नहीं** -999<br>Does not know | |
| R.7 | **क्या इस मकान की ज़मीन के अलावा इस परिवार की कहीं और भी ज़मीन और / या संपत्ति है ?**<br><br>Does this household own any land and/or property other than where this house is built? | **हाँ** 1<br>Yes | |
| | | **नहीं** 2<br>No | Skip to **R.9** |
| | | **जवाब नहीं दिया** -888<br>Will not answer | Skip to **R.9** |
| | | **मालूम नहीं** -999<br>Does not know | Skip to **R.9** |
| R.8 | **उस दूसरी ज़मीन की कुल कीमत क्या है ?**<br>What is the total value of your other land (Rs.)? | Rs./रु [ ][ ] , [ ][ ] , [ ][ ][ ] 1 | |
| | | **जवाब नहीं दिया** -888<br>Will not answer | |
| | | **मालूम नहीं** -999<br>Does not know | |
| R.9 | **क्या आपके पास कोई मवेशी जैसे जानवर, बकरी या चिड़िया है ?**<br><br>Does this household own any livestock, such as cattle, goats or birds? | **हाँ** 1<br>Yes | |
| | | **नहीं** 2<br>No | Skip to **R.11** |
| | | **जवाब नहीं दिया** -888<br>Will not answer | Skip to **R.11** |
| | | **मालूम नहीं** -999<br>Does not know | Skip to **R.11** |
| R.10 | **इस घर के मवेशियों की कुल कीमत क्या होगी ?**<br>What is the total value of your household's livestock? | Rs./रु [ ][ ] , [ ][ ] , [ ][ ][ ] 1 | |
| | | **जवाब नहीं दिया** -888<br>Will not answer | |
| | | **मालूम नहीं** -999<br>Does not know | |

| | | | | |
|---|---|---|---|---|
| R.11 | **क्या आप के यहाँ खेती बाड़ी की उपज भी आती है ? ( चाहे खुद करे , साझे में करे या किराये पर हो )**<br><br>Does this household receive agricultural produce? (whether self-employed or as share or rent payment) | **हाँ**<br>Yes | 1 | |
| | | **नहीं**<br>No | 2 | Skip to **R.13** |
| | | **जवाब नहीं दिया**<br>Will not answer | -888 | Skip to **R.13** |
| | | **मालूम नहीं**<br>Does not know | -999 | Skip to **R.13** |
| R.12 | **आपके घर के कृषि उपज की कुल कीमत क्या है ?**<br>What is the total value of your household's agricultural produce? | Rs./रु [ ][ ] , [ ][ ] , [ ][ ][ ] | 1 | |
| | | **जवाब नहीं दिया**<br>Will not answer | -888 | |
| | | **मालूम नहीं**<br>Does not know | -999 | |
| R.13 | **पिछले बारह महीनों में आपके घर की औसत आमदनी क्या रही ?**<br><br>Over the past 12 months, what was the monthly income of the household, on average? | **500 रू. से कम**<br>Less than 500 Rs. | 1 | |
| | | **500 से 1000 रू.**<br>500 to 1000 Rs. | 2 | |
| | | **1000 से 1500 रू.**<br>1000 to 1500 Rs. | 3 | |
| | | **1500 से 2000 रू.**<br>1500 to 2000 Rs. | 4 | |
| | | **अन्य, स्पष्ट करें** ____________<br>Other (specify) | -777 | |
| | | **जवाब नहीं दिया**<br>Will not answer | -888 | |
| | | **मालूम नहीं**<br>Does not know | -999 | |

# स: व्यय
# S: Consumption

प्रश्नावली कोड/
Questionnaire code: |__|__| |__|__| |__|__| |__|__|__|

**साक्षात्कारकर्ता:अब में आपसे पिछले तीस दिनों के अंतर्गत व्यय के बारे में पूछूँगा चाहे वह खरीदारी हो या घरेलु उत्पादन हो**
INTERVIEWER: I would like to ask you about your consumption in the last 30 days, whether it comes from purchasing or production at home.

| | | व्यय का मूल / रु Value of consumption (Rs.) | | |
|---|---|---|---|---|
| | | पिछले 30 दिनों में Last 30 days | जवाब नहीं दिया Will not answer | मालूम नहीं Does not know |
| S.0 | **अनाज और अनाज संबंधी उत्पाद** Cereals & cereal products | \|__\|__\|__\|__\| Rs | -888 | -999 |
| S.1 | **दाल और दाल संबंधी उत्पाद** Pulses & pulse products | \|__\|__\|__\|__\| Rs | -888 | -999 |
| S.2 | **दूध और दूध संबंधी उत्पाद** Milk & milk products | \|__\|__\|__\|__\| Rs | -888 | -999 |
| S.3 | **खाद्य तेल** Edible oil | \|__\|__\|__\|__\| Rs | -888 | -999 |
| S.4 | **सब्जियाँ** Vegetables | \|__\|__\|__\|__\| Rs | -888 | -999 |
| S.5 | **फ़ल और मेवे** Fruits & nuts | \|__\|__\|__\|__\| Rs | -888 | -999 |
| S.6 | **अंडा, मछली और माँस** Egg, fish & meat | \|__\|__\|__\|__\| Rs | -888 | -999 |
| S.7 | **अन्य खाद्य पदार्थ** Other food items | \|__\|__\|__\|__\| Rs | -888 | -999 |
| S.8 | **पान, तंबाकू और अन्य नशे के पदार्थ** Paan, tobacco & intoxicants | \|__\|__\|__\|__\| Rs | -888 | -999 |
| S.9 | **ईंधन** Fuel & light | \|__\|__\|__\|__\| Rs | -888 | -999 |
| S.10 | **कुल-टोटल (आइटम 1-9) SUB-TOTAL (ITEM 1 - 9)** | \|__\|__\|__\|__\|__\| Rs | | |
| | अन्य पदार्थ और सेवायें (मासिक खर्च) MISCELLANEOUS GOODS & SERVICES (MONTHLY EXPENDITURE) | | | |
| S.11 | **सिनेमा/नाटक/वीडियो/ केबल** Cinema / theatre / video show / cable TV | \|__\|__\|__\|__\| Rs | -888 | -999 |
| S.12 | **अखबार, पत्रिका, किताबें (पढ़ाई की नहीं)** Newspapers, magazines, books (not textbooks) | \|__\|__\|__\|__\| Rs | -888 | -999 |
| S.13 | **स्वास्थय-ख़र्च (ग़ैर-संस्थानिक)** Medical expenses (non-institutional) | \|__\|__\|__\|__\| Rs | -888 | -999 |
| S.14 | **शौचालय** के लिए इस्तेमाल होने वाली वस्तुएं जैसे साबुन, शम्पू और अन्य सफाई के पदार्थ Toilet articles including washing soap, shampoo & other cleaning agents | \|__\|__\|__\|__\| Rs | -888 | -999 |
| S.15 | यात्रा तथा ईंधन खर्च Travel including commuting and fuel | \|__\|__\|__\|__\| Rs | -888 | -999 |
| S.16 | घर का किराया House rent | \|__\|__\|__\|__\| Rs | -888 | -999 |
| S.17 | **मोबाईल रिचार्ज** Mobile re-charge | \|__\|__\|__\|__\| Rs | -888 | -999 |
| S.18 | अन्य **विभिन्न** वस्तुएं और सेवाएं Other miscellaneous goods & services | \|__\|__\|__\|__\| Rs | -888 | -999 |
| S.19 | **कुल-टोटल (आइटम 11-17) SUB-TOTAL (ITEM 11 - 18)** | \|__\|__\|__\|__\|__\| Rs | | |

## स: व्यय
## S: Consumption

प्रश्नावली कोड/
Questionnaire code: |__|__| |__|__| |__|__| |__|__|__|

| | | | | | |
|---|---|---|---|---|---|
| | **विभिन्न वस्तुएँ** और सेवाएं (सालाना खर्च)<br>MISCELLANEOUS GOODS & SERVICES (ANNUAL EXPENDITURE) | | | | |
| S.20 | ट्यूशन फीस<br>Tuition Fees | -888 | -999 | | Rs |
| S.21 | स्कूल की पुस्तकें और अन्य शैक्षिक खर्च<br>School books & other education expenses | -888 | -999 | | Rs |
| S.22 | अस्पताल और निजी स्वास्थ्य केंद्र **(संस्थानिक)**<br>Hospital & nursing home (institutional) | -888 | -999 | | Rs |
| S.23 | कपड़े / जुते / **चप्पलें**<br>Clothing / Footwear | -888 | -999 | | Rs |
| S.24 | फर्नीचर<br>Furniture | -888 | -999 | | Rs |
| S.25 | बर्तन / रसोई का सामान<br>Utensils / Kitchen equipment | -888 | -999 | | Rs |
| S.26 | **गाड़ियाँ**/मोटर साईकल /साईकल<br>Vehicles / motorcycles / bicycle | -888 | -999 | | Rs |
| S.27 | टीवी, रेड़ियो **आदि**<br>TV, radio, etc | -888 | -999 | | Rs |
| S.28 | घर के अन्य सामान<br>Other household appliances | -888 | -999 | | Rs |
| S.29 | मर्रमत और रखरखाव<br>Repair & maintenance | -888 | -999 | | Rs |
| S.30 | तैयोहार, **छुट्टियों,** शदियो, उपहार देन **के** खर्च<br>Expenses for festivals, holidays, vacation, weddings, gifts | -888 | -999 | | Rs |
| S.31 | **कुल-टोटल (आइटम 20-30) SUB-TOTAL (ITEM 20 - 30)** | | | | Rs |

## ट: माप
## T: Measurements

प्रश्नावली कोड/
Questionnaire code: |__|__| |__|__| |__|__| |__|__|__|

**साक्षात्कारकर्ता /अत मे मैं आप से कुछ चीजो की माप के बारे मे पूछना चाहूंगा**
*INTERVIEWER: Finally, I would like to take a few measurements.*

| | | | |
|---|---|---|---|
| T.0 | **वजन (किलो)**<br>*WEIGHT (Kilograms)*<br><br>साक्षात्कारकर्ता :नापे और लिखे<br>*INTERVIEWER: Measure and record* | [ ][ ][ ][ ] . [ ][ ] KGs /**किला** 1<br>**भाग नहीं लूँगा** Will not participate -888<br>**नहीं कर सकते** Cannot perform -999 | |
| T.1 | **लंबाई (सेमी.)**<br>*HEIGHT (Centimeters)*<br><br>*INTERVIEWER: Measure and record*<br>साक्षात्कारकर्ता :नापे और लिखे | [ ][ ][ ][ ] . [ ][ ] CMs/ **समा.** 1<br>**भाग नहीं लूँगा** Will not participate -888<br>**नहीं कर सकते** Cannot perform -999 | |
| T.2 | **उपरी बाँह (प्रगण्ड) की परिधि(सेमी.)**<br>*UPPER ARM CIRCUMPHERANCE (Centimeters)*<br><br>*INTERVIEWER: Measure and record*<br>साक्षात्कारकर्ता :नापे और लिखे | [ ][ ][ ][ ] . [ ][ ] CMs/ **समा.** 1<br>**भाग नहीं लूँगा** Will not participate -888<br>**नहीं कर सकते** Cannot perform -999 | |

प्रश्नावली कोड/
Questionnaire code: |__|__| |__|__| |__|__| |__|__|__|

| | | | |
|---|---|---|---|
| U.0 | **सर्वे समाप्त होने का समय**<br>*ENDING TIME OF THE SURVEY* | घंटे<br>Hour [ ][ ]   मिनट<br>Minute [ ][ ] | |
| U.1 | **क्या सर्वे के समय और लोग भी मौजूद थे? यदि हाँ तो मरीज के साथ उनका रिश्ता लिखिये (जैसे पिता, बच्चा, पड़ोसी) ?**<br><br>Were other people present during this survey? If yes, indicate not their name but their relation to the patient (e.g.: father / child / neighbor / …) | **हाँ**<br>Yes 1<br>**व्यक्ति 1**<br>Person 1<br>**व्यक्ति 2**<br>Person 2<br>**व्यक्ति 3**<br>Person 3<br>**व्यक्ति 4**<br>Person 4<br>**व्यक्ति 5**<br>Person 5<br>**व्यक्ति 6**<br>Person 6<br>**कोई नहीं**<br>None 2 | |
| U.2 | **क्या सर्वे करने में कोई समस्या आई थी?**<br>Were there any problems administering the survey? | **हाँ**<br>Yes 1<br>**नहीं**<br>No 2<br>**टिप्पणा**<br>Remarks: | |
| U.3 | **आपके अनुसार उत्तरदाता से मिली जानकारी कितनी अच्छि/सही थी?**<br><br>Would you say that the quality of the respondent's answers were … | **बहुत अच्छा**<br>Very good 1<br>**अच्छा**<br>Good 2<br>**संतोषजनक**<br>Satisfying 3<br>**खराब**<br>Bad 4<br>**बहुत खराब**<br>Very bad 5<br>**कहना मुश्किल**<br>Difficult to say 6 | |
| U.4 | **क्या सर्वे के दौरान आपके सुपरवाईजर मौजूद थे ?**<br>Was your supervisor present during the survey? | **हाँ**<br>Yes 1<br>**नहीं**<br>No 2 | |
| U.5 | **अंतिम टिप्पणी**<br>Final Remarks | | |

***सर्वे का अंत***
*END OF THE SURVEY*

| व: स्क्रूटिनी - बेक-चेक - एंट्री<br>**V: Scrutiny - Backcheck - Entry** | प्रश्नावली कोड/<br>Questionnaire code: \|__\|__\| \|__\|__\| \|__\|__\| \|__\|__\|__\| | | |
|---|---|---|---|
| *केवल जाचकतो द्वारा भरती के लिए*<br>*TO BE FILLED BY THE SCRUTINIZER ONLY* | | | |
| V.1 | **क्या प्रश्नावली की जाँच के गयी थी ?**<br>Was the questionnaire scrutinized? | **हाँ** Yes 1<br>**नहीं** No 2 | → **END** |
| V.2 | **यदि हाँ तो जॉचकर्ता का नाम**<br>If yes, scrutinizer's name | | |
| V.3 | **जाँच कब की गयी थी ?**<br>When was it scrutinized? | **दिन** day [ ][ ] **महिना** month [ ][ ] **साल** year [ ][ ][ ][ ] | |
| V.4 | **जाँचकर्ता की टिप्पणी**<br>Scrutinizer's Remarks | | |
| *बेक चेक जाचकतो द्वारा भारती के लिए*<br>*TO BE FILLED BY THE BACK-CHECKER ONLY* | | | |
| V.4 | **क्या प्रश्नावली की दुबारा जांच की गयी थी ?**<br>Was the questionnaire back-checked? | **हां** Yes 1<br>**नही** No 2 | → **END** |
| V.5 | **यदि हाँ तो जाँचकर्ता का नाम**<br>If yes, back-checker's name | | |
| V.6 | **दुबारा जांच कब की गयी थी ?**<br>When was it back-checked? | **दिन** day [ ][ ] **महिना** month [ ][ ] **साल** year [ ][ ][ ][ ] | |
| V.7 | **जांचकतो का नाम**<br>Back-checker's Remarks | | |
| *डाटा एंट्री ऑपरेटर द्वारा भर्ती के लिए*<br>*TO BE FILLED BY THE DATA-ENTRY OPERATORS ONLY* | | | |
| *पहली एंट्री*<br>***FIRST ENTRY*** | | | |
| V.8 | **डाटा एन्ट्री ऑपरेटर का नाम**<br>Data entry operator's name | | |
| V.9 | **इस सर्वे की इन्ट्री कब की गयी थी**<br>When was this survey entered? | **दिन** day [ ][ ] **महिना** month [ ][ ] **साल** year [ ][ ][ ][ ] | |
| V.10 | **डाटा इंट्री ऑपरेटर की टिप्पणी**<br>Data-entry operator's Remarks | | |
| *दूसरा एंट्री*<br>***SECOND ENTRY*** | | | |
| V.11 | **डाटा इंट्री ऑपरेटर का नाम**<br>Data entry operator's name | | |
| V.12 | **इस सर्वे की इन्ट्री कब की गयी थी**<br>When was this survey entered? | **दिन** day [ ][ ] **महिना** month [ ][ ] **साल** year [ ][ ][ ][ ] | |
| V.13 | **डाटा इंट्री ऑपरेटर की टिप्पणी**<br>Data-entry operator's Remarks | | |

## W. Notes

प्रश्नावली कोड/
Questionnaire code: |__|__| |__|__| |__|__| |__|__|__|

| Question number | Note |
| --- | --- |
| | |
| | |
| | |
| | |
| | |
| | |
| | |
| | |
| | |
| | |
| | |
| | |
| | |
| | |

**ज़: सीढ़ी**

**Z: Ladder** प्रश्नावली कोड/Questionnaire code: |__|__| |__|__| |__|__| |__|__|__|

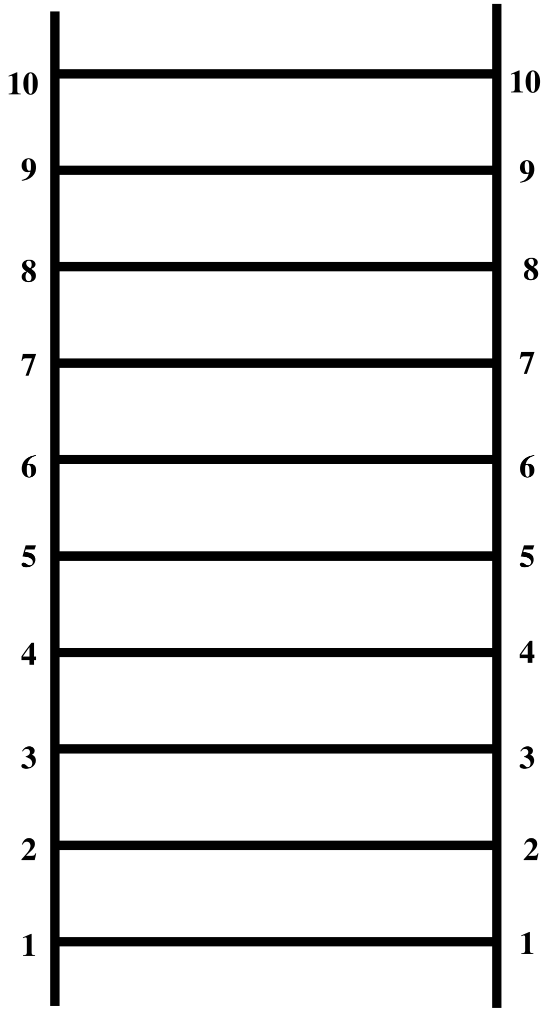

Supplement: S1 Fig — Full questionnaire administered to patients. (PDF) [file pone.0206245.s001.pdf]
